# Heteropolymetallic [FeFe]-Hydrogenase Mimics: Synthesis and Electrochemical Properties

**DOI:** 10.1021/acs.inorgchem.2c03355

**Published:** 2023-02-13

**Authors:** Alejandro Torres, Alba Collado, Mar Gómez-Gallego, Carmen Ramírez de Arellano, Miguel A. Sierra

**Affiliations:** † Departamento de Química Orgánica I, Facultad de Química, Universidad Complutense, 28040 Madrid, Spain; ‡ Center for Innovation in Advanced Chemistry (ORFEO-CINQA), Facultad de Química, Universidad Complutense, 28040 Madrid, Spain; § Departamento de Química Orgánica, Universidad de Valencia, 46100 Valencia, Spain

## Abstract

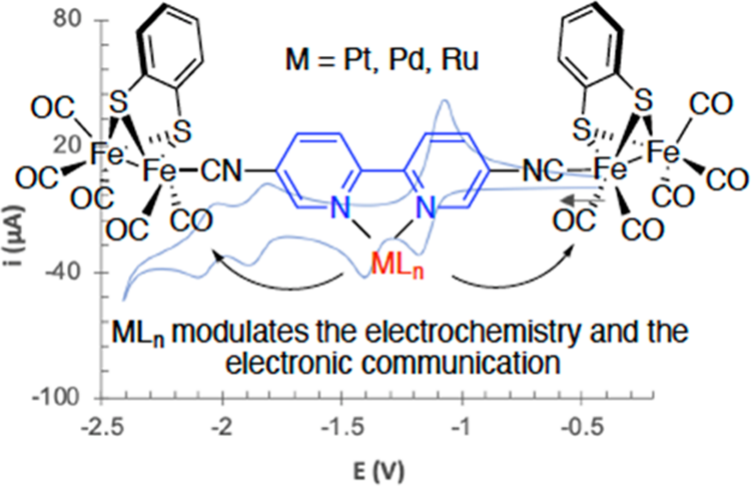

The synthesis and electrochemical properties of tetranuclear [Fe_2_S_2_]-hydrogenase mimic species containing Pt(II), Ni(II), and Ru(II) complexes have been studied. To this end, a new tetranuclear [Fe_2_S_2_] complex containing a 5,5′-diisocyanide-2,2′-bipyridine bridging ligand has been designed and coordinated to the metal complexes through the bipyridine moiety. Thus, the tetranuclear [Fe_2_S_2_] complex (**6**) coordinates to Pt(II), Ni(II) and Ru(II) yielding the corresponding metal complexes. The new metal center in the bipyridine linker modulates the electronic communication between the redox-active [Fe_2_S_2_] units. Thus, electrochemical studies and DFT calculations have shown that the presence of metal complexes in the structure strongly affect the electronic communication between the [Fe_2_S_2_] centers. In the case of diphosphine platinum compounds **10**, the structure of the phosphine ligand plays a crucial role to facilitate or to hinder the electronic communication between [Fe_2_S_2_] moieties. Compound **10a**, bearing a dppe ligand, shows weak electronic communication (Δ*E* = 170 mV), whereas the interaction is much weaker in the Pt-dppp derivative **10b** (Δ*E* = 80 mV) and virtually negligible in the Pt-dppf complex **10c**. The electronic communication is facilitated by incorporation of a Ru-bis(bipyridine) complex, as observed in the BF_4_ salt **12** (Δ*E* = 210 mV) although the reduction of the [FeFe] centers occurs at more negative potentials. Overall, the experimental–computational procedure used in this work allows us to study the electronic interaction between the redox-active centers, which, in turn, can be modulated by a transition metal.

## Abstract 2

The synthesis and electrochemical properties of tetranuclear [Fe_2_S_2_]-hydrogenase mimic species bridged by bipy-Pt(II), Ni(II), and Ru(II) complexes have been studied. An experimental−computational approach has been used to determine the electronic interaction between the redox-active centers, which are modulated by the transition metal in the bridge.

## Introduction

**Figure 1:**
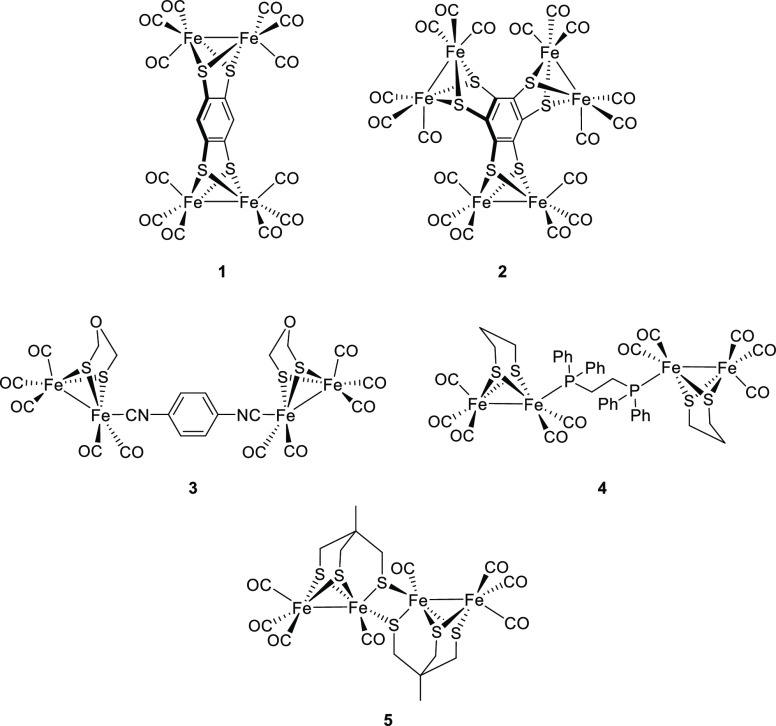
Examples of tetranuclear (**1**, **3**, **4**, and **5**) and hexanuclear (**2**) iron–sulfur complexes.

[FeFe]-Hydrogenases are enzymes capable of reversible conversion of H^+^ into H_2_.^1−5^ Due to the relevance of these transformations, a large number of studies on the [FeFe]-hydrogenase catalytic cycle have been carried out in the last decades, inspiring the efforts of synthetic chemists to design structural and functional models of these enzymes.^6−8^ Among the different types of [FeFe]-hydrogenase mimics, polynuclear iron–sulfur complexes (that is, complexes having several [Fe_2_S_2_] units), have attracted attention as they can exhibit electronic properties different from those of their mononuclear analogues.^9−11^ In these polynuclear systems, the connection of the [Fe_2_S_2_] moieties has been carried out either by covalent bonding through the substituents on the bridging sulfur ligands (**1**, **2**, and **5** in Figure 1)^10−12^ or by incorporation of the linker as a ligand in the Fe atoms (**3** and **4** in Figure 1).^9,13^

Particularly interesting to us are those polynuclear systems having spacers that allow the electronic communication between the two [Fe_2_S_2_] centers as they can work as stable multi-electron relays. In this regard, the [FeFe] centers in complexes **1** and **2**, bearing a rigid conjugate linker between the [Fe_2_S_2_] units, undergo two (**1**) or three (**2**) consecutive reversible two-electron metal-center based reductions, the reduced species being stabilized by delocalization of negative charges over the conjugate system. Additionally, complex **1** shows a good electrocatalytic behavior in the reduction of protons for ClCH_2_COOH.^11^ The electronic interaction between the [FeFe] centers is also possible through more flexible conjugate linkers, such as the diisonitrile bridging ligand in **3**.^9^

**Figure 2:**
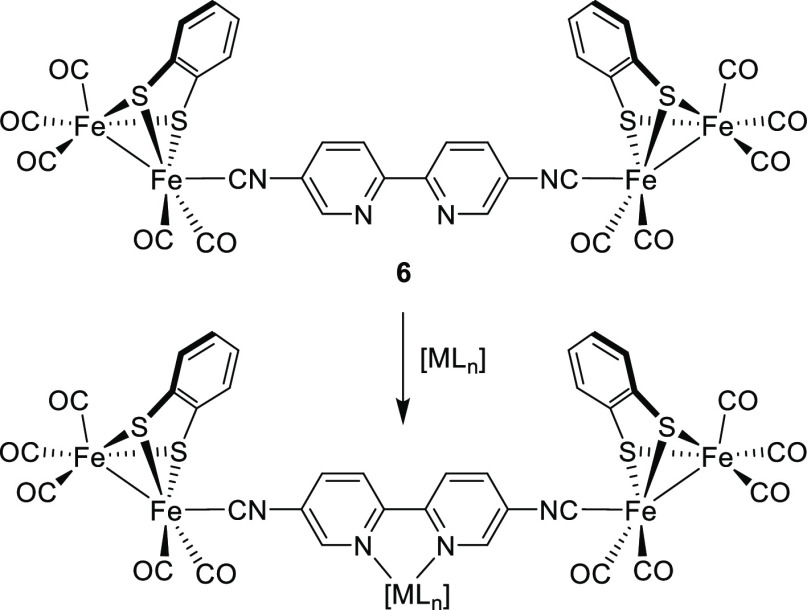
Strategy to incorporate metal complexes into a tetranuclear [Fe_2_S_2_]_2_-complex.

However, despite the interest of polynuclear [Fe_2_S_2_] complexes as models to design new robust [FeFe]-hydrogenase mimics, it is remarkable that their potential had been little explored. In this context, the linkers reported to join the [Fe_2_S_2_] units are mere connectors between the electrochemically active diiron moieties, and, as far as we know, they have not been conceived for further modifications or reactivity studies. In our current research, we are interested in the development of methodologies for the incorporation of [FeFe]-hydrogenase mimics into diverse types of molecules.^14−16^ Here, our approach focuses on the design of a polynuclear complex **6** with an active linker, suitable to facilitate the electronic communication between the [FeFe] centers and also able to act as a ligand to incorporate transition metal complexes in the structure (Figure 2). The structure of **6** combines two [Fe_2_S_2_] units, known to be electrocatalytically active for hydrogen production, with a π-conjugated linker that also has the chelating properties of the 2,2′-bipyridine moiety, a ligand widely employed in transition-metal coordination chemistry. Complex **6** will be used as a scaffold to synthesize a series of tetranuclear [FeFe]-hydrogenase mimics built as a part of square planar (Pt, Ni) and octahedral (Ru) complexes (Figure 2). The influence of the incorporation of these metal cations in the linker on the electrochemical properties of the complexes and the effect on the electronic communication between the [FeFe]-centers will be presented. The results shown here are a first step into the development of new methodologies to incorporate polynuclear iron–sulfur complexes, mimetics of [FeFe]-hydrogenases, in a wide variety of metal complexes.

## Results and Discussion

**Scheme 1:**
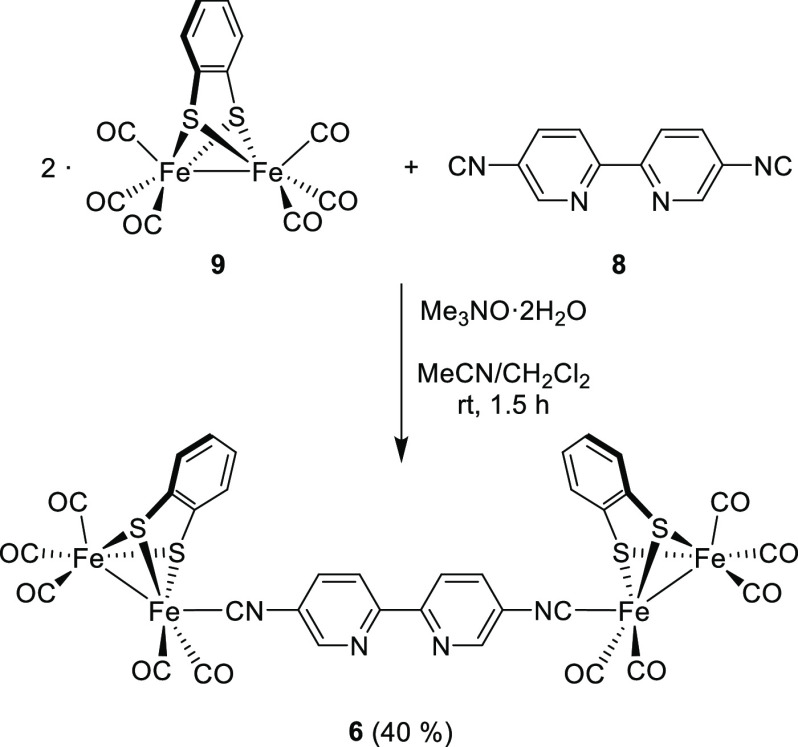
Synthesis of the Tetranuclear [Fe_2_S_2_] Complex **6**

5,5’-Diisocyanide-2,2′-bipyridine (**8**) was prepared in 74% yield from 5,5′-diamino-2,2′-bipyridine (**7**)^17^ by formylation with formic acetic anhydride followed by dehydration with POCl_3_/NEt_3_ (Scheme S1). The coordination of **8** to [(μ-bdt)][Fe(CO)_3_]_2_ (**9**) (bdt = 1,2-benzenedithiolate) was achieved by a Me_3_NO·2H_2_O-promoted CO substitution reaction. The tetranuclear complex **6** was obtained as a red solid in 40% yield after purification through a silica gel flash chromatography (Scheme 1).

**Figure 3:**
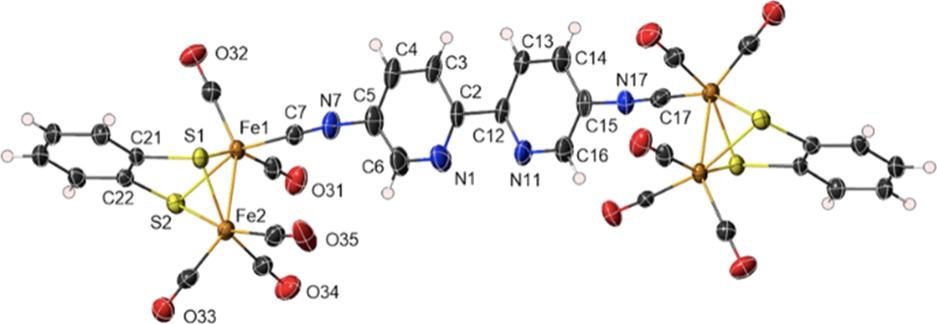
X-ray thermal ellipsoid plot of **6** (50% probability level) with the labeling scheme. Selected bond lengths (Å) and angles (°): Fe(1)-C(7) 1.786(16), Fe(1)-S(1) 2.2718(15), Fe(1)-S(2) 2.2768(13), Fe(1)-Fe(2) 2.4651(9), Fe(2)-S(1) 2.2625(13), Fe(2)-*S*(2) 2.2793(14), *S*(1)-C(21) 1.785(5), *S*(2)-C(22) 1.789(5), C(7)-N(7) 1.171(18), N(7)-C(5) 1.396(16), C(15)-N(17) 1.406(15), N(17)-C(17) 1.172(17), C(7)-Fe(1)-S(1) 90.9(7), C(7)-Fe(1)-*S*(2) 152.7(5), *S*(1)-Fe(1)-S(2) 80.39(5), C(7)-Fe(1)-Fe(2) 96.3(6), *S*(1)-Fe(2)-S(2) 80.54(5), Fe(2)-*S*(1)-Fe(1) 65.87(4), Fe(1)-*S*(2)-Fe(2) 65.51(4), N(7)-C(7)-Fe(1) 177(3), C(7)-N(7)-C(5) 172(3).

Complex **6** was structurally characterized by FTIR and NMR spectroscopy and mass spectrometry. The most relevant signals in the ^13^C{^1^H} NMR spectrum in CDCl_3_ were the singlets found at 210.6 and 209.0 ppm corresponding to the inequivalent CO ligands and the signal at 175.7 ppm corresponding to the coordinated CN-R moiety.^18,19^ The IR spectrum of **6** showed the stretching frequencies corresponding to the CN (2115 cm^–1^) and CO bonds (2077, 2038, and 1979 cm^–1^). The structure and connectivity of complex **6** was unambiguously determined by single-crystal X-ray diffraction analysis (Figure 3).

The structure shows a 5,5′-diisocyanide-2,2′-bipyridine ligand bridging two symmetry-related [(μ-SR)_2_Fe_2_(CO)_5_] units. The Fe-Fe bond length for **6** (2.4651(9) Å) lies in the range found for μ-benzene-1,2diothilate-dirion moieties (2.44–2.57 Å).^20^ The diisocyanide-bipyridine bridging ligand is bonded to the iron centers in a slightly distorted linear geometry with a Fe(1)-C(7) bond length of 1.786(16) Å and Fe(1)-C(7)-N(7) angle of 177(3)°.

**Scheme 2:**
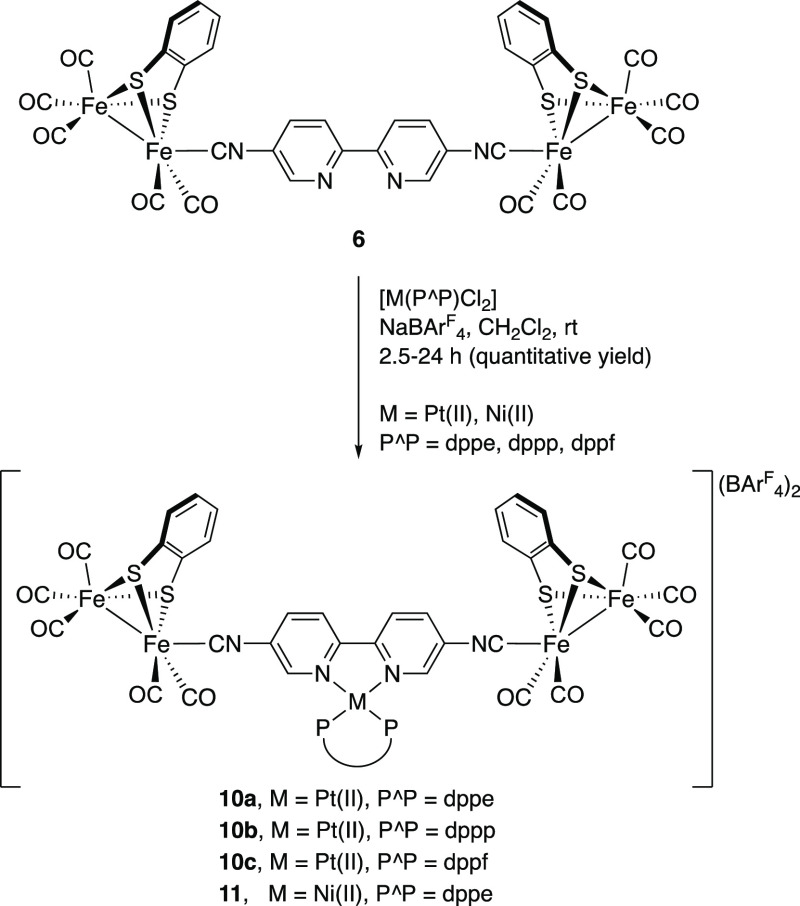
Synthesis of [FeFe]-Pt (**10**) and [FeFe]-Ni (**11**) complexes

The ability of the tetranuclear complex **6** to coordinate to Pt(II) and Ni(II) centers through the bipyridine moiety was evaluated next. Reaction of complex **6**, NaBAr^F^_4_, and the corresponding square-planar [Pt(P^P)Cl_2_] complexes [(P^P) = 1,2-bis(diphenylphosphino)ethane (dppe), 1,3-bis(diphenylphosphino)propane (dppp), 1,1′-bis(diphenylphosphino)ferrocene (dppf)] in CH_2_Cl_2,_ at room temperature, afforded compounds **10a–10c** as BAr^F^_4_ salts. These compounds were isolated as dark purple solids in quantitative yields (Scheme 2). Following the same protocol, employing [Ni(dppe)Cl_2_] as a reagent, compound **11** was isolated as a dark purple solid in quantitative yield (Scheme 2).

Compounds **10a**–**10c** and **11** were fully characterized by ^1^H, ^13^C{^1^H}, ^31^P{^1^H} and ^19^F{^1^H} NMR and IR spectroscopies and elemental analysis. The signal corresponding to the coordinated CN moiety was found in the 188.3–189.3 ppm range in the ^13^C{^1^H} NMR spectra in CDCl_3_ of compounds **10a**–**10c** and **11**, that is, significantly downfield shifted with respect to complex **6**. Accordingly, the CN stretching frequency of **10a**–**10c** and **11** in the IR spectra also shifts toward lower wavenumbers (2087–2090 cm^–1^ vs 2115 cm^–1^). One singlet was observed in the ^13^C{^1^H} NMR spectrum at ca. 208 ppm, assigned to the Fe(CO)_3_ groups. Additionally, broad signals were observed at a very similar chemical shift, which could be tentatively assigned to the CO ligands in the Fe(CO)_2_CNR-fragments. The broadness of these signals suggests restricted ligand rotation around the Fe center.^18,19^ The ^31^P{^1^H} spectra of compounds **10a**–**10c** contained a singlet at 43.77, −2.62, and 12.87 ppm, respectively, with the expected satellites due to ^31^P-^195^Pt coupling (*J*_P–Pt_ ≈ 3300 Hz). The coupling constant is in agreement with the expected *cis*-square planar geometry with the employed bisphosphines.^21^ The ^31^P{^1^H} spectra of **11** contained a singlet at 67.65 ppm.^22^

**Scheme 3:**
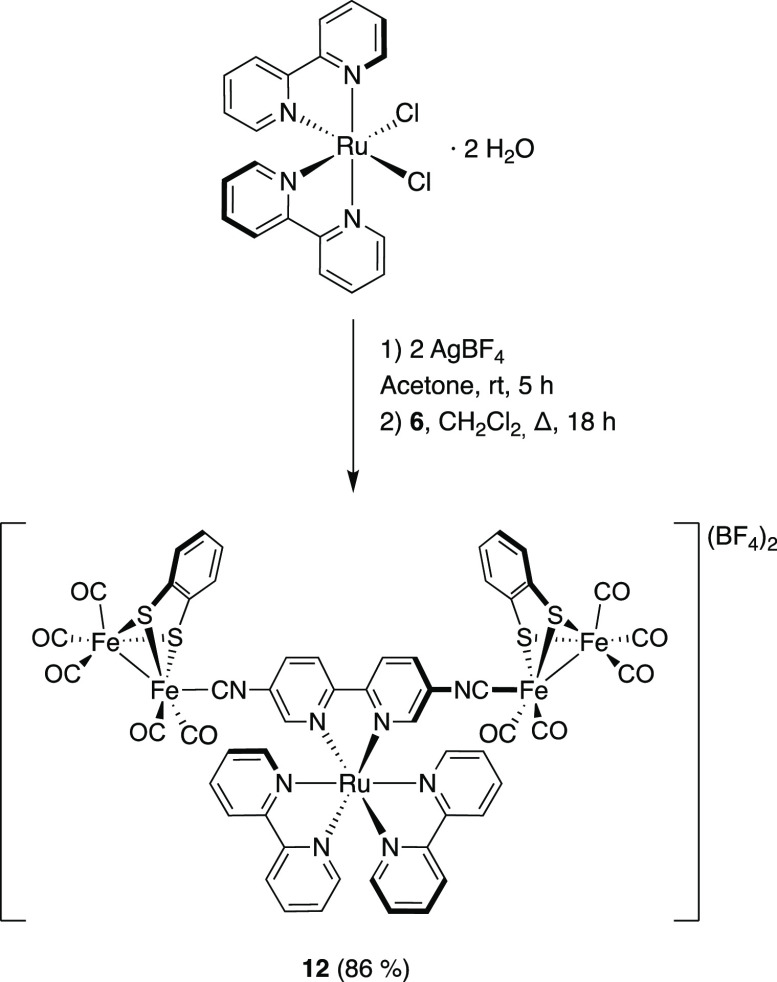
Synthesis of the [FeFe]-Ru Compound (**12**)

Additionally, the preparation of the octahedral ruthenium compound **12** was achieved by in situ generation of the bis-acetone-*solvato* salt [Ru(bpy)_2_(OCMe_2_)_2_][BF_4_]_2_ and subsequent reaction with **6** at rt. **12** was obtained as a BF_4_ salt, an orange solid in 86% yield, after flash chromatography on Al_2_O_3_ (Scheme 3), and it was characterized by ^1^H, ^13^C{^1^H}, and ^19^F{^1^H} NMR spectroscopies in CD_3_CN, IR spectroscopy, and HRMS. Two singlets were observed for the inequivalent CO-ligand C atoms at 211.3 and 209.9 ppm, and the signal corresponding to the CN ligand was found at 178.1 ppm. The ^19^F{^1^H} NMR spectrum showed the typical isotopic pattern corresponding to the presence of the BF_4_ anion at −152.12 (^10^BF_4_^–^) and −152.20 ppm (^11^BF_4_^–^). The presence of the CN, CO, and BF_4_ moieties was further confirmed by IR analysis. The CN stretching frequency appeared at 2109 cm^–1^, closer to that observed for complex **6** (2115 cm^–1^) than that obtained for the Pt and Ni complexes (ca. 2090 cm^–1^). These values suggest a very different electronic effect depending on the metal complex employed.

### Electrochemical and DFT Studies

**Figure 4:**
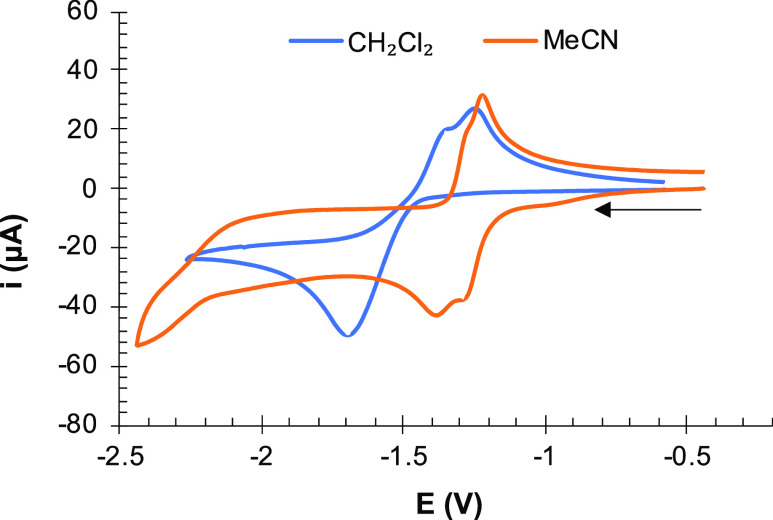
Cyclic voltammograms of compound **6** (10^–3^ M) in CH_2_Cl_2_ (blue line) and MeCN (orange line; intensity multiplication factor = 4) solutions containing 10^–1^ M [NBu_4_]PF_6_ as supporting electrolyte at 25 °C. Counter-electrode: Pt; working electrode: glassy carbon; potential given in V vs Fc^+^/Fc; scan rate: 100 mV/s.

Cyclic voltammetry (CV) was employed to study the electrochemical behavior of complexes **6**, **10a**–**10c**, **11**, and **12**. CVs were recorded in the cathodic direction at a scan rate of 100 mV s^–1^, and the data (versus Fc^+^/Fc) are collected in Table 1 and Figures 4, 6, 7, and 11 and Figures S1 and S2. Platinum compounds **10a**–**10c** and **11** decomposed in CH_3_CN solution, and their electrochemistry was studied in CH_2_Cl_2._

**Table 1:** Electrochemical Data of Compounds 6, 10a-c, 11, 12, 13^a^

		reduction			
entry	compound	*E*_pc_^1^ (*E*_1/2_^1^)	*E*_pc_^2^ (*E*_1/2_^2^)	*E*_pc_^3^ (*E*_1/2_^3^)	*E*_pc_^4^ (*E*_1/2_^4^)
1	**6**^b^			–1.70	
	**6**^c^			–1.28	–1.38
2	**10a**^b^	–0.91 (−0.88)	–1.40 (−1.38)	–1.75	–1.92
3	**10b**^b^	–0.95	–1.42	–1.82	–1.90
4	**10c**^b^	–0.80	–1.47	–1.77	
5	**11**^b^	–1.18		–1.92	
6	**12**^c^	–1.17 (−1.12)	–1.37	–1.89 (−1.85)	–2.10 (−2.07)
7	**13**^b^	–1.26 (−1.22)	–1.72		

a Potential given in V vs Fc^+^/Fc.

b CV recorded in CH_2_Cl_2_.

c CV recorded in MeCN.

The CV of the tetranuclear [Fe_2_S_2_] complex **6** in CH_2_Cl_2_ exhibited a broad reduction wave at *E*_pc_ = −1.70 V, but two oxidation processes were observed in the anodic back scan (*E*_pa_^*1*^ = −1.25 V, *E*_pa_^2^ = −1.33 V) (Figure 4). This observation suggests that two reduction events were occurring at an almost identical potential. In fact, two reversible processes were observed at −1.28 and −1.38 V when the CV was registered in CH_3_CN (Δ*E* = 100 mV) (Figure 4).

Since complex **6** contains three redox-active units, i.e., the [(μ-bdt)][Fe_2_(CO)_5_CN] fragments and the 2,2′-bipyridine spacer, and in order to obtain information about the reduction process, computational DFT studies (SMD(CH_2_Cl_2_)-B3LYP-D3/def2-SVP) were performed in this system.^47^ The study of the frontier orbitals confirmed that the LUMO of **6** connects the two [Fe_2_S_2_] fragments through the bis-isocyanide–bipyridine linker (Figure S42).

**Figure 5:**
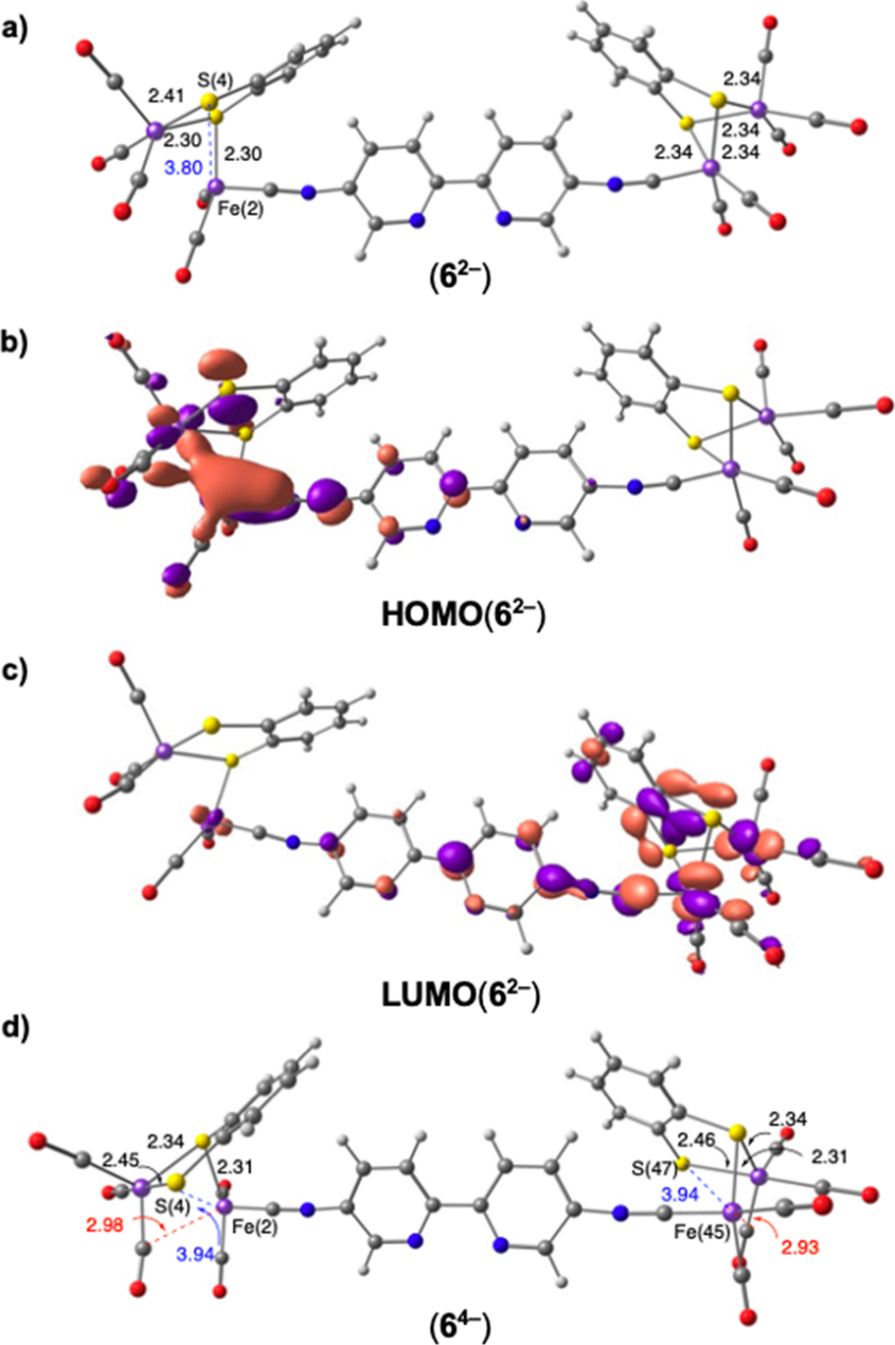
Consecutive two-electron reductions of **6** showing the structural changes in the [Fe_2_S_2_] complexes: (a) dianion **6^2–^**; (b) and (c) HOMO and LUMO orbitals of **6**^**2**–^; (d) tetraanion **6^4–^** (computed at the SMD(CH_2_Cl_2_)-B3LYP-D3/def2-SVP level). Distances in Å. Isosurface value, 0.04.

The computed successive electron uptakes (Figure 5) shows that the reduction of **6** involves the [Fe_2_S_2_] moieties in a sequential manner, as evidenced by the structural changes observed in these fragments along the process. The incorporation of the first two electrons particularly affects one of the [Fe_2_S_2_] centers^23^ and provokes the cleavage of the Fe(2)-S(4) bond [Fe(2)-S(4) distance changes from 2.34 Å in **6^0^** to 3.80 Å in **6^2–^**] (Figure 5a). Accordingly, the orbital HOMO of **6^2–^** is located on the reduced [Fe_2_S_2_] fragment, whereas the LUMO is placed on the unaltered [Fe_2_S_2_] moiety (Figure 5b,c). The uptake of two more electrons (**6^2–^/6^4–^**) occurs on this unaltered [Fe_2_S_2_] unit, causing the breakage of the Fe(45)-S(47) bond (the distance changes from 2.34 Å in **6^2–^** to 3.94 Å in **6^4–^**) (Figure 5d).

The existence of two very close reduction events in the CV of **6** (Δ*E* = 100 mV in CH_3_CN, a single wave in CH_2_Cl_2_) (Figure 4) is compatible with the sequential process of the computational study but indicates that the electronic communication between the iron centers of **6** is very weak. Overall, the sequential reduction process can be summarized in eqs 1 and 2.
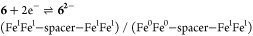
1
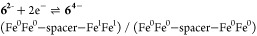
2

The structural changes observed in Figure 5 are analogous to those reported in the computational studies on the reduction of the related [(μ-bdt)][Fe(CO)_3_]_2_ complex **9**,^24−26^ although in this case, the breakage of the Fe–S bonds during the reduction was accompanied by the formation of a bridging CO. Although our calculations did not show bridging CO bonds in the broken [Fe_2_S_2_] fragments, there is a clear approaching of one of the CO ligands to both the Fe(2) and Fe(45) atoms in tetraanion **6**^**4**–^ (final bond distances: 2.98 and 2.93 Å, respectively) (Figure 5). The remarkable structural changes observed in the [Fe_2_S_2_] moieties along the reduction process will be used in this article as probe of reduction processes centered on the [Fe_2_S_2_] fragments when studying the more complex systems **10a**–**10c**, **11**, and **12**.

**Figure 6:**
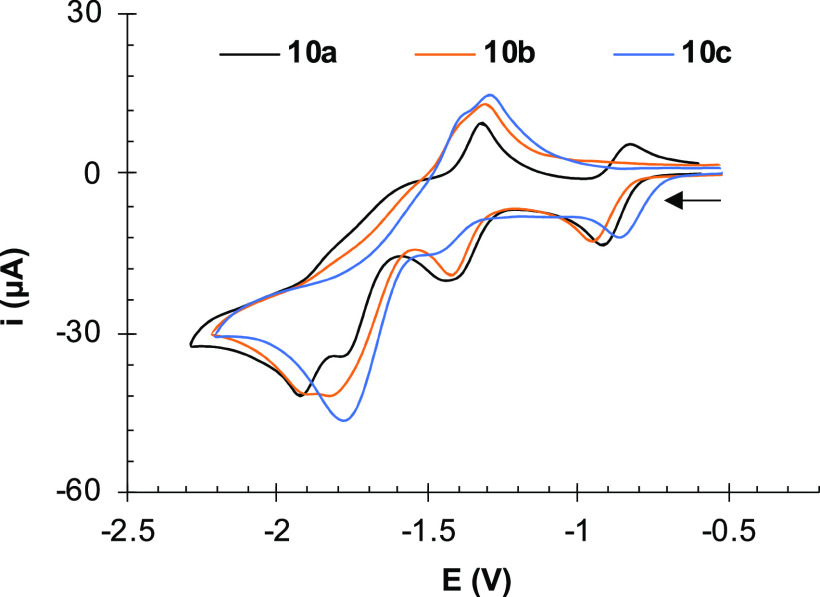
Cyclic voltammograms of compounds **10a**–**10c** (10^–3^ M) in CH_2_Cl_2_ solutions containing 10^–1^ M [NBu_4_]PF_6_ as supporting electrolyte at 25 °C. Counter-electrode: Pt; working electrode: glassy carbon; potential given in V vs Fc^+^/Fc; scan rate: 100 mV/s.

**Figure 7:**
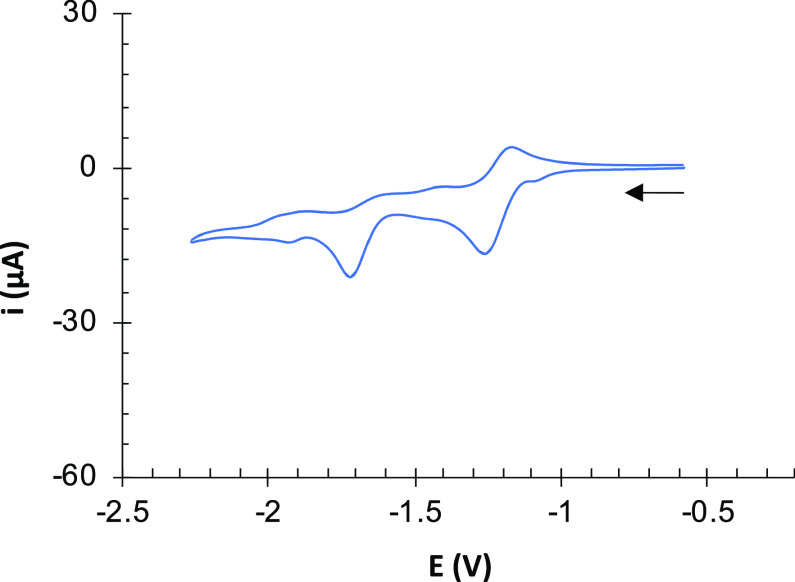
Cyclic voltammogram of [Pt(dppe)(bpy)][PF_6_] (**13**) (10^–3^ M, CH_2_Cl_2_, 10^–1^ M [NBu_4_]PF_6_ as supporting electrolyte at 25 °C). Counter-electrode: Pt; working electrode: glassy carbon; potential given in V vs Ag/AgCl; scan rate: 100 mV/s.

The effect of the coordination to different Pt(II) complexes through the bipyridine ligand in electronic communication between the two [Fe_2_S_2_] centers of **6** was addressed next. The CVs of **10a**–**10c** contain up to four reduction processes in the −0.9 to −1.9 V range (data in Table 1 and Figure 6). Based on DFT studies (see Figures S52–S57), the first two waves at *E*_pc_^1^ ≈ −0.9 V and *E*_pc_^2^ ≈ −1.40 V can be assigned to the successive one-electron reductions of the bipyridine moiety (bipy^0/1–^ and bipy^1–/2–^). This is in agreement with the behavior reported for other Pt(II)-bipyridine complexes.^27^ To support this asseveration, model compound [Pt(dppe)(bpy)][PF_6_] (**13**) was prepared, and its CV was recorded (Table 1 and Figure 7). It shows two reduction events at *E*_pc_^1^ = −1.26 V (reversible) and *E*_pc_^2^*=* −1.72 V (irreversible). These values are anodically shifted by 30 mV compared to those of **10a**–**10c**, likely as a result of the electron withdrawing effect caused by the [Fe_2_S_2_] centers as substituents in the bipyridine skeleton.^10,11^

After the reduction of the bipyridine moiety (that would lead to species **10a–10c^2–^**), the successive electron uptakes should necessarily involve the [Fe_2_S_2_] moieties. However, the CVs of complexes **10a**–**10c** in Figure 6 show differences depending on the structure of the Pt(II) complex. Thus, two waves are clearly observed in the CV of **10a** (*E*_pc_^3^ = −1.75 V; *E*_pc_^4^ = −1.92 V), but they approach in the CV of **10b** (*E*_pc_^3^ = −1.82 V; *E*_pc_^4^ = −1.90 V) and turned into a single broad wave for **10c** (*E*_pc_^3^ = −1.77 V).

**Figure 8:**
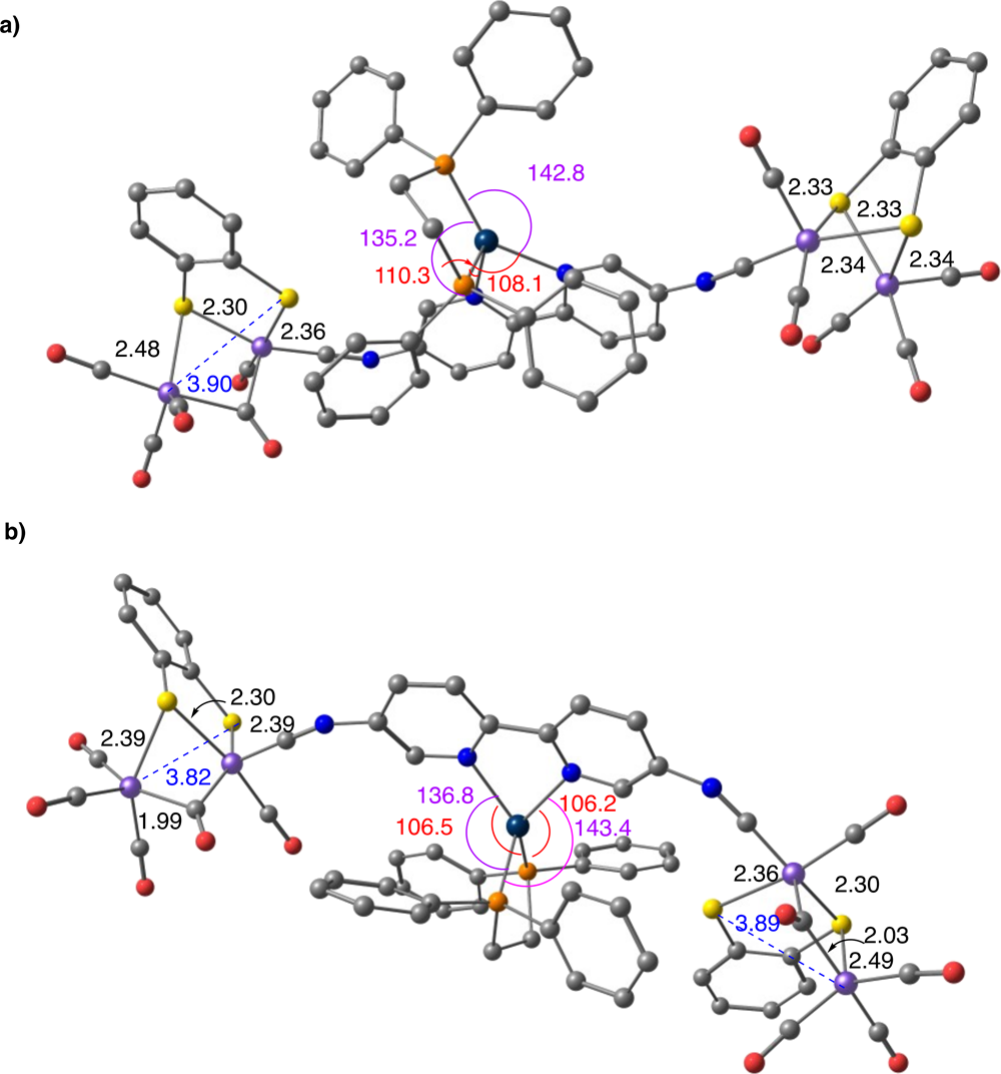
Changes of the structure of the Pt(II) complex during the reduction of the [FeS_2_] moieties in species **10a^2–^**: (a) **10a^4–^**; (b) **10a^6–^**. Computed at the SMD(CH_2_Cl_2_)-B3LYP-D3/def2-SVP level. Distances in Å. H atoms omitted for clarity.

The DFT calculations confirmed that the successive reductions of the [Fe_2_S_2_] centers of the species **10a–10c^2–^** were accompanied by structural changes in the iron–sulfur moieties, as described above for complex **6**. Interestingly, the cleavage of the Fe–S bonds and the reorganization of terminal CO ligands to a bridging position were not the only changes observed as the geometry of the Pt(II) complexes was also deeply affected during the reductions. For complex **10a**,^28^ bearing a dppe ligand, the successive two electron uptakes of the reduced species **10a^2–^** (**10a^2–^**/**10a^4–^** and **10a^4–^**/**10a^6–^**) causes the progressive deviation of the Pt(II) center from the initial square-planar structure to finally reach a distorted-tetrahedral geometry in **10a^6–^**, formally a Pt(0) complex (Figure 8).^29−31^ There, one of the P atoms (P1) is almost perpendicular to the equatorial plane that contains the three other donor atoms (P^1^–Pt–N angles: 106.5 and 106.2°; P^2^–Pt–N angles: 136.8 and 143.4°). The structural changes in the Pt center are compatible with an increase in electron density on the metal along the reduction process, which is in consonance with the existence of electronic communication between the [Fe_2_S_2_] units. Therefore, the two waves at *E*_pc_^3^ = −1.75 V and *E*_pc_^4^ = −1.92 V in the CV of **10a** could be respectively assigned to the calculated **10a^2–^**/**10a^4–^** and **10a^4–^**/**10a^6–^** couples, which suggests that the reduction of the first [Fe_2_S_2_] unit increases the electronic density of the other through the Pt(dppe)-bipyridine linker. The difference between the *E*_pc_^3^ and *E*_pc_^4^ (Δ*E* = 170 mV) indicates a weak electronic interaction between the [Fe_2_S_2_] units in **10a**. However, compared to complex **6** (a single wave in CH_2_Cl_2_), the effect of the incorporation of the Pt(dppe) complex on the electronic communication is highly remarkable.

The influence of the structure of the bisphosphine ligand was clearly manifested during the reduction of the [Fe_2_S_2_] moieties in the species **10b**^**2**–^ and **10c**^**2**–^. A comparative computational study (Figures S54 and S56) reveals that the Pt-center of complex **10b**, containing the flexible dppp ligand, was only slightly distorted during the reductions of the [Fe_2_S_2_] units (**10b^2–^**/**10b^4–^** and **10b^4–^**/**10b^6–^**) (*cis*-P–Pt–N angles: 94.0 and 97.1°), whereas complex **10c**, bearing a dppf ligand, suffered the elongation of one of the N–Pt bonds during the processes, allowing the distortion of the initially coplanar bipyridine ring. The effect of these changes in the electronic communication between the [Fe_2_S_2_] units is significant. The CV of complex **10b** still shows two reduction waves (*E*_pc_^3^ = −1.82 V and *E*_pc_^4^ = −1.90 V) assignable to the **10b^2–^**/**10b^4–^** and **10b^4–^**/**10b^6–^** events, but the difference in potential values (Δ*E* = 80 mV) indicate that the electronic interaction is much weaker than in **10a**. In turn, for complex **10c**, there is only a broad reduction wave assignable to the **10c^2–^**/**10c^4–^** and **10c^4–^**/**10c^6–^** events (*E*_pc_^3^ = −1.77 V), revealing the lack of electronic communication in this complex. In a way, the decoordination of the Pt(dppf) during the reduction of the [Fe_2_S_2_] centers in **10c** makes it comparable to complex **6**, having a free bipyridine linker.

Overall, the sequential reductions of platinum complexes **10** can be summarized in eqs 3 to 6.
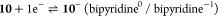
3
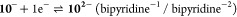
4
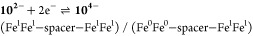
5
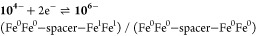
6

The CV of compound **11**, bearing a Ni(II)-dppe fragment, shows two irreversible reduction waves at *E*_pc_ = −1.18 and −1.92 V (Figure S1). Despite the fact that this complex is stable in solution and could be fully characterized, we were unable to obtain reproducible successive CVs, suggesting that **11** and their reduced species are not stable under the experimental electrochemical conditions. Therefore, compound **11** was excluded from the rest of the study.

**Figure 9:**
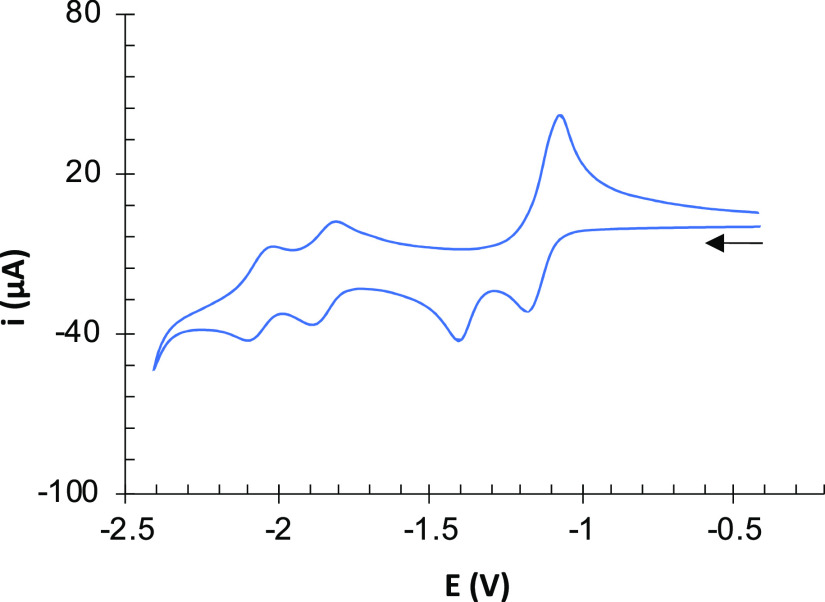
Cyclic voltammogram of compound **12** (10^–3^ M in CH_3_CN, containing 10^–1^ M [NBu_4_]PF_6_ as supporting electrolyte at 25 °C). Counter-electrode: Pt; working electrode: glassy carbon; potential given in V vs Fc^+^/Fc; scan rate: 100 mV/s. The CV of **12** was also recorded in CH_2_Cl_2_. However, the shorter solvent window associated with this solvent, prevented the observation of all the electrochemical processes. See Figure S2 for more details.

**Figure 10:**
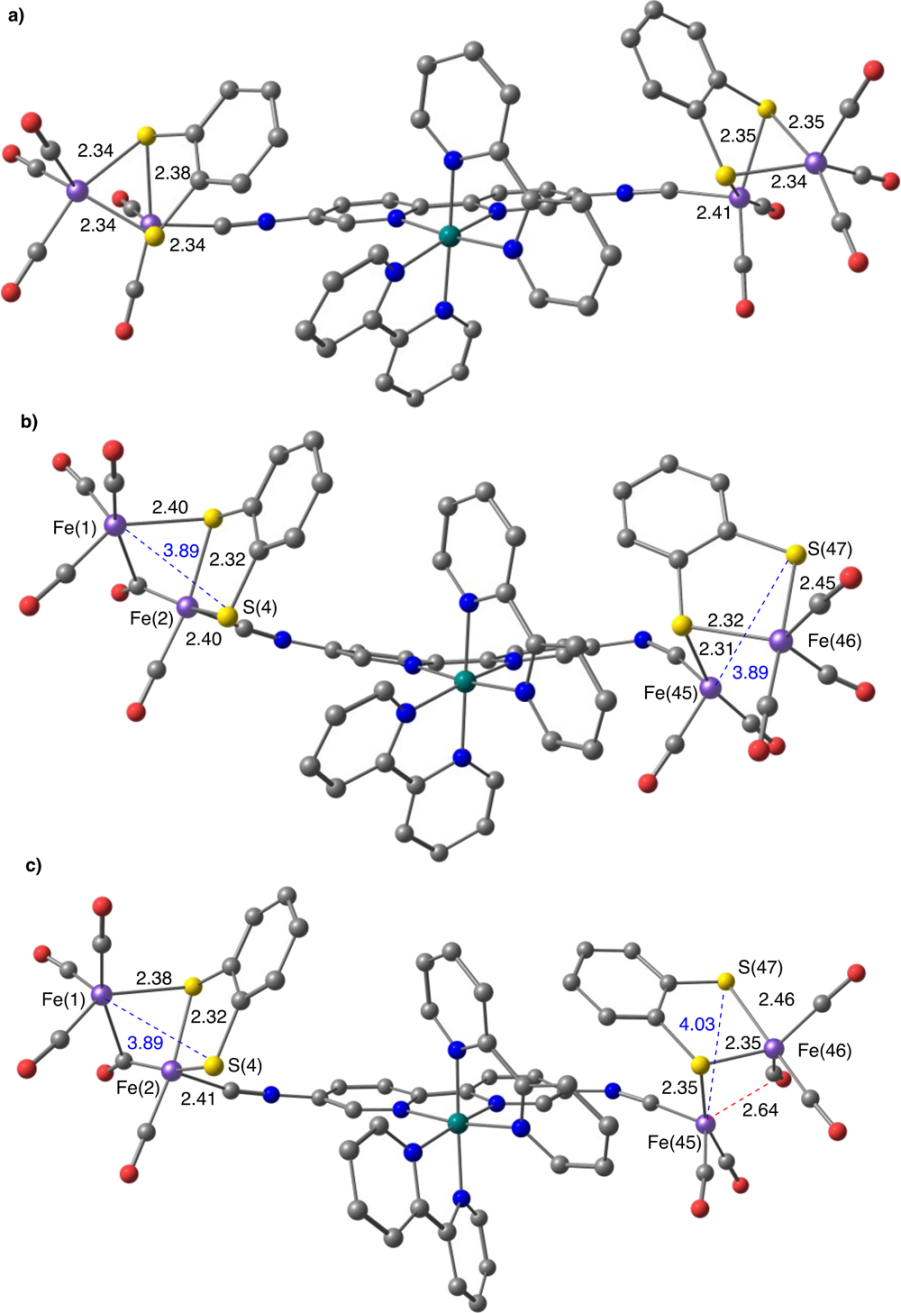
Computed structures of complex **12** (SMD(CH_2_Cl_2_)-B3LYP-D3/def2-SVP level) showing the changes in the structure of the [Fe_2_S_2_] complexes upon reduction. (a) **12^4–^**; (b) **12^6–^**; (c) **12^8–^**. Distances in Å. H atoms omitted for clarity.

The electrochemical behavior of the octahedral Ru(II) species (**12**) was studied next. The CV of **12** in CH_3_CN shows four reduction events (Table 1 and Figure 9). The waves at *E*_pc_^1^ = −1.12 V (reversible) and *E*_pc_^2^ = −1.37 V (irreversible) can be assigned to the reductions of the bipyridine moieties, in agreement with the electrochemical behavior reported for other Ru-bipyridine complexes.^32−35^ According to the DFT calculations, the first sequential two-electron uptake involves the bipy^0/1–^ and bipy^1–/2–^ of the bis-isocyanide–bipyridine linker, while the next two-electron reduction can be assigned to the bipy^0/1–^ and bipy^1–/2–^ of one of the bipyridine–Ru ligands (see Figure 10a and Figures S58–S61).

After the reduction of the bipyridine moieties (that would lead to species **12^4–^**), the successive electron uptakes should necessarily involve the [Fe_2_S_2_] moieties (Figures S61 and S62). The CV of **12** also shows two reversible reduction waves at *E*_pc_^3^ = −1.85 and *E*_pc_^4^ = −2.07 V, compatible with the expected sequential two-electron reductions of the [Fe_2_S_2_] units. Accordingly to the computation, the first of them could be consistent with the **12^4–^**/**12^6–^** event, a process that causes a noticeable structural change in both [Fe_2_S_2_] fragments, with the characteristic Fe–S bond cleavage associated to the Fe^I^Fe^I^/Fe^I^Fe^0^ reduction [(Fe(1)–S(4) and (Fe(45)–S(47) distances change from 2.34 Å in **12^4–^** to 3.89 Å in **12^6–^**]. In this case the reduction is accompanied by the formation of a bridging CO ligand between Fe(1) and Fe(2) (Figure 10b). The DFT calculations confirmed that the following reduction, **12^6–^**/**12^8–^**, also involves the [Fe_2_S_2_] moieties, although now the additional structural changes in both units are subtle (Figure 10c).

It is well known that the reduction of [(μ-bdt)][Fe(CO)_3_]_2_ and structurally related complexes is an overall two-electron process, consisting on two successive monoelectronic events, the second one occurring at a less negative potential than the first one. As a result, only a single reduction wave is usually observed in their CVs.^8,10,11,24,36−38^ The electrochemical study of **12** (Figure 9) reveals a different behavior as the two successive reduction events of the [Fe_2_S_2_] units appear at very different potential values (Δ*E* = 210 mV). This result is undoubtedly related to the electronic connection between the [Fe_2_S_2_] fragments through the highly-reduced Ru-bipyridine spacer. In support, the DFT analysis of the anion-radical **12^7–^** formed by monoelectronic reduction of **12^6–^** reveals the spin density shared between the connected Fe atoms (0.06 e^**–**^ and 0.11 e**^–^,** respectively) and the Ru-bipyridine spacer (Figure S63).

### Electrocatalytic Hydrogen Evolution Reaction from Acetic Acid

**Figure 11:**
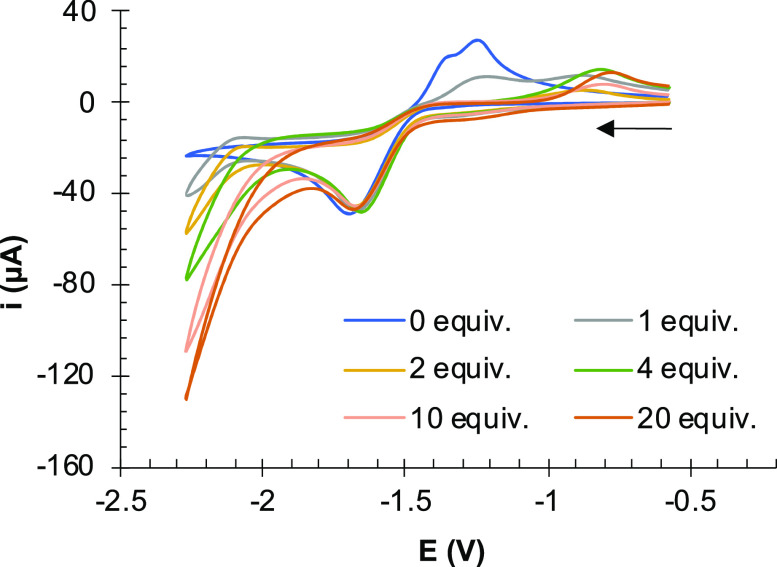
Electrochemical response of a CH_2_Cl_2_ solution of **6** (10 ^–3^ M) in the presence of increasing amounts of acetic acid (0–20 equiv). Cyclic voltammograms registered at 25 °C. Supporting electrolyte: [NBu_4_]PF_6_ (10^–1^ M). Counter-electrode: Pt; working electrode: glassy carbon; potential given in V vs Fc^+^/Fc; scan rate: 100 mV/s.

The electrocatalytic behavior of compounds **6**, **10a–10c** and **12** in the reduction of protons from acetic acid was explored.^39^ Complex **6** showed the expected electrocatalytic behavior of a [(μ-bdt)][Fe(CO)_3_]_2_ derivative (Figure 11).^11,24,37,38^ The intensity of the first reduction event (−1.70 V) did not change upon addition of increasing amounts of acetic acid to a solution of complex **6** in CH_3_CN. However, this process becomes irreversible and the current intensity at −2.30 V increases with the acid concentration. The observed behavior is in agreement with an electrocatalytic process occurring at this latter potential.

The electrochemical behavior of the Pt complexes **10a**–**10c** and the Ru complex **12** in the presence of acetic acid was also evaluated (Figure S3). As in **6**, upon acid addition, the CVs of Pt complexes, **10a–10c**, show the increase of current intensity at −2.30 V, but a current enhancement was also observed at less negative potentials, particularly in the waves assignable to the reduction of the [FeS_2_] fragments of complexes **10a** and **10b**. Both complexes have some degree of electronic interaction between the iron–sulfur centers through the Pt(II) spacer.

For complex **12** (Figure S3), the current intensity of the wave at −2.07 V increases upon the increase of AcOH concentration. The catalytic reduction of acetic acid occurs now at less negative potential than with complex **6** or Pt complexes **10a–10c** (−2.30 V).

## Conclusions

Herein, we report a tetranuclear [Fe_2_S_2_] complex (**6**) bearing a flexible conjugate linker that is able to act as a ligand to coordinate to Pt(II), Ni(II), and Ru(II) metal complexes. The incorporation of the metal complex in the linker is decisive for electronic communication between the redox-active [Fe_2_S_2_] units, likely because the metal coordination forces the bipyridine to be coplanar. Thus, diphosphine platinum compounds **10** enable the electronic interaction between the [FeFe] centers, but the structure of the phosphine ligand plays a crucial role to facilitate or to hinder the process. A weak electronic communication (Δ*E* = 170 mV) was observed for complex **10a**, bearing a dppe ligand, whereas the interaction is much weaker in the Pt-dppp derivative **10b** (Δ*E* = 80 mV) and virtually negligible in the Pt-dppf complex **10c**. Alternatively, the electronic communication is facilitated by incorporation of a Ru-bis(bipyridine) complex, **12** (Δ*E* = 210 mV), although the reduction of the [FeFe] centers occur at more negative potentials. The results shown here offer a new approach to the design of polynuclear iron–sulfur complexes with flexible conjugated bridges, in which the electronic interaction between the redox-active centers can be modulated by a transition metal.

## Experimental Section

### General Methods

Unless stated otherwise, all the reactions were carried out under an Ar atmosphere using anhydrous solvents. The reaction work-ups were performed in air. [2,2′-Bipyridine]-5,5′-diamine, complexes **9** and **13**, [Ni(dppe)Cl_2_], [Pt(dppe)Cl_2_], and [Ru(bipy)_2_Cl_2_]·2H_2_O were prepared according to reported protocols.^17,22,40,41^ Commercially available reagents (Me_3_NO·2H_2_O, POCl_3_, NaBAr^F^_4_, [Pt(dppf)Cl_2_], and [Pt(dppp)Cl_2_]) were used as received without further purification. ^1^H, ^13^C{^1^H}, ^31^P{^1^H}, and ^19^F{^1^H} NMR spectra were recorded at ambient temperature in CDCl_3_ or DMSO-*d*_6_ on Bruker 500 or 300 MHz spectrometers. Chemical shifts are expressed in parts per million and referenced to residual solvent peaks (^1^H and ^13^C{^1^H}) or to an external reference (85% H_3_PO_4_ aqueous solution for ^31^P{^1^H} and C_6_H_5_CF_3_ for ^19^F{^1^H}). FTIR spectra (ATR) were recorded as solids or films (by slowly evaporating CHCl_3_ solutions of the compounds) on a Bruker Alpha spectrometer. ESI-HRMS was performed on an Agilent 6500 accurate mass spectrometer with a Q-TOF analyzer. Elemental analyses were carried out on an elemental microanalyzer LECO CHNS-932. Cyclic voltammograms were recorded using a Metrohm Autolab Potentiostat model PGSTAT302N with a glassy carbon working electrode, an Ag/AgCl 3 M as reference, and a Pt wire counter electrode. All the measurements were performed under Ar at room temperature from CH_2_Cl_2_ or CH_3_CN solutions containing 0.1 M [NBu_4_]PF_6_ as supporting electrolyte with analyte concentrations of 1 mM (scan rate 0.1 V/s).

#### Computational Details

All calculations were performed at the DFT level using the B3LYP functional^42^ as implemented in Gaussian09^43^ supplemented with Grimme’s dispersion correction D3^44^ and the def2-SVP basis set.^45^ All minima were verified to have no negative frequencies. The geometries were fully optimized in vacuo and in CH_2_Cl_2_ or CH_3_CN using the continuum SMD model.^46^

### Complex **6**

In a 500 mL round-bottom flask, complex **9** (1.45 g, 3.45 mmol, 2 equiv) was dissolved in a CH_2_Cl_2_/CH_3_CN (2:1) mixture (105 mL). To this solution, a suspension of Me_3_NO·2H_2_O (384 mg, 3.45 mmol, 2 equiv) in CH_3_CN (105 mL) was added. After 5 min of stirring at room temperature, a solution of **8** (356 mg, 1.73 mmol, 1 equiv) in CH_2_Cl_2_ (35 mL) was added to the suspension. The reaction mixture was stirred at room temperature for 1.5 h. After this time, the solvent was removed under reduced pressure, and the reaction crude was purified by flash chromatography using a mixture of *n*-hexane/EtOAc (97:3) as eluent. Complex **6** was obtained as a red solid in 40% yield (831 mg). ^1^H NMR (500 MHz, CDCl_3_): δ 8.54 (br s, 2H, CH_py_), 8.45 (d, *J* = 8.5 Hz, 2H, CH_py_), 7.65 (d, *J* = 8.5 Hz, 2H, CH_py_), 7.13 (dd, *J* = 5.5, 3.2 Hz, 4H, CH_SAr_), 6.61 (dd, *J* = 5.5, 3.2 Hz, 4H, CH_SAr_) ppm. ^13^C{^1^H} NMR (126 MHz, CDCl_3_): δ 210.6 (CO), 209.0 (CO), 175.7 (CN), 153.6 (C_py_), 148.8 (C_SAr_), 146.7 (CH_py_), 134.0 (CH_py_), 127.8 (CH_SAr_), 126.5 (C_py_), 126.4 (CH_SAr_), 121.9 (CH_py_) ppm. IR (film): ν_C≡N_ 2115 (s); ν_C≡O_ 2077 (m), 2038 (vs) and 1979 (vs) cm^–1^. HRMS-ESI: *m/z* calcd. for C_34_H_14_Fe_4_N_4_O_10_S_4_ [M + H]^+^: 990.70687; found [M + H]^+^: 990.70488.

### General Procedure for Synthesis of Complexes **10a**–**10c** and **11**

In a round-bottom flask, complex **6** (100 mg, 0.100 mmol, 1.01 equiv) and NaBAr^F^_4_ (198 mg, 0.218 mmol, 2.2 equiv) were suspended in CH_2_Cl_2_ (15 mL). To this mixture, a solution of the corresponding [M(P^P)Cl_2_] (0.099 mmol, 1 equiv) in CH_2_Cl_2_ (10 mL) was added via a cannula The reaction mixture was stirred at room temperature for 2.5–24 h. After this time, the obtained suspension was filtered through Celite and the solvent was removed under reduced pressure. The residue was washed with a mixture of *n*-pentane/CH_2_Cl_2_ (9:1) (6 × 5 mL), affording the pure product as a solid.

#### Compound **10a**

Following the general procedure (with 66 mg of [Pt(dppe)Cl_2_]), **10a** was isolated after 24 h as a dark purple solid in quantitative yield (330 mg). ^1^H NMR (500 MHz, CDCl_3_): δ 7.75 (m, 12H + 2H, CH_PPh_ + CH_py_), 7.66 (br s, 16H, CH_B(ArF)4_), 7.61–7.55 (m, 8H + 2H, CH_PPh_ + CH_py_), 7.42 (s, 8H, CH_B(ArF)4_), 7.24 (d, *J* = 9.9 Hz, 2H, CH_py_), 7.11 (dd, *J* = 5.5, 3.2 Hz, 4H, CH_SAr_), 6.65 (dd, *J* = 5.5, 3.2 Hz, 4H, CH_SAr_), 2.47 (m, 4H, PCH_2_) ppm. ^13^C{^1^H} NMR (126 MHz, CDCl_3_): δ 208.2 (CO), 189.2 (CN), 161.8 (q, *J*_(C-F)_*=* 49.9 Hz, C_B(ArF)4_), 151.7 (C_py_), 148.5 (CH_py_), 147.3 (C_SAr_), 138.4 (CH_py_), 136.1 (CH_PPh_), 134.8 (CH_B(ArF)4_), 133.5 (CH_PPh_), 131.6 (m, CH_PPh_), 131.5 (m, C_PPh_), 130.1 (C_py_), 129.1 (q, *J*_(C–F)_ = 33.1 Hz, C_B(ArF)4_), 128.0 (CH_SAr_), 127.2 (CH_SAr_), 124.9 (CH_py_), 124.5 (q, *J*_(C–F)_ = 272.7 Hz, C_B(ArF)4_), 117.7 (CH_B(ArF)4_), 29.2 (d, *J*_(C–P)_ = 50.4 Hz, PCH_2_) ppm. ^31^P{^1^H} NMR (202 MHz, CDCl_3_): δ 43.77 (*J*_(P–Pt)_ = 3346.8 Hz) ppm.^19^F{^1^H} NMR (471 MHz, CDCl_3_): δ −62.72 ppm. IR (film): ν_C≡N_ 2087 (m); ν_C≡O_ 2041 (s) and 1993 (vs); ν_C–F_ 1354 (s) and 1276 (vs); ν_C–B_ 1123 (vs) cm^–1^. Anal. calcd (%) for C_124_H_62_B_2_F_48_Fe_4_N_4_O_10_P_2_PtS_4_: C 44.99; H, 1.89; N, 1.69; S, 3.87. Found: C, 44.86; H, 1.90; N, 1.72; S, 3.73.

#### Compound **10b**

Following the general procedure (with 68 mg of [Pt(dppp)Cl_2_]), **10b** was isolated after 24 h as a dark purple solid in quantitative yield (331 mg). ^1^H NMR (500 MHz, CDCl_3_): δ 7.79–7.73 (m, 12H, CH_PPh_), 7.67 (br s, 16H + 2H, CH_B(ArF)4_ + CH_py_), 7.60 (br s, 8H, CH_PPh_), 7.51 (d, *J* = 8.4 Hz, 2H, CH_py_), 7.44 (s, 8H, CH_B(ArF)4_), 7.15–7.11 (m, 4H + 2H, CH_SAr_ + CH_py_), 6.66 (br s, 4H, CH_SAr_), 2.49 (br s, 2H, PCH_2_C*H*_2_), 2.12 (m, 4H, PC*H_2_*CH_2_) ppm. ^13^C{^1^H} NMR (126 MHz, CDCl_3_): δ 208.2 (CO), 189.0 (CN), 161.8 (q, *J*_(C-F)_*=* 49.9 Hz, C_B(ArF)4_), 151.6 (C_py_), 148.8 (CH_py_), 147.3 (C_SAr_), 137.9 (CH_py_), 135.8 (CH_PPh_), 134.8 (CH_B(ArF)4_), 132.9 (CH_PPh_), 131.6 (m, CH_PPh_ + C_PPh_), 129.6 (C_py_), 129.2 (q, *J*_(C–F)_ = 32.6 Hz, C_B(ArF)4_), 128.0 (CH_SAr_), 127.2 (CH_SAr_), 124.7 (CH_py_), 124.6 (q, *J*_(C–F)_ = 272.7 Hz, C_B(ArF)4_), 117.7 (CH_B(ArF)4_), 21.4 (d, *J*_(C–P)_ = 31.8 Hz, P*C*H_2_CH_2_), 16.4 (PCH_2_*C*H_2_) ppm. ^31^P{^1^H} NMR (202 MHz, CDCl_3_): δ -2.62 (*J*_(P–Pt)_*=* 3230.6 Hz) ppm. ^19^F{^1^H} NMR (471 MHz, CDCl_3_): δ −62.69 ppm. IR (film): ν_C≡N_ 2088 (m); ν_C≡O_ 2043 (s) and 1998 (vs); ν_C −F_ 1355 (s) and 1277 (vs); ν_C–B_ 1125 (vs) cm^–1^. Anal. calcd (%) for C_125_H_64_B_2_F_48_Fe_4_N_4_O_10_P_2_PtS_4_: C, 45.17; H, 1.94; N, 1.69; S, 3.86. Found: C, 44.79; H, 2.04; N, 1.65; S, 3.71.

#### Compound **10c**

Following the general procedure (with 82 mg of [Pt(dppf)Cl_2_]), **10c** was isolated after 24 h as a dark purple solid in quantitative yield (345 mg). ^1^H NMR (500 MHz, CDCl_3_): δ 7.79–7.72 (m, 8H, CH_PPh_), 7.69 (br s, 16H + 2H, CH_B(ArF)4_ + CH_py_), 7.64 (t, *J* = 7.7 Hz, 4H, CH_PPh_), 7.50 (dd, *J* = 8.5, 2.1 Hz, 2H, CH_py_), 7.47–7.43 (m, 8H + 8H, CH_B(ArF)4_ + CH_Ph_), 7.26 (d, *J* = 8.5 Hz, 2H, CH_py_), 7.11 (dd, *J* = 5.5, 3.2 Hz, 4H, CH_SAr_), 6.64 (dd, *J* = 5.5, 3.2 Hz, 4H, CH_SAr_), 4.61 (s, 4H, CH_Cp_), 4.34 (s, 4H, CH_Cp_) ppm. ^13^C{^1^H} NMR (126 MHz, CDCl_3_): δ 208.4 (CO), 188.8 (CN), 161.8 (q, *J*_(C–F)_ = 49.9 Hz, C_B(ArF)4_), 151.9 (C_py_), 149.2 (CH_py_), 147.4 (C_SAr_), 138.2 (CH_py_), 135.1 (CH_PPh_), 134.9 (CH_B(ArF)4_), 133.6 (CH_PPh_), 130.7 (m, CH_PPh_ + C_PPh_), 129.3 (C_py_), 129.1 (q, *J*_*(*C–F)_ = 31.9 Hz, C_B(ArF)4_), 128.0 (CH_SAr_), 127.1 (CH_SAr_), 124.6 (q, *J*_(C–F)_ = 272.7 Hz, C_B(ArF)4_), 124.5 (CH_py_), 117.7 (CH_B(ArF)4_), 77.1 (m, CH_Cp_ + C_Cp_) ppm. ^31^P{^1^H} NMR (202 MHz, CDCl_3_): δ 12.87 (*J*_(P–Pt)_ = 3349.6 Hz) ppm. ^19^F{^1^H} NMR (471 MHz, CDCl_3_): δ −62.65 ppm. IR (film): ν_C≡N_ 2090 (m); ν_C≡O_ 2043 (s) and 1999 (s); ν_C–F_ 1355 (s) and 1277 (vs); ν_C–B_ 1126 (vs) cm^–1^. Anal. calcd (%) for C_132_H_66_B_2_F_48_Fe_5_N_4_O_10_P_2_PtS_4_: C, 45.74; H, 1.92; N, 1.62; S, 3.70. Found: C, 45.36; H, 2.03; N, 1.62; S, 3.43.

#### Compound **11**

Following the general procedure (with 53 mg of [Ni(dppe)Cl_2_]), **11** was isolated after 2.5 h as a dark purple solid in quantitative yield (314 mg). ^1^H NMR (500 MHz, CDCl_3_) δ: 7.91 (br s, 8H, CH_PPh_), 7.77 (t, *J* = 7.7 Hz, 4H, CH_PPh_), 7.68 (br s, 16H, CH_B(ArF)4_), 7.60 (t, *J* = 7.7 Hz, 8H, CH_PPh_)7.56 (d, *J* = 2.2 Hz, 2H, CH_py_) 7.46 (dd, *J* = 8.8, 2.2 Hz, 2H, CH_Py_), 7.42 (s, 8H, CH_B(ArF)4_), 7.12 (dd, *J* = 5.5, 3.3 Hz, 4H, CH_SAr_), 7.03 (d, *J* = 8.8 Hz, 2H, CH_py_), 6.65 (dd, *J* = 5.5, 3.2 Hz, 4H, CH_SAr_), 2.30 (m, 4H, PCH_2_) ppm. ^13^C{^1^H} NMR (126 MHz, CDCl_3_): δ 208.3 (CO), 188.3 (CN), 161.8 (q, *J*_C–F_ = 49.7 Hz, C_B(ArF)4_), 151.2 (C_py_), 149.5 (CH_py_), 147.3 (C_SAr_), 138.5 (CH_py_), 136.2 (CH_PPh_), 134.8 (CH_B(ArF)4_), 133.1 (CH_PPh_), 131.8 (m, CH_PPh_ + C_PPh_), 129.2 (q, *J*_C-F_ = 33.6 Hz, C_B(ArF)4_), 129.0 (C_py_), 128.0 (CH_SAr_), 127.17 (CH_SAr_), 124.5 (q, *J*_C–F_ = 272.9 Hz, C_B(ArF)4_), 123.5 (CH_py_), 117.7 (CH_B(ArF)4_), 29.0 (t, *J*_C–P_ = 25.0 Hz, PCH_2_) ppm. ^31^P{^1^H} NMR (202 MHz, CDCl_3_): δ 67.65 ppm. ^19^F{^1^H} NMR (471 MHz, CDCl_3_): δ −62.64 ppm. IR (film): ν_C≡N_ 2088 (m); ν_C≡O_ 2041 (s) and 1994 (vs); ν_C–F_ 1354 (s) and 1276 (vs); ν_C–B_ 1123 (vs) cm^–1^. Anal. calcd (%) for C_124_H_62_B_2_F_48_Fe_4_N_4_NiO_10_P_2_S_4_: C, 46.93; H, 1.97; N, 1.77; S, 4.04. Found: C, 46.66; H, 1.90; N, 1.74; S, 3.83.

#### Compound **12**

In a 50 mL round-bottom flask, *cis*-[Ru(bpy)_2_Cl_2_]·2H_2_O (150 mg, 1 equiv) was dissolved in 18 mL of degassed acetone and AgBF_4_ (114.5 mg, 2 equiv) was added to the mixture. The reaction was stirred for 5 h at room temperature protected from the light. After this time, the suspension was filtered through Celite in order to remove AgCl. Then, 25 mL of CH_2_Cl_2_ and 316 mg (1.1 equiv) of the complex **6** were added to the filtrate. The reaction mixture was stirred for 18 h at room temperature. After this time, the solution was concentrated to 4–5 mL and Et_2_O was added until an orange precipitate was obtained. The solid was collected and purified by column chromatography over Al_2_O_3_ (neutral, activity I) using a mixture of CH_2_Cl_2_/MeOH (95:5) as eluent. Complex **12** was obtained as an orange solid in 86% yield (350 mg). ^1^H NMR (500 MHz, CD_3_CN): δ 8.53 (d, *J* = 8.2 Hz, 4H, CH_bipy_), 8.45 (d, *J* = 8.8 Hz, 2H, CH_py_), 8.13–8.08 (m, 4H, CH_bipy_), 7.90 (d, *J* = 8.8 Hz, 2H, CH_py_), 7.69 (d, *J* = 5.5 Hz, 2H, CH_bipy_), 7.63 (d, *J* = 5.5 Hz, 2H, CH_bipy_), 7.62 (s, 2H, CH_py_), 7.48–7.38 (m, 4H, CH_bipy_), 7.17 (dd, *J* = 5.5, 3.2 Hz, 4H, CH_SAr_), 6.68 (dd, *J* = 5.5, 3.2 Hz, 4H, CH_SAr_) ppm. ^13^C{^1^H} NMR (126 MHz, CD_3_CN): δ 211.3 (CO), 209.9 (CO), 178.1 (CN), 157.8(C), 155.9 (C), 153.1 (CH), 152.6 (CH), 150.9 (CH), 149.0 (C), 139.3 (CH), 135.2 (CH), 129.2 (C), 128.8 (CH), 128.7 (CH), 128.5 (CH), 127.7 (CH), 126.4 (CH), 125.4 (CH), 125.4 (CH) ppm. ^19^F{^1^H} NMR (471 MHz, CD_3_CN): δ −152.14 (^10^BF_4_^–^), −152.20 (^11^BF_4_^–^) ppm. IR (film): ν_C≡N_ 2109 (m); ν_C≡O_ 2040 (s) and 1979 (vs); ν_B–F_ 1052 (vs) cm^–1^. HRMS-ESI: *m/z* calcd. for C_54_H_30_Fe_4_N_8_O_10_RuS_4_ [M]^2+^: 701.86990; found [M]^2+^: 701.87625.

## Supporting Information Available

Synthesis and characterization of compounds **7** and **8**; electrochemical information; computational details; DFT coordinates; NMR and IR spectra of all compounds (PDF)
