# Supplementary material for: Heteropolymetallic [FeFe]-Hydrogenase Mimics: Synthesis and Electrochemical Properties
Source: Inorg Chem. 2023 Feb 13;62(8):3409–19. doi: 10.1021/acs.inorgchem.2c03355 (PMC9976291; doi:10.1021/acs.inorgchem.2c03355)
Supplement: Supplementary file 1 — ic2c03355_si_001.pdf [file ic2c03355_si_001.pdf]

ELECTRONIC SUPPLEMENTARY INFORMATION belonging to the manuscript:

Heteropolymetallic [FeFe]-Hydrogenase Mimics: Synthesis and Electrochemical Properties.

Alejandro Torres,[a,b] Alba Collado, *,†,a,b Mar Gómez-Gallego,[a,b] Carmen Ramírez de Arellano,[b,c] and Miguel A. Sierra*,[a,b]

[a] Departamento de Química Orgánica I, Facultad de Química, Universidad Complutense, 28040-Madrid. Spain. [b] Center for Innovation in Advanced Chemistry (ORFEO-CINQA), Facultad de Química, Universidad Complutense, 28040-Madrid. Spain. [c] Departamento de Química Orgánica, Universidad de Valencia 46100-Valencia. Spain.

Index

S.1. Synthesis of compound 8 .... **S2**
S.2. Electrochemical studies **S3**
S.3. Crystal data **S5**
S.4. NMR spectra **S6**
S.5. FTIR spectra **S20**
S.6. DFT calculations. **S24**
S.7. References **S135**

S.1. Synthesis of compound 8

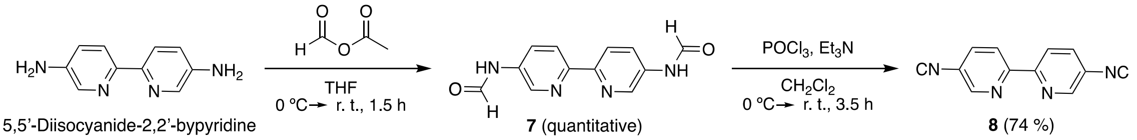

Scheme S1. Synthetic route to prepare **8**.

Compound 7: Acetic formic anhydride was prepared by mixing acetic anhydride (2.25 mL, 23.6 mmol, 4 equiv.) and formic acid (1.00 mL, 26 mmol, 4.4 equiv.) at 55 ºC for 2 h in a pressure tube. This anhydride was added slowly to a solution of 5,5'-diamino-2,2'-bypyridine[1] (1.10 g, 5.9 mmol, 1 equiv.) in THF (28 mL) at 0 ºC in a 100 mL round bottom flask. The reaction mixture was stirred at room temperature for 1.5 h and all the volatiles were evaporated under reduced pressure. A white solid was obtained in quantitative yield (1.44 g). The crude product was used without further purification. Two rotamers were observed by NMR spectroscopy. **^{1}H NMR (300 MHz, DMSO-*d*$_6$) δ:** 10.57 (d, *J* = 1.8 Hz, 2H, NH_{major}), 10.40 (d, *J* = 10.8 Hz, 2H, NH_{minor}), 8.90 (d, *J* = 10.8 Hz, 2H, $CH_{py,minor}$), 8.82 (d, *J* = 2.5 Hz, 2H, $CH_{py,major}$), 8.54 (d, *J* = 2.7 Hz, 2H, CHO_{minor}), 8.39 (d, *J* = 1.8 Hz, 2H, CHO_{major}), 8.29 (d, *J* = 8.7 Hz, 2H, $CH_{py,major}$), 8.26 (m, 2H, $CH_{py,minor}$), 8.16 (dd, *J* = 8.7, 2.5 Hz, 2H, $CH_{py,major}$), 7.75 (dd, *J* = 8.7, 2.7 Hz, 2H, $CH_{py,minor}$) ppm. **^{13}C{^{1}H} NMR (75 MHz, DMSO) δ:** 162.6 (CHO_{minor}), 160.3 (CHO_{major}), 150.4 ($C_{py,minor}$), 150.1 ($C_{py,major}$), 134.0 ($CH_{py,major}$), 138.6 ($CH_{py,minor}$), 135.0 ($C_{py,minor}$), 134.9 ($C_{py,major}$), 127.0 ($CH_{py,major}$), 125.2 ($CH_{py,minor}$), 120.5 ($CH_{py,minor}$), 120.3 ($CH_{py,major}$) ppm. **IR (film):** 3215 (w), 3166 (w), 3089 (w), 3016 (w), 2957 (w), 2896 (w), 1697 (vs), 1581(vs), 1508 (s), 1461 (vs) cm^{-1}. **HRMS-ESI *m/z*:** calcd. for $C_{12}H_{11}N_4O_2$ [M+H]$^+$: 243.08820; found [M+H]$^+$: 243.08836.

Compound 8: In a 100 mL round bottom flask, a suspension of **7** (893 mg, 3.7 mmol, 1 equiv.) in CH_2Cl_2 (25 mL) was cooled down to 0 ºC and NEt_3 (5.14 mL, 10 equiv.) was added. After 5 min, $POCl_3$ (1.38 mL, 14.8 mmol, 4 equiv.) was added dropwise. The reaction mixture was stirred at 0 ºC for 1 h and then for 2.5 h at room temperature. A saturated aqueous solution of $NaHCO_3$ (5 mL) was added very slowly to the flask. The mixture was transferred to a separation funnel. The organic layer was separated and successively washed with a saturated aqueous solution of $NaHCO_3$ (3 x 10 mL), H_2O (2 x 10 mL) and brine (2 x 10 mL), dried over anhydrous Na_2SO_4 and filtered. The solvent was removed *in vacuo*. A yellow solid was obtained in 74 % yield (569 mg). The crude product was used without further purification. **^{1}H NMR (300 MHz, $CDCl_3$) δ:** 8.72 (d, *J* = 2.4 Hz, 2H, CH), 8.52 (d, *J* = 8.5 Hz, 2H, CH), 7.83 (dd, *J* = 8.5, 2.4 Hz, 2H, CH) ppm. **^{13}C{^{1}H} NMR (75 MHz, $CDCl_3$) δ:** 170.4 (CN), 154.5 (C), 147.1 (CH), 134.6 (CH), 124.5 (C), 121.9 (CH) ppm. **IR (film):** $\nu_{C\equiv N}$ 2121 (vs) cm^{-1}. **HRMS-ESI *m/z*:** calcd. for $C_{12}H_7N_4$ [M+H]$^+$: 207.067907; found [M+H]$^+$: 207.06652.

S.2. Electrochemical studies

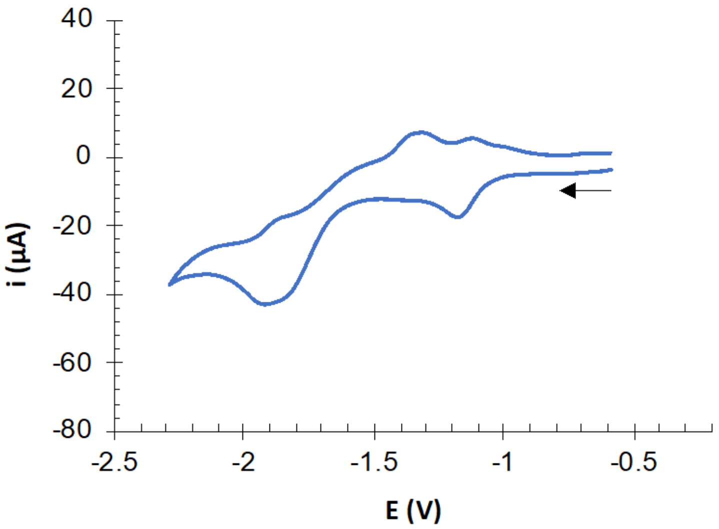

Figure S1. Cyclic voltammogram of a CH_2Cl_2 solution of compound **11** (10^{-3} M) containing 10^{-1} M $[NBu_4]PF_6$ as supporting electrolyte at 25 ºC. Counter-electrode: Pt; working electrode: Glassy Carbon; potential given in V *vs* Ag/AgCl; scan rate: 100 mV/s.

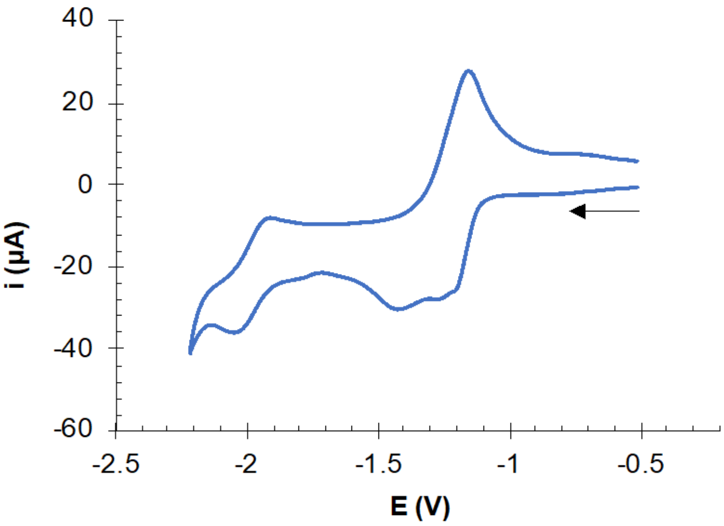

Figure S2. Cyclic voltammograms of a CH_2Cl_2 solution of compound **12** (10^{-3} M) containing 10^{-1} M $[NBu_4]PF_6$ as supporting electrolyte at 25 ºC. Counter-electrode: Pt; working electrode: Glassy Carbon; potential given in V vs Ag/AgCl; scan rate: 100 mV/s.

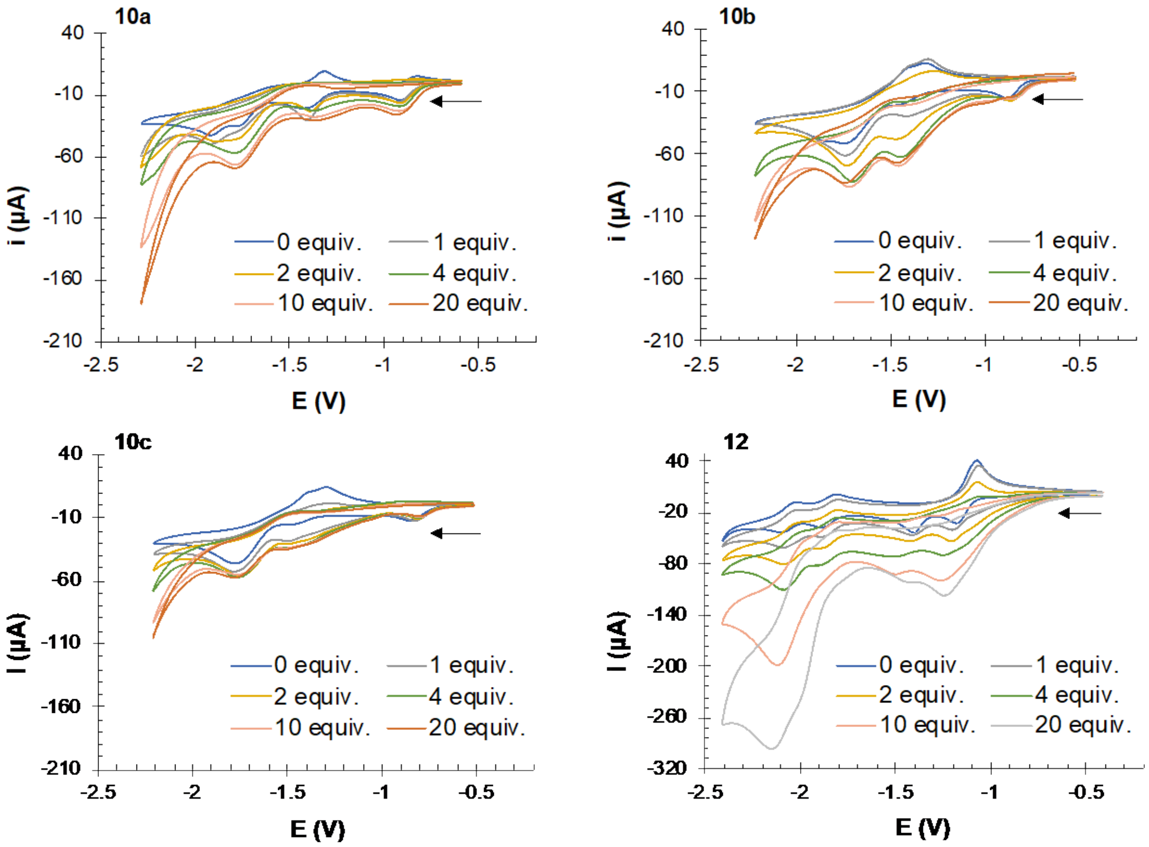

Figure S3. Electrochemical response of complexes **10a**, **10b** and **10c** in CH_2Cl_2 (10^{-3} M) and **12** in CH_3CN (10^{-3} M) with increasing amounts of acetic acid (0-20 equiv.). Cyclic voltammograms registered at 25 ºC. Supporting electrolyte: $[NBu_4]PF_6$ (10^{-1} M). Counter-electrode: Pt; working electrode: Glassy Carbon; potential given in V vs Fc^+/Fc; scan rate: 100 mV/s

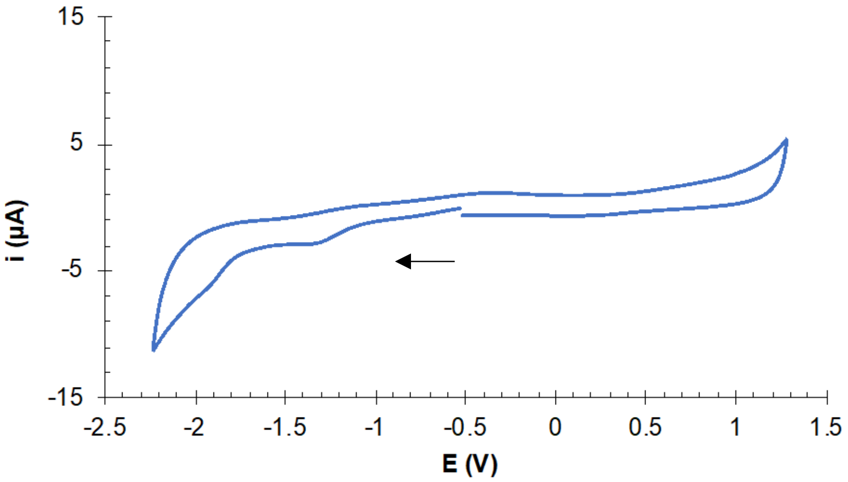

Figure S4. Cyclic voltammogram of a CH_2Cl_2 solution of AcOH (10^{-2} M) containing 10^{-1} M $[NBu_4]PF_6$ as supporting electrolyte at 25 ºC. Counter-electrode: Pt; working electrode: Glassy Carbon; potential given in V *vs* Ag/AgCl; scan rate: 100 mV/s.

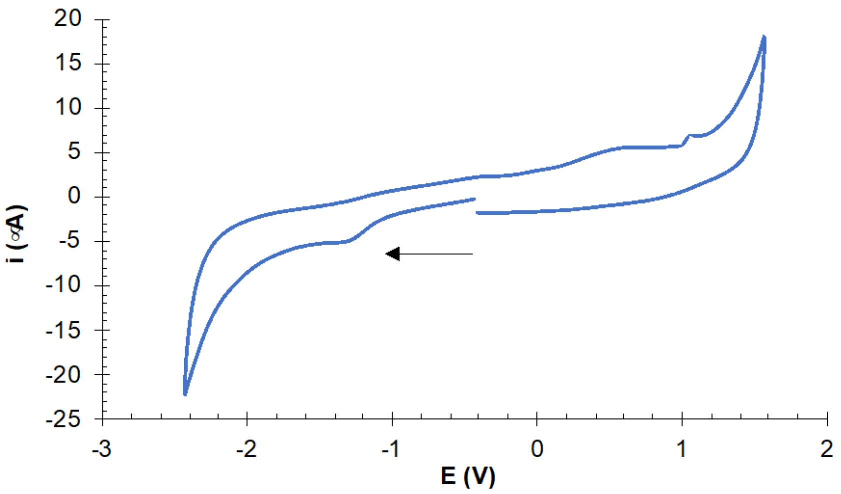

Figure S5. Cyclic voltammogram of a MeCN solution of AcOH (10^{-2} M) containing 10^{-1} M $[NBu_4]PF_6$ as supporting electrolyte at 25 °C. Counter-electrode: Pt; working electrode: Glassy Carbon; potential given in V *vs* Ag/AgCl; scan rate: 100 mV/s.

S.3. Crystal data

Crystal data for [compound 6]$_2$EtOH

$(C_{34}H_{14}Fe_4N_4O_{10}S_4)_2(C_2H_6O_2)$, M = 2026.36, monoclinic, $C_{2/c}$, a = 46.810(2), b = 7.5034(3), c = 12.4963(8) Å, β = 97.593(5)°, V = 4350.6(4) Å^3, Z = 2, T = 120(2) K, λ = 0.71073 Å, Dcalc = 1.547 g/cm^3, µ = 1.556 cm^{-1}, 19577 reflections measured, 6865 unique (Rint = 0.0866), dark red plate of 0.16 x 0.14 x 0.02 mm size, crystal structure solved by dual space methods with all non-hydrogen atoms refined anisotropically on F^2 using the programs SHELXT-2018 and SHELXL-2019.[2,3] Hydrogen atoms were included using a riding model. Both the bipyridinium ligand and the C(35)-O(35) carbonyl ligand are disordered over an inversion center and were refined with half occupancy. Residual electron density associated with an inversion center was tentatively identified as a disordered molecule of ethanol and refined with quarter occupancy and restrained C-O distance (command DFIX). The H atom at O97 should be considered as tentative as there is no indication of H bonding. Disordered moieties were refined with appropriate similarity restraints (command SAME) and local ring geometry restraints (FLAT, SAME). Disordered atoms U value components were restrained to be equal (commands RIGU, ISOR). GOF = 1.051, R (Fo, I > 2σ(I)) = 0.0675, Rw (Fo^2, all data) = 0.1882.

S.4. NMR spectra

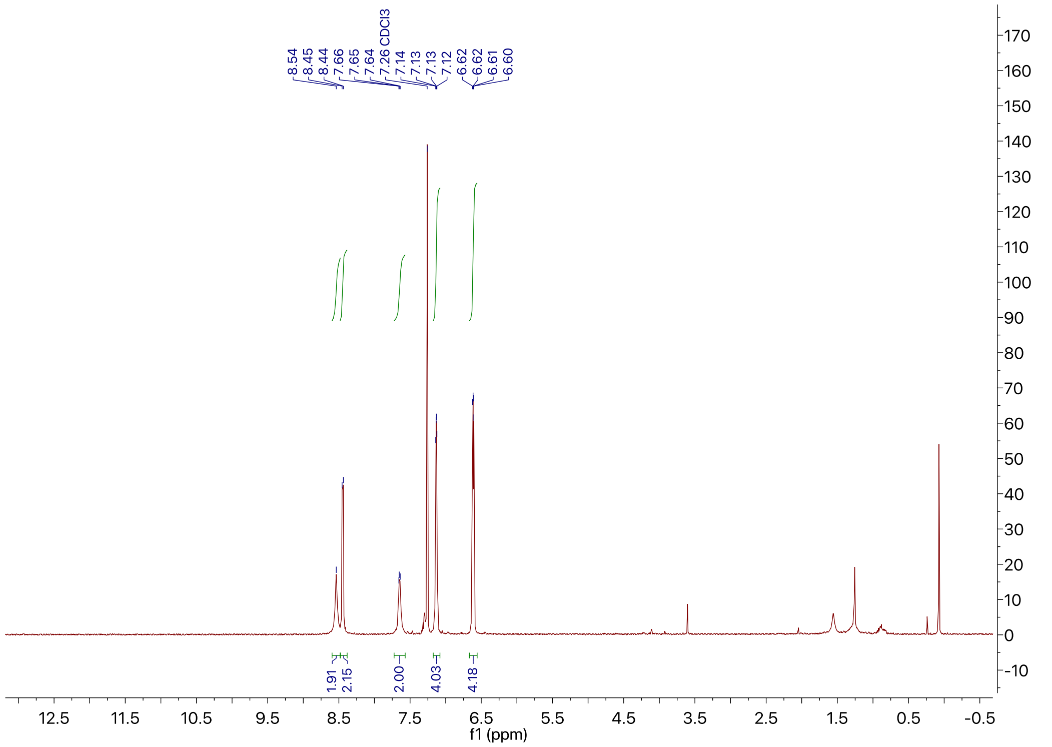

Figure S6. ^{1}H NMR (500 MHz), $CDCl_3$, compound **6**.

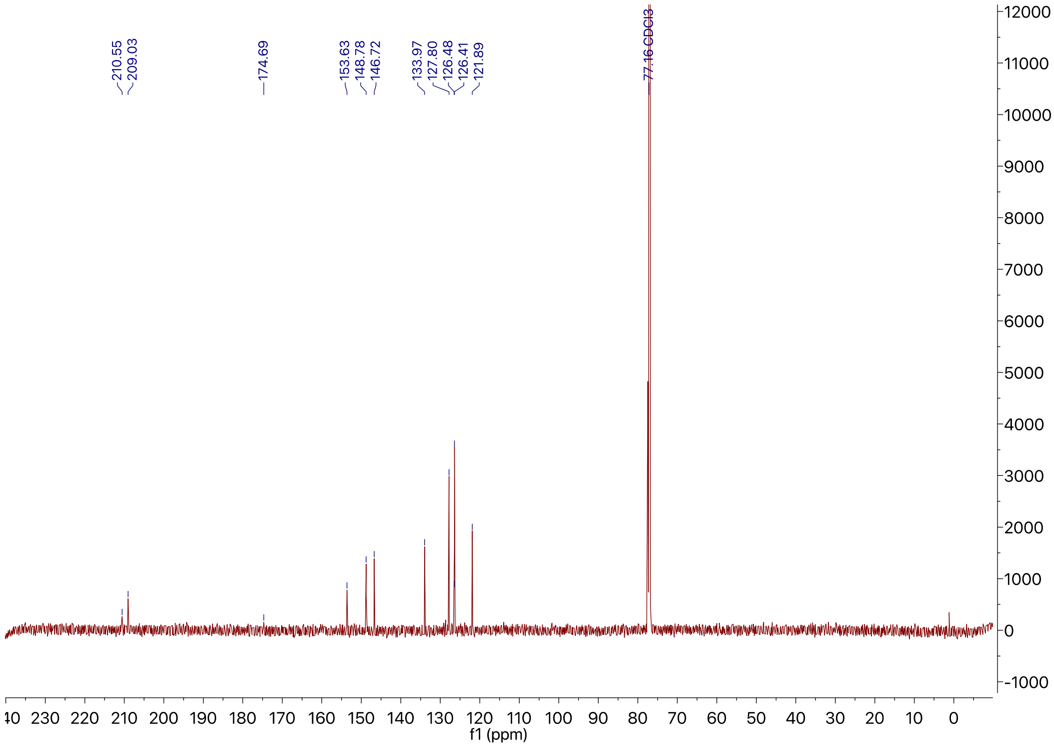

Figure S7. $^{13}C\{^{1}H\}$ NMR (126 MHz), $CDCl_3$, compound **6**.

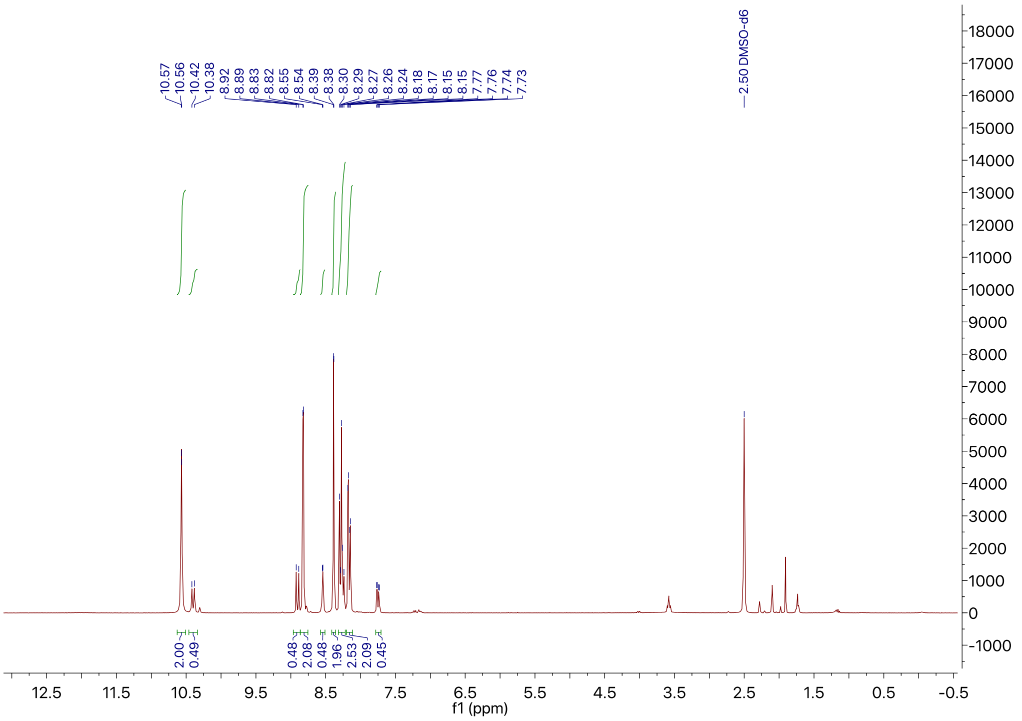

Figure S8. ^{1}H NMR (300 MHz), DMSO-d_6, compound **7**.

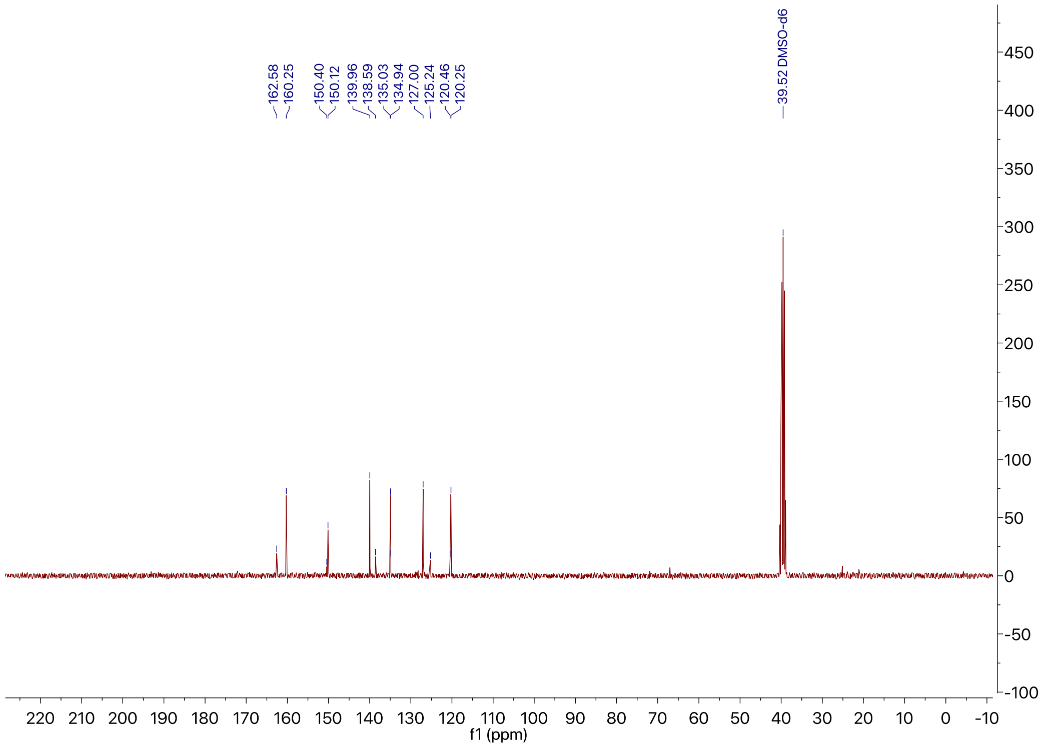

Figure S9. ^{13}C{^{1}H} NMR (75 MHz), DMSO-d_6, compound **7**.

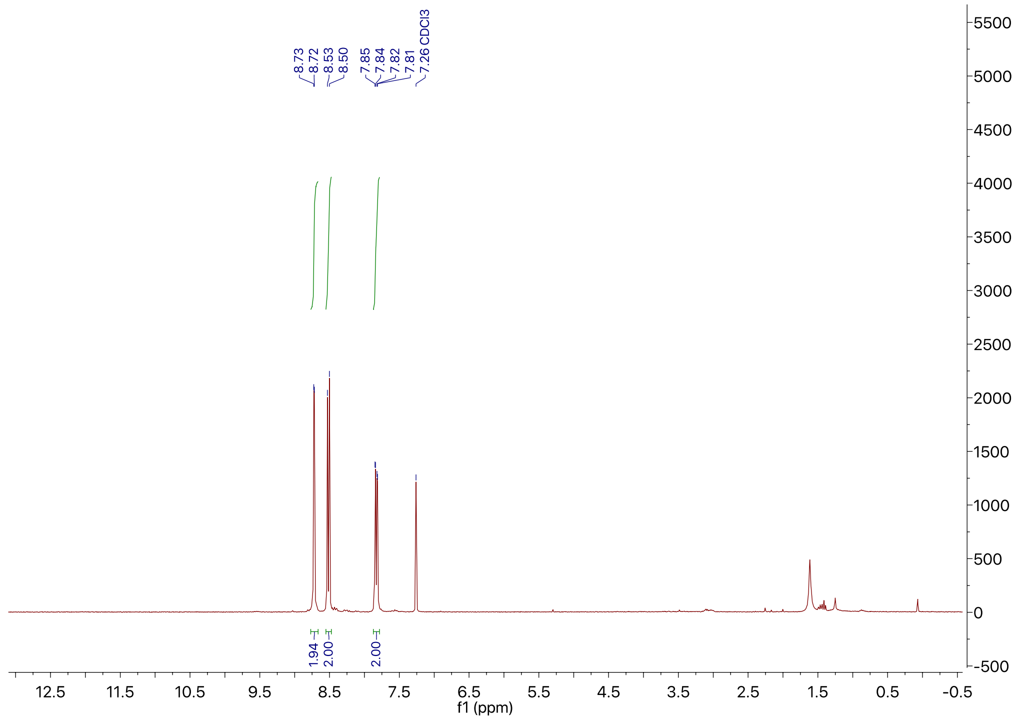

Figure S10. ^{1}H NMR (300 MHz), $CDCl_3$, compound **8**.

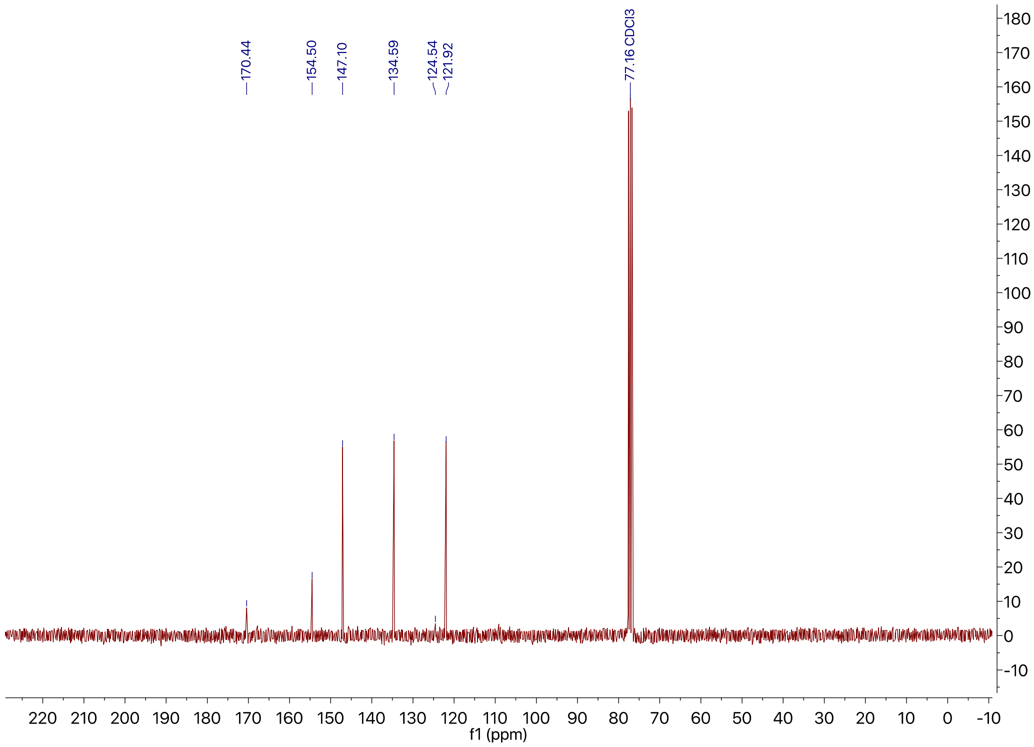

Figure S11. $^{13}C\{^{1}H\}$ NMR (75 MHz), $CDCl_3$, compound **8**.

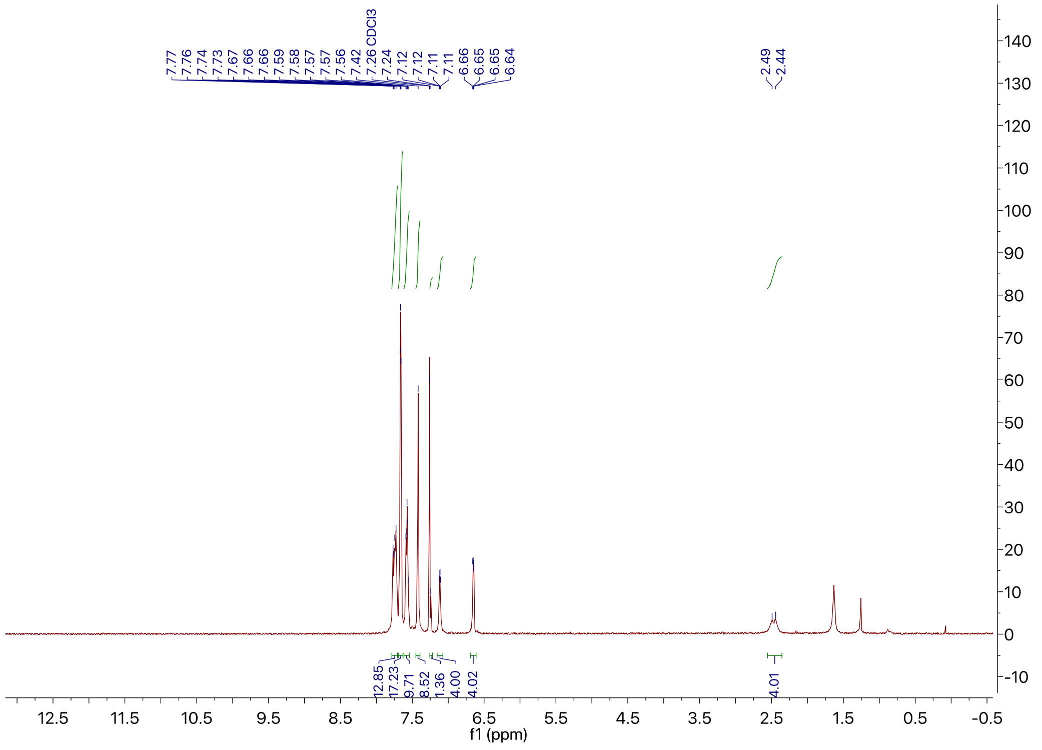

Figure S12. ^{1}H NMR (500 MHz), $CDCl_3$, compound **10a**.

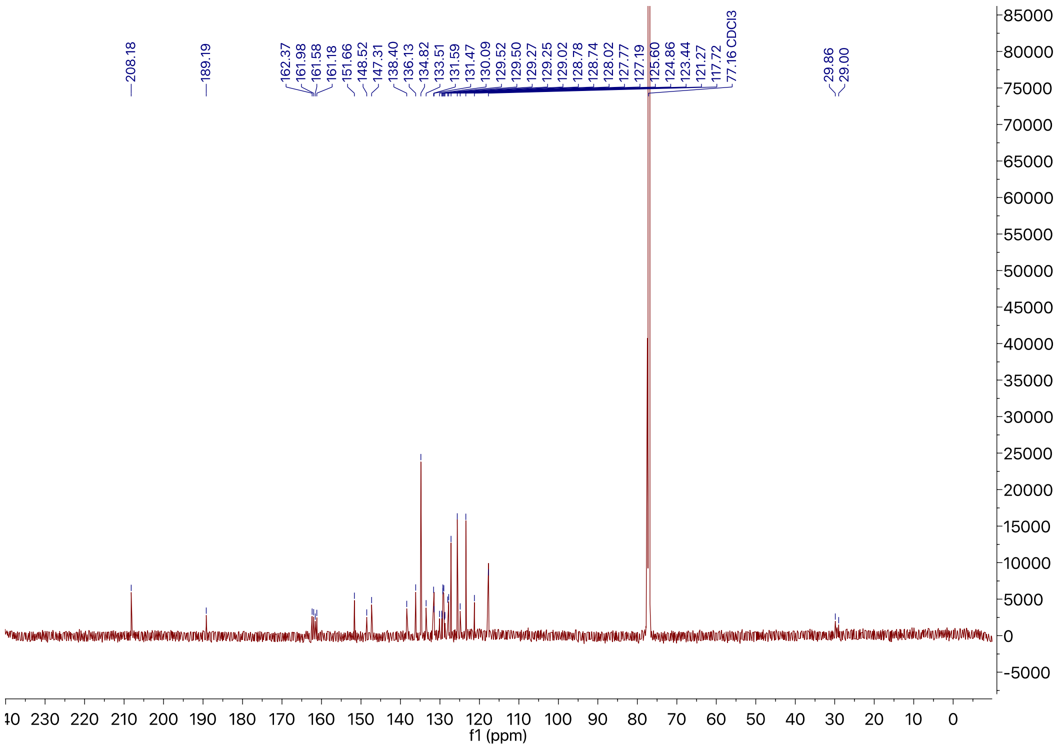

Figure S13. $^{13}C\{^{1}H\}$ NMR (126 MHz), $CDCl_3$, compound **10a**.

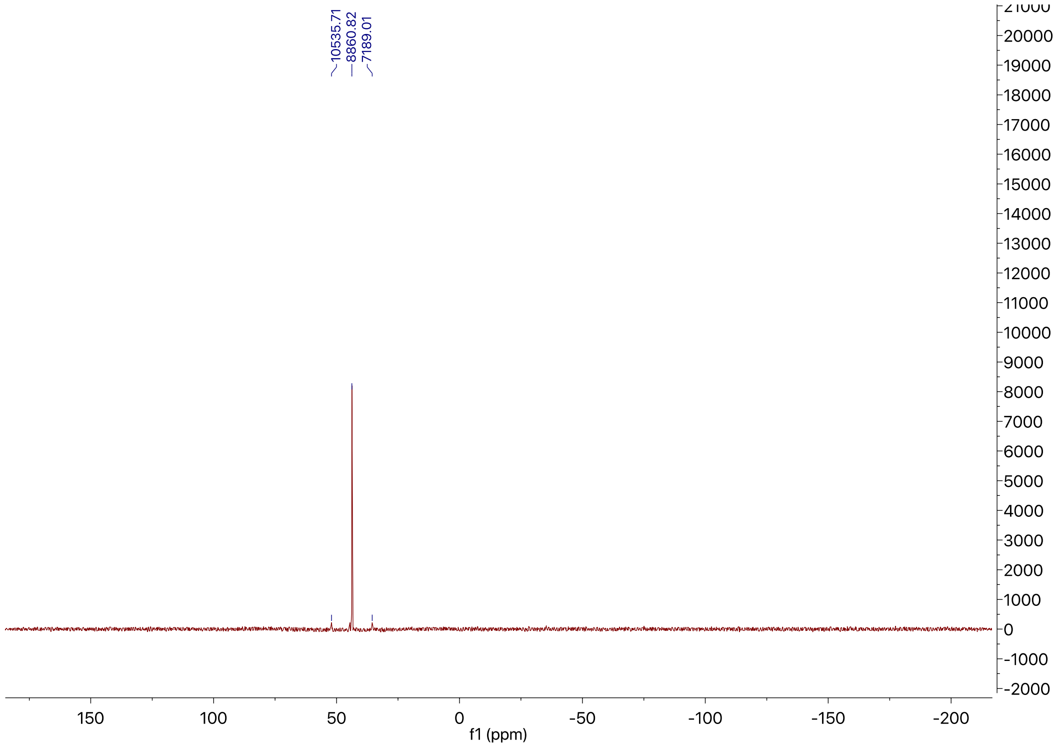

Figure S14. $^{31}P\{^1H\}$ NMR (202 MHz), $CDCl_3$, compound **10a**.

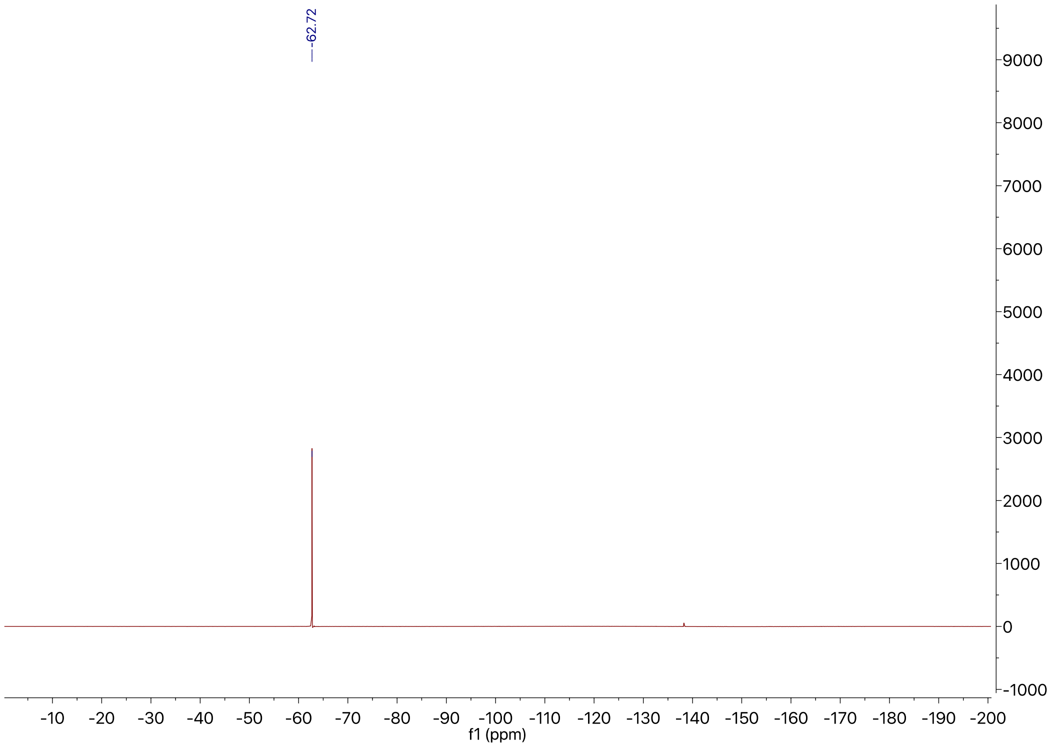

Figure S15. $^{19}F\{^1H\}$ NMR (471 MHz), $CDCl_3$, compound **10a**.

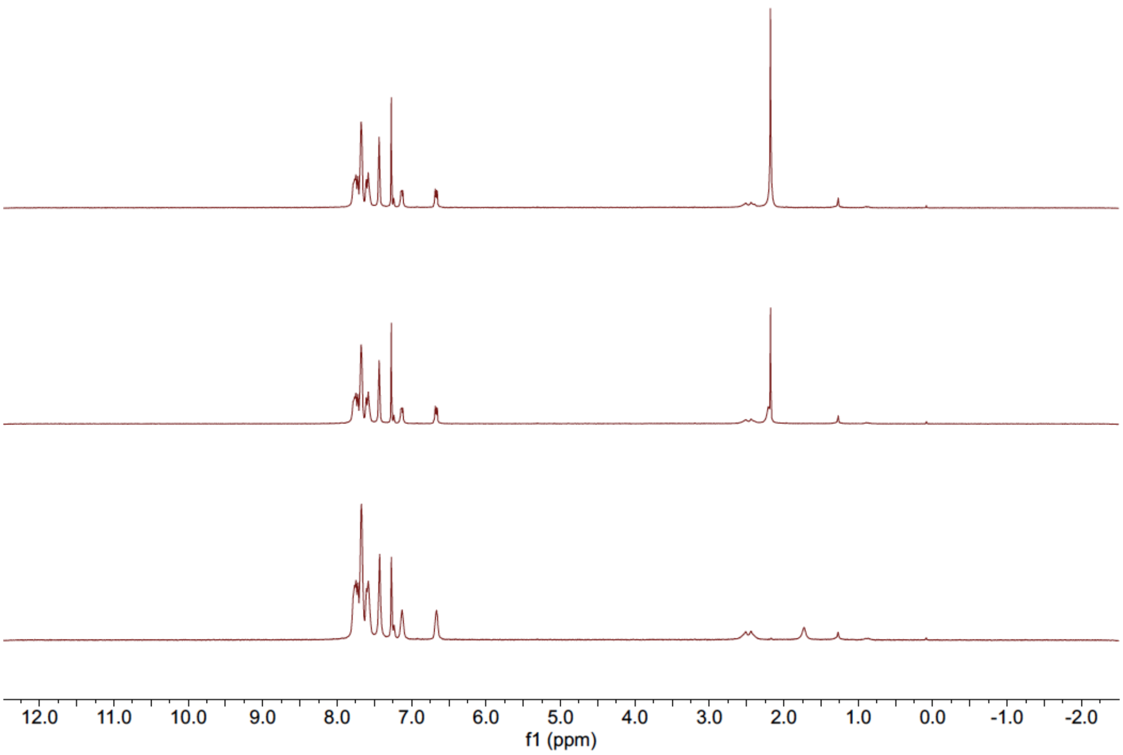

Figure S16. ^{1}H NMR (300 MHz) spectra of compound **10a** ($CDCl_3$) with AcOH (0 to 4 equiv.).

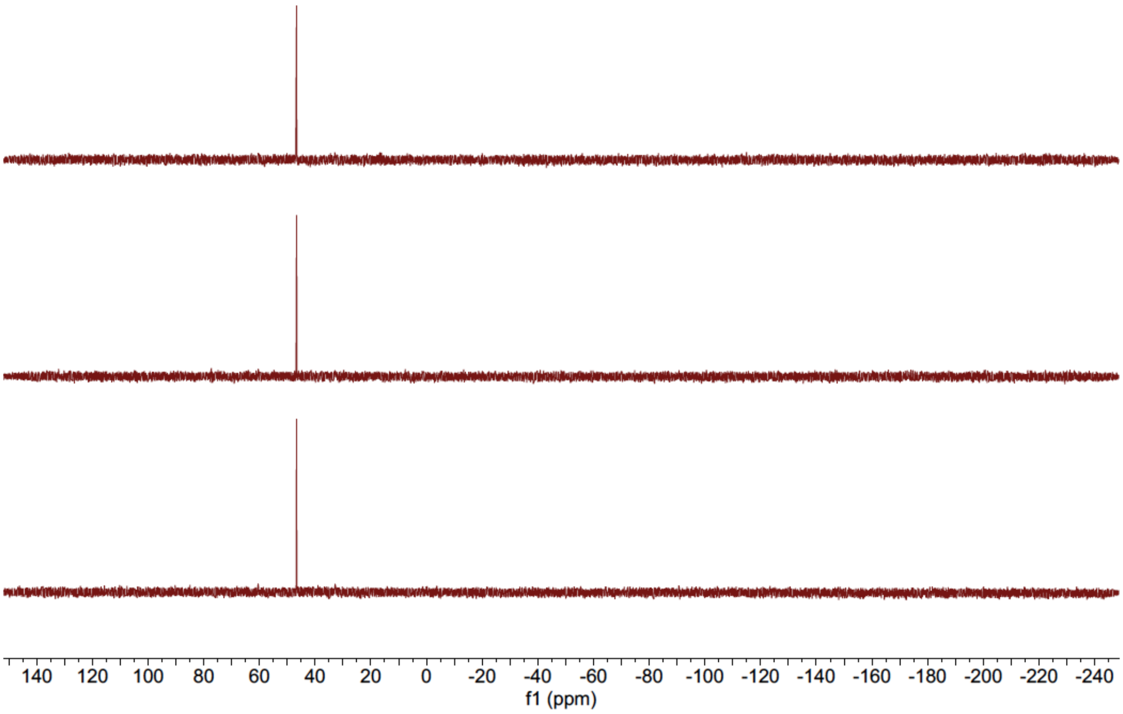

Figure S17. $^{31}P\{^{1}H\}$ NMR (122 MHz) spectra of compound **10a** ($CDCl_3$) with AcOH (0 to 4 equiv.).

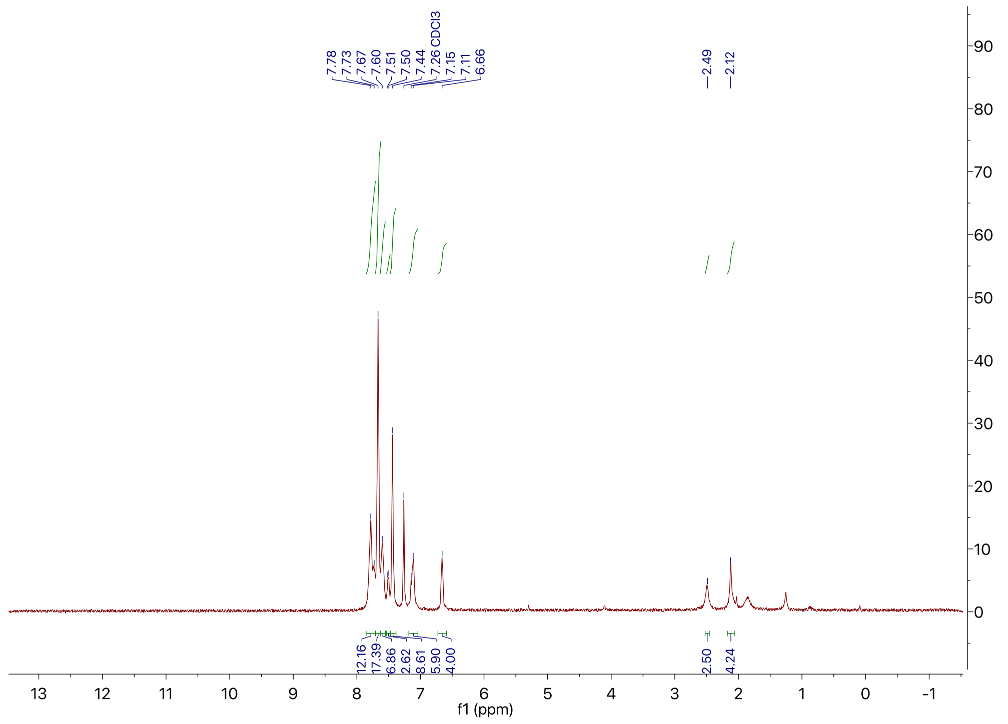

Figure S18. ^{1}H NMR (500 MHz), $CDCl_3$, compound **10b**.

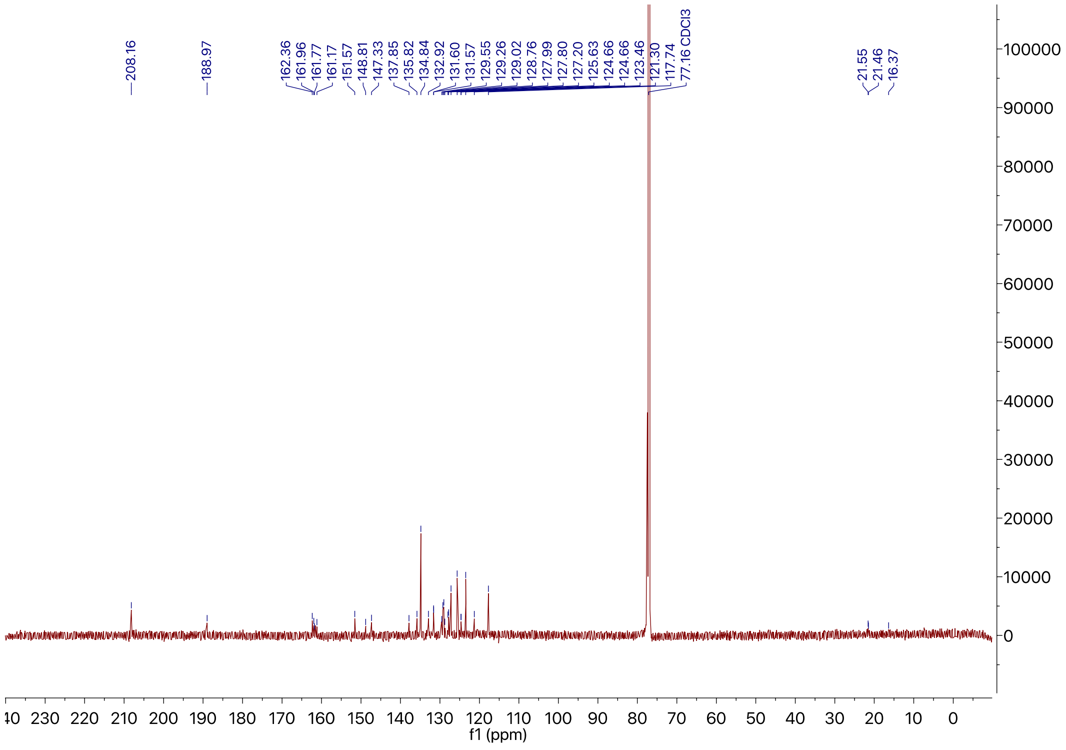

Figure S19. $^{13}C\{^{1}H\}$ NMR (126 MHz), $CDCl_3$, compound **10b**.

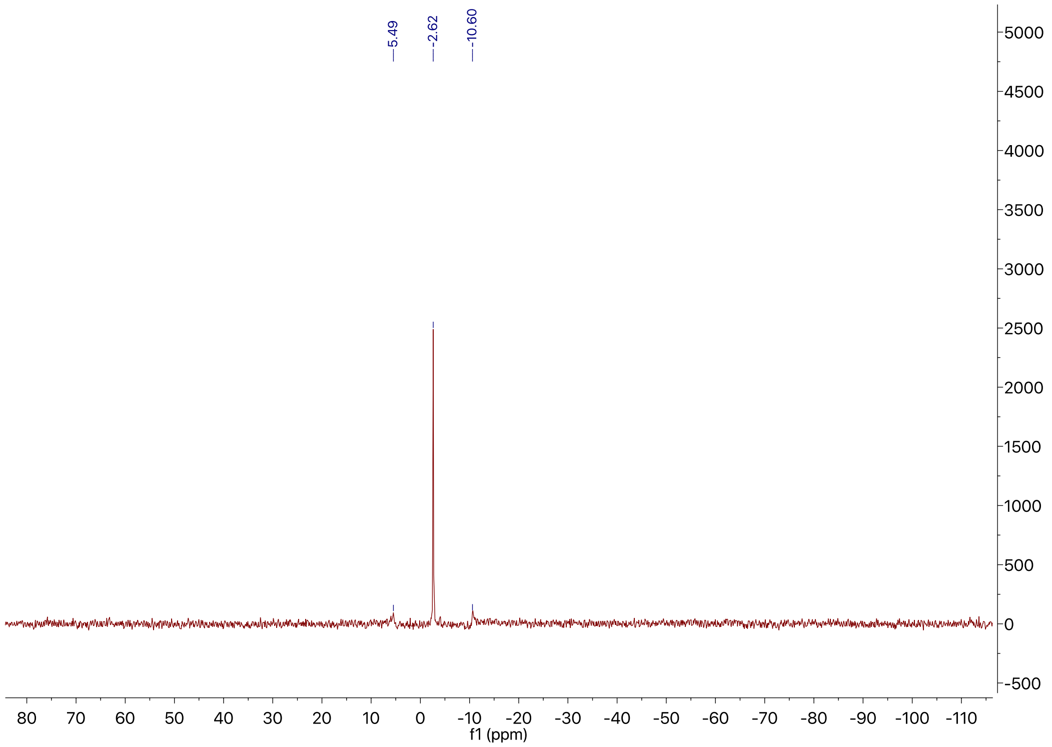

Figure S20. $^{32}P\{^{1}H\}$ NMR (202 MHz), $CDCl_3$, compound **10b**.

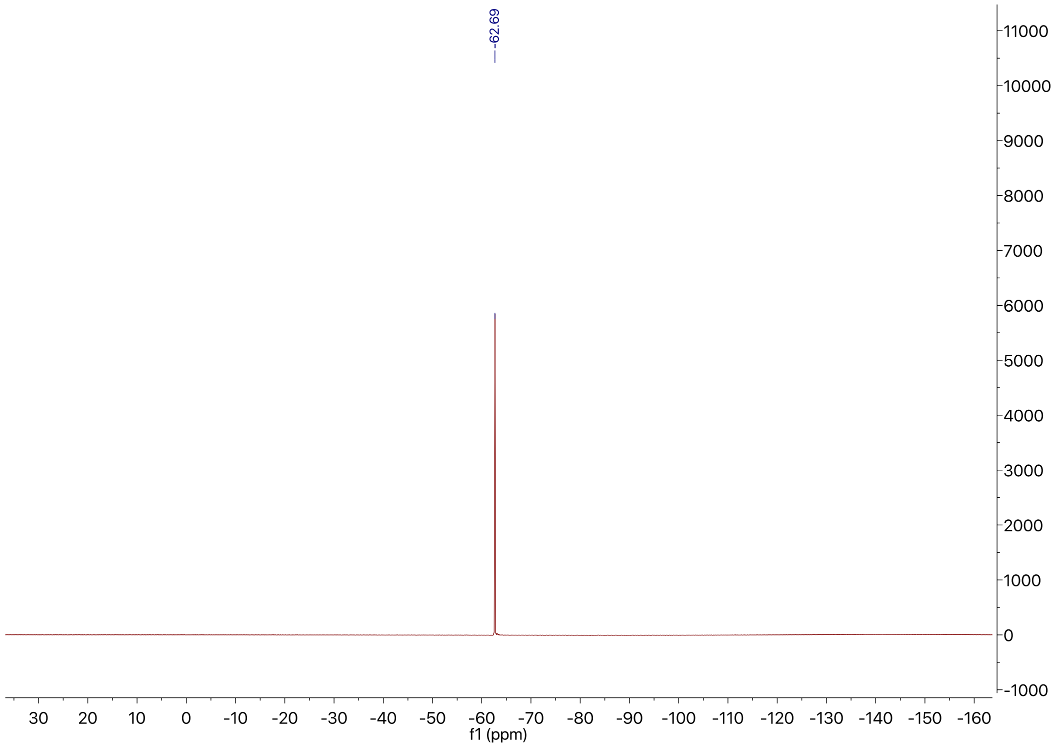

Figure S21. $^{19}F\{^{1}H\}$ NMR (471 MHz), $CDCl_3$, compound **10b**.

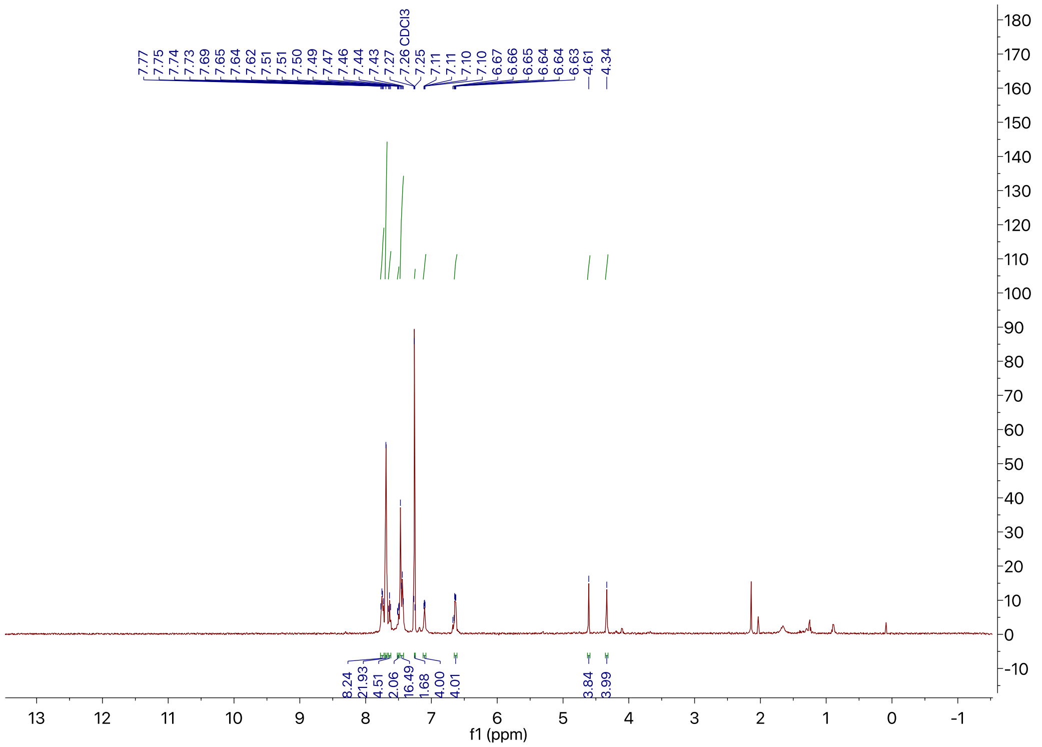

Figure S22. ^{1}H NMR (500 MHz), $CDCl_3$, compound **10c**.

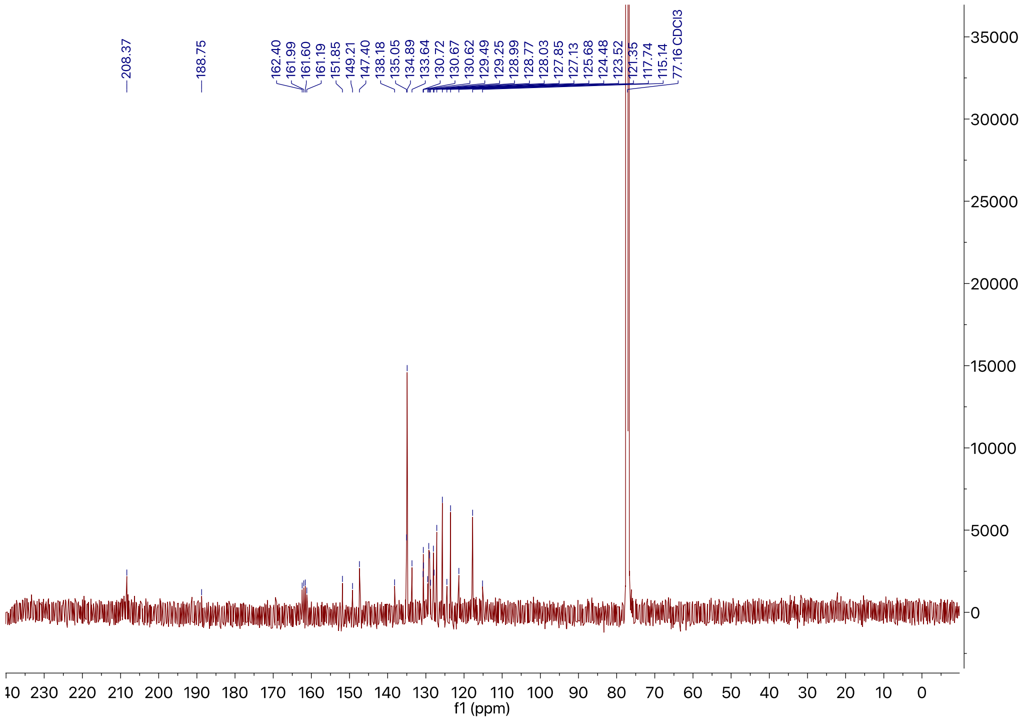

Figure S23. $^{13}C\{^{1}H\}$ NMR (126 MHz), $CDCl_3$, compound **10c**.

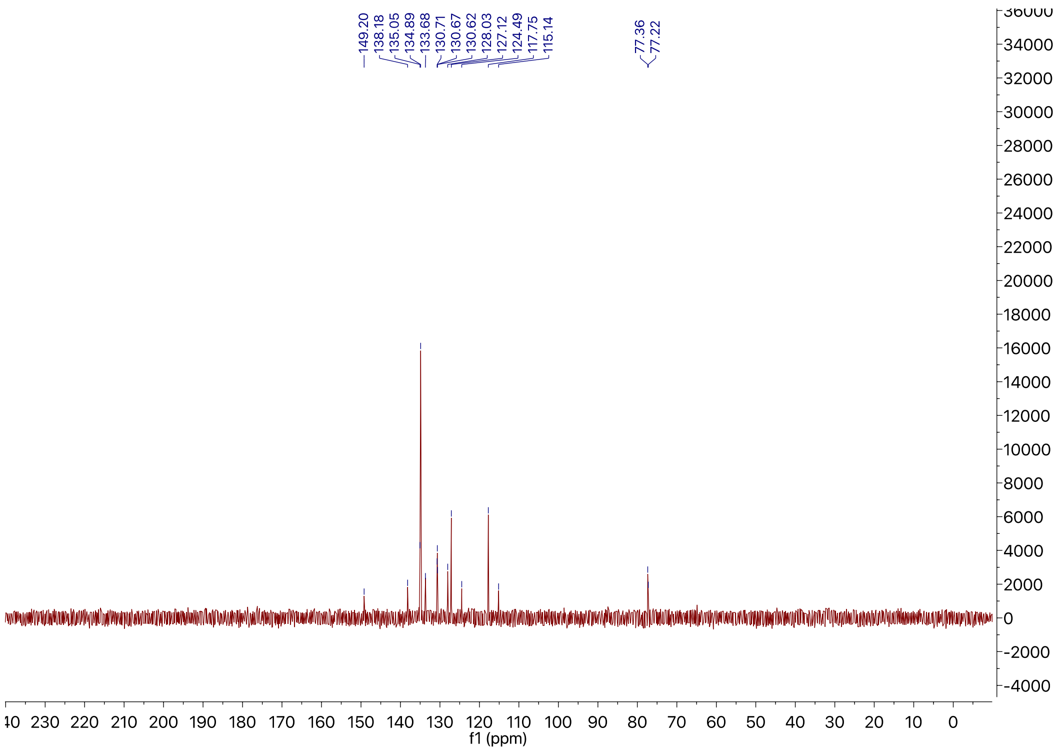

Figure S24. DEPT-135 $^{13}C\{^{1}H\}$ NMR (126 MHz), $CDCl_3$, compound **10c**.

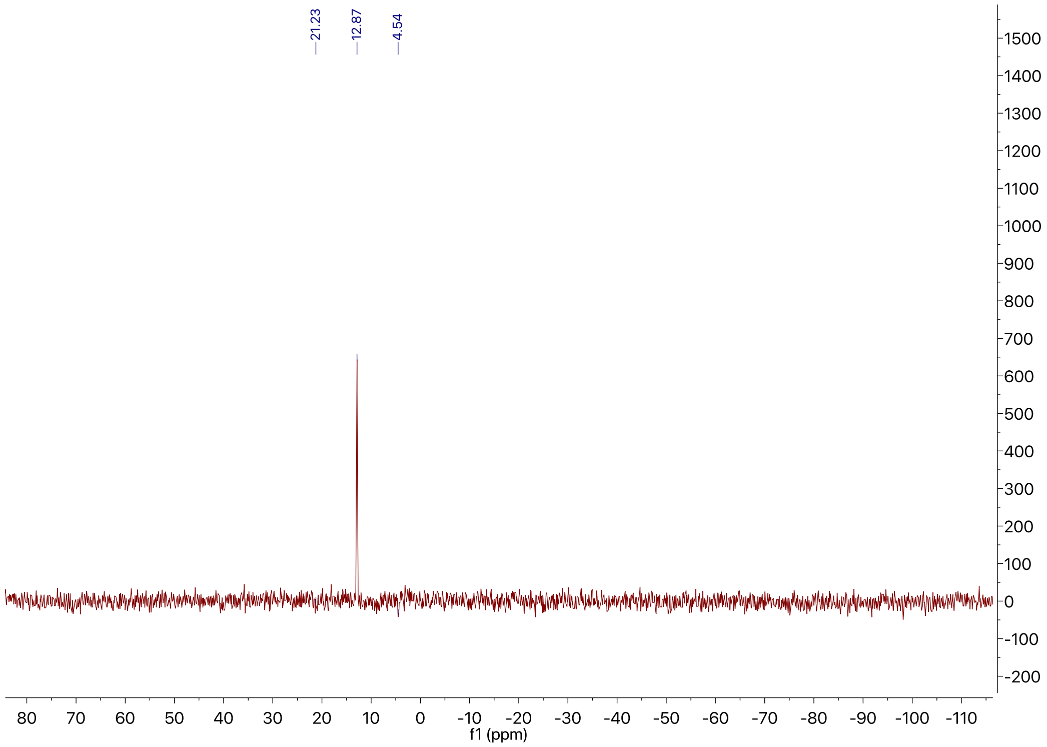

Figure S25. $^{31}P\{^{1}H\}$ NMR (202 MHz), $CDCl_3$, compound **10c**.

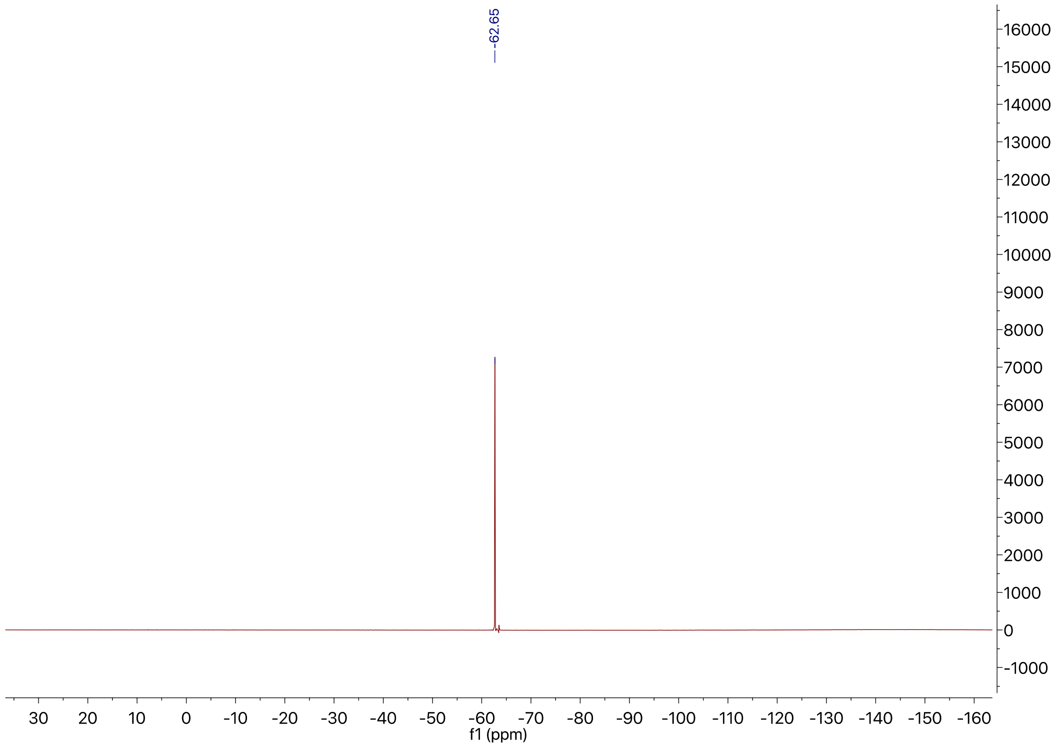

Figure S26. $^{19}F\{^{1}H\}$ NMR (471 MHz), $CDCl_3$, compound **10c**.

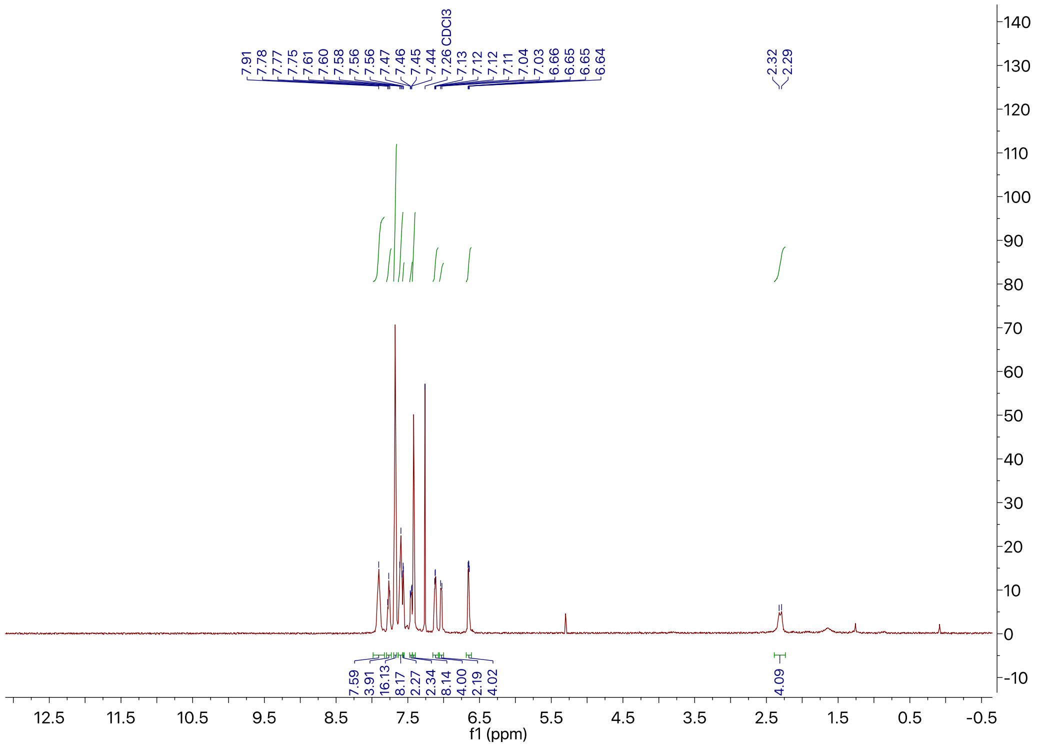

Figure S27. ^{1}H NMR (500 MHz), $CDCl_3$, compound **11**.

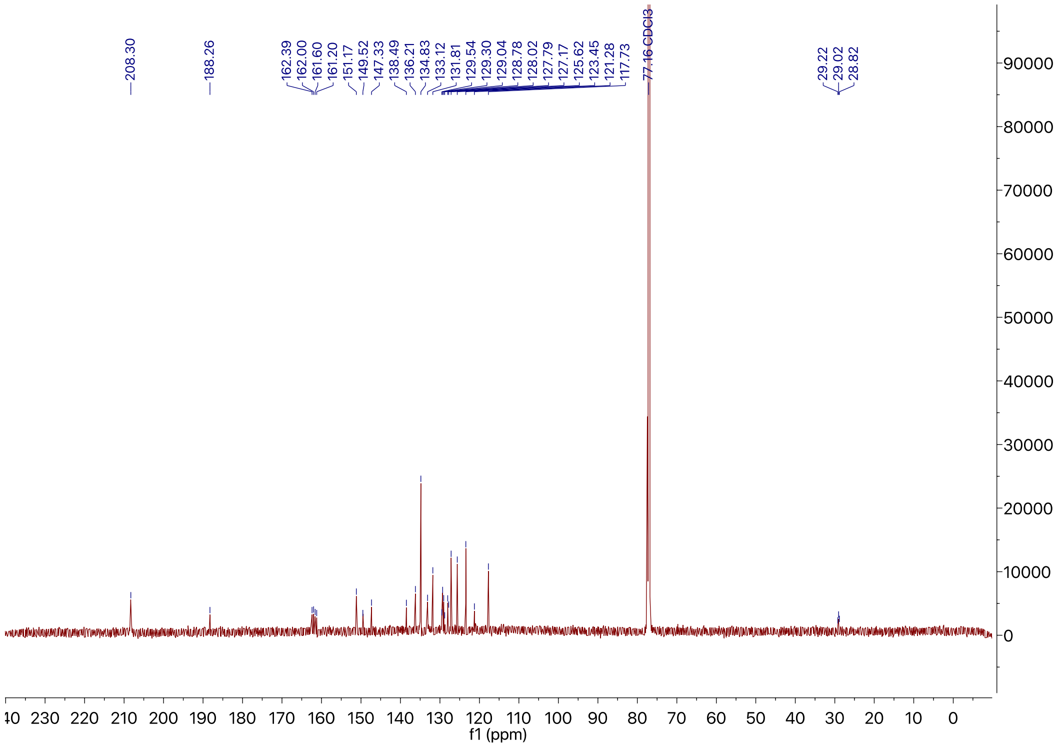

Figure S28. $^{13}C\{^{1}H\}$ NMR (126 MHz), $CDCl_3$, compound **11**.

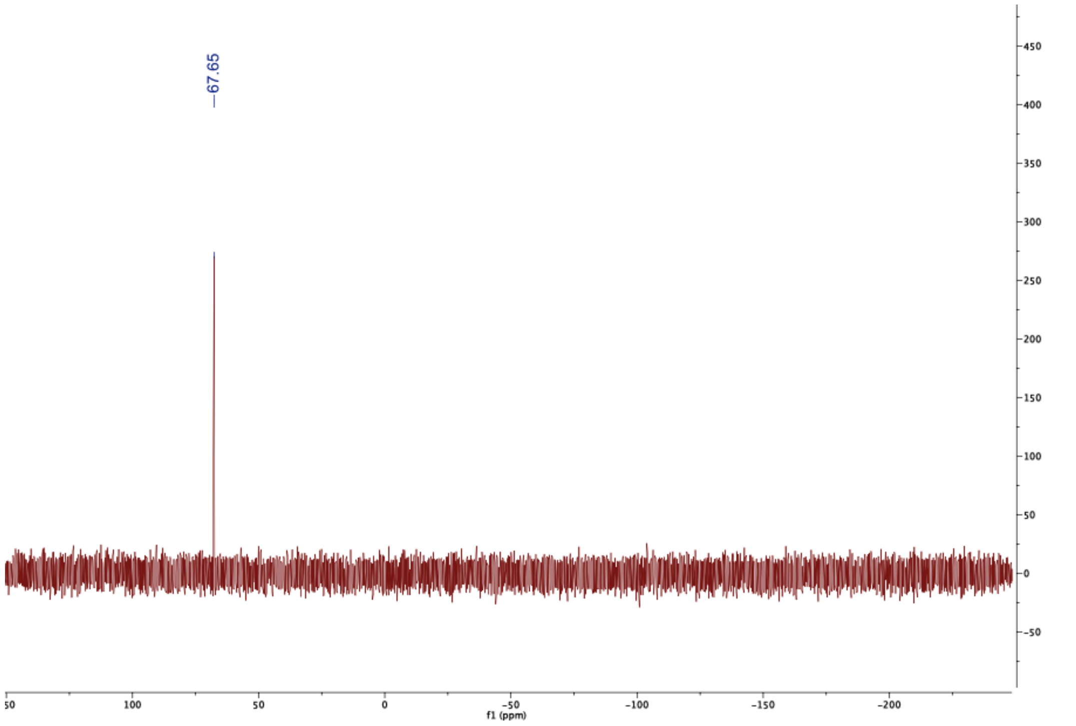

Figure S29. $^{31}P\{^{1}H\}$ NMR (202 MHz), $CDCl_3$, compound **11**.

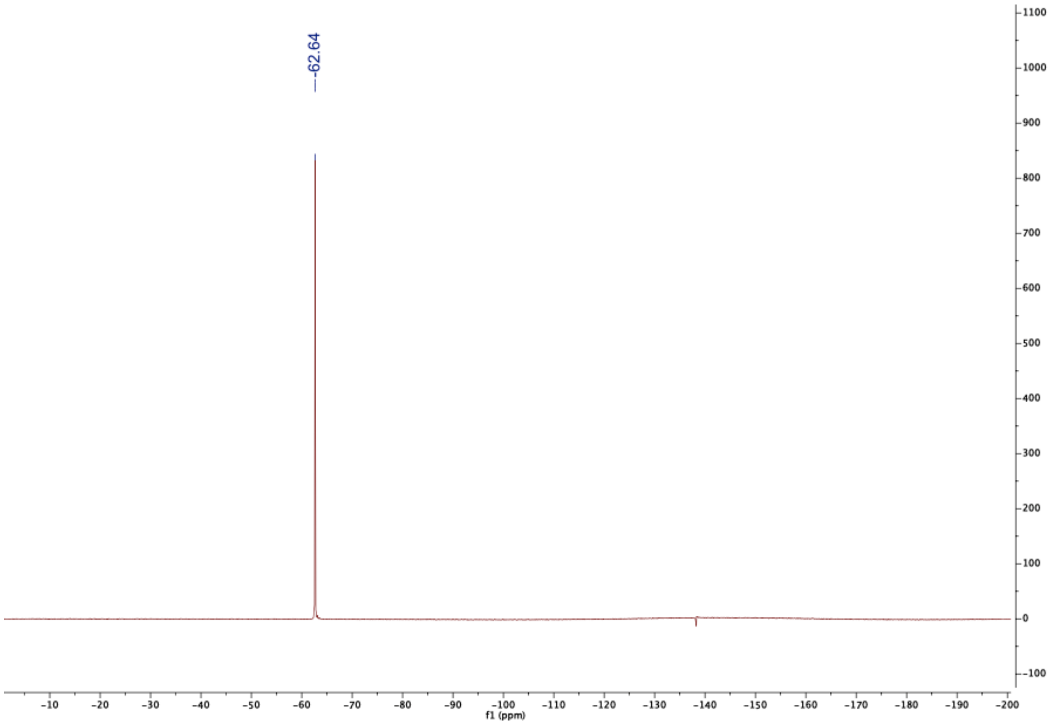

Figure S30. $^{19}F\{^{1}H\}$ NMR (471 MHz), $CDCl_3$, compound **11**.

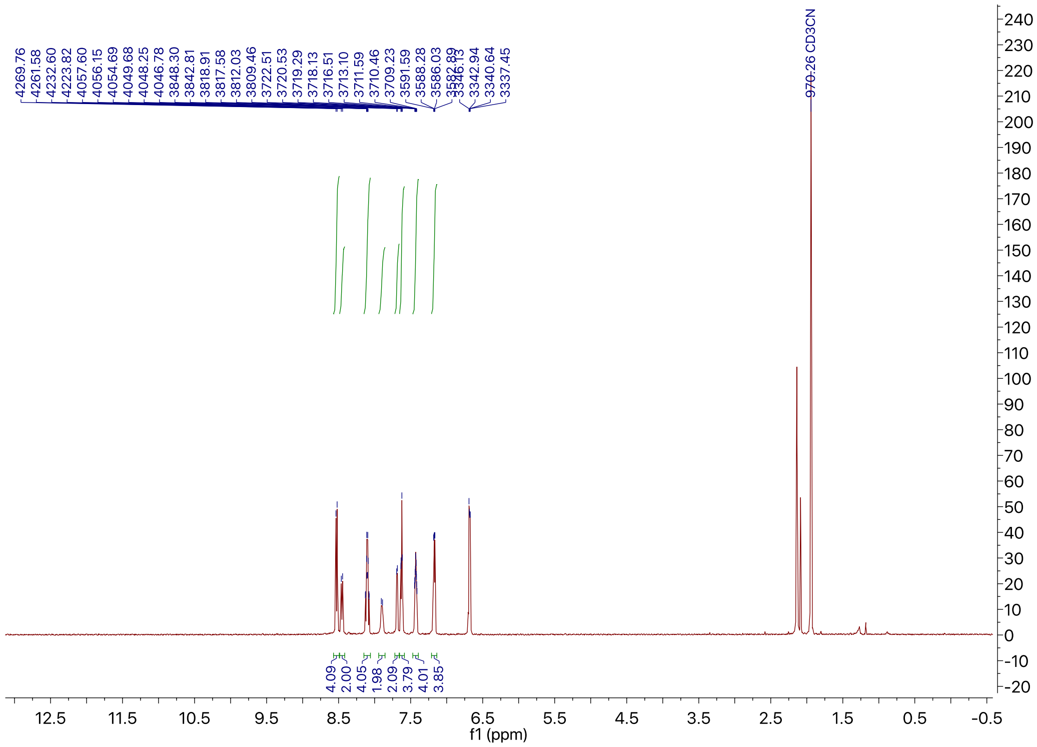

Figure S31. ^{1}H NMR (500 MHz), $CDCl_3$, compound **12**.

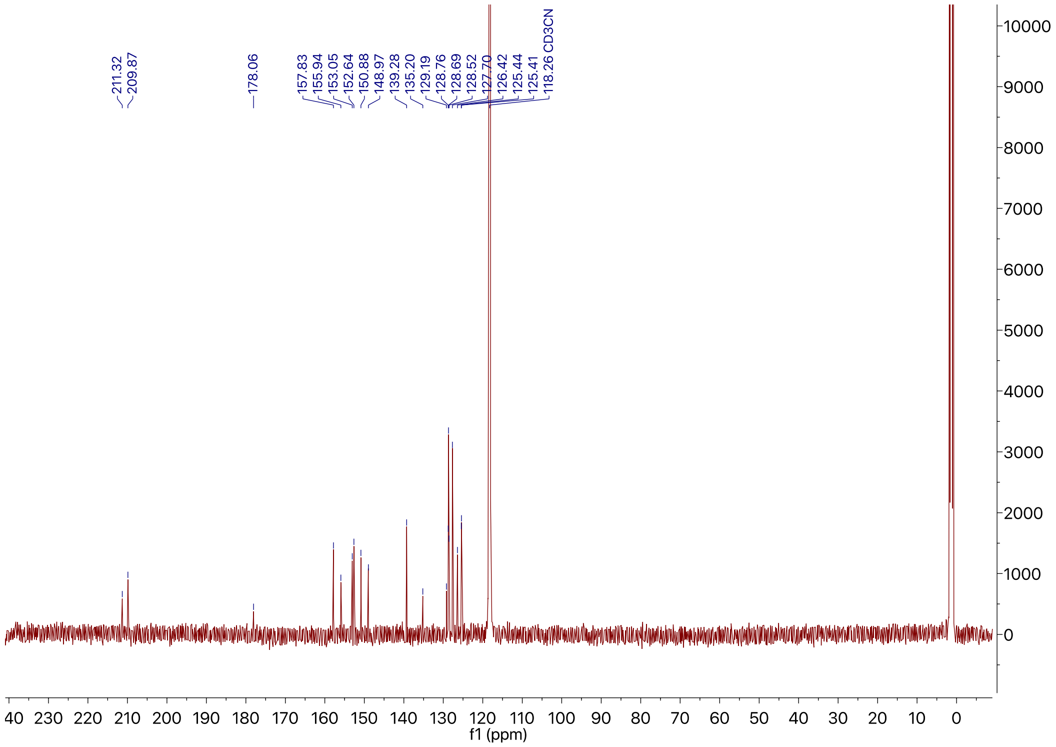

Figure S32. $^{13}C\{^{1}H\}$ NMR (126 MHz), $CDCl_3$, compound **12**.

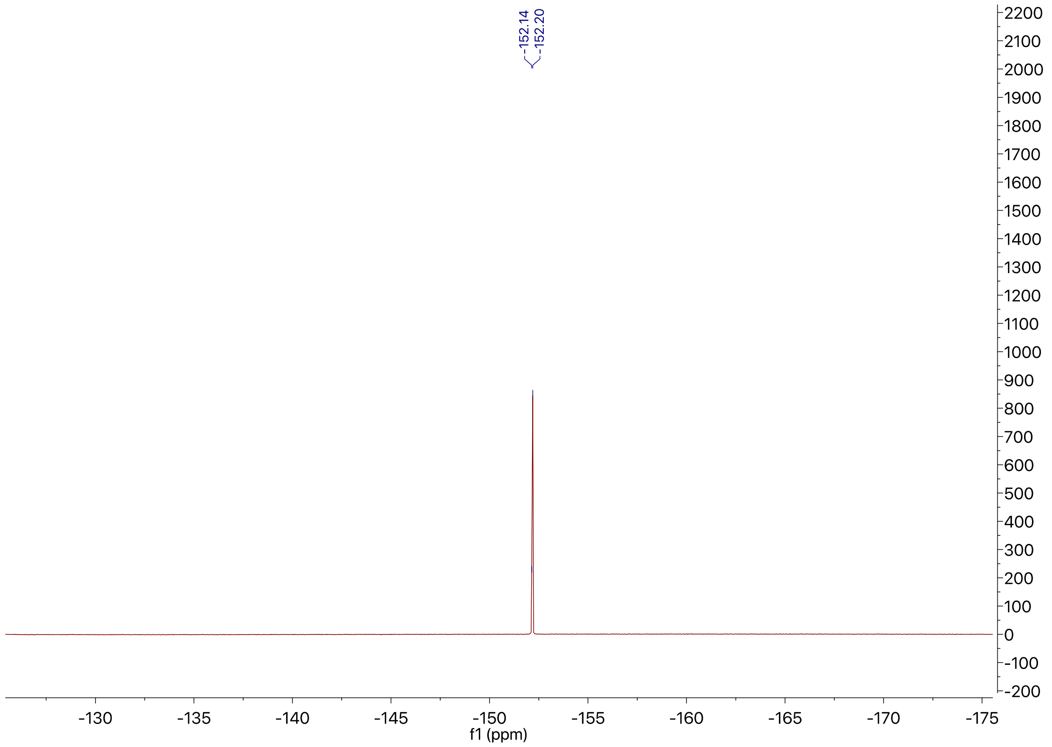

Figure S33. $^{19}F\{^{1}H\}$ NMR (471 MHz), $CDCl_3$, compound **12**.

S.5. FTIR spectra

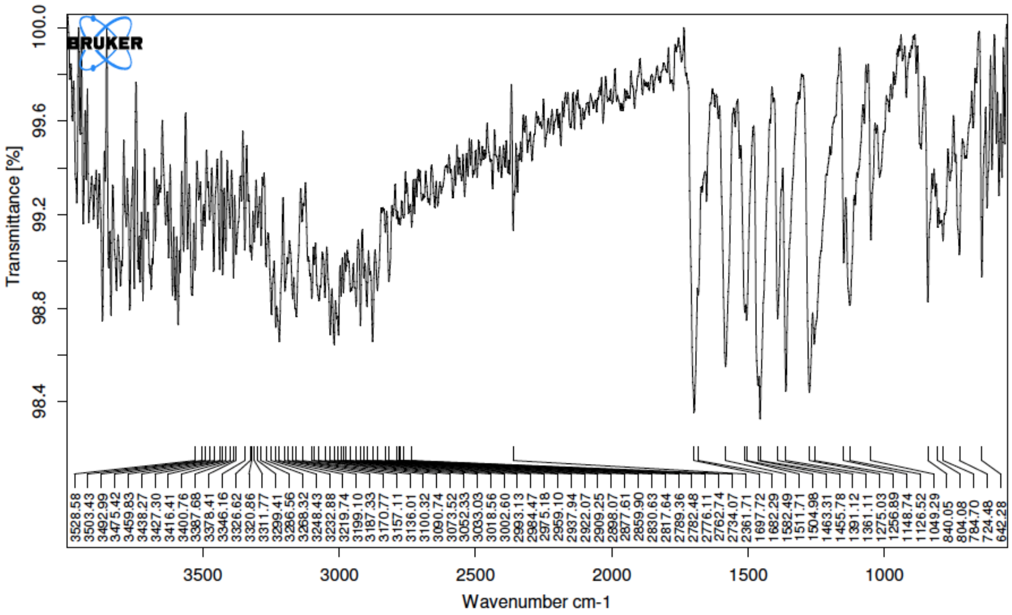

Figure S34. FTIR spectrum (film) of compound **7**.

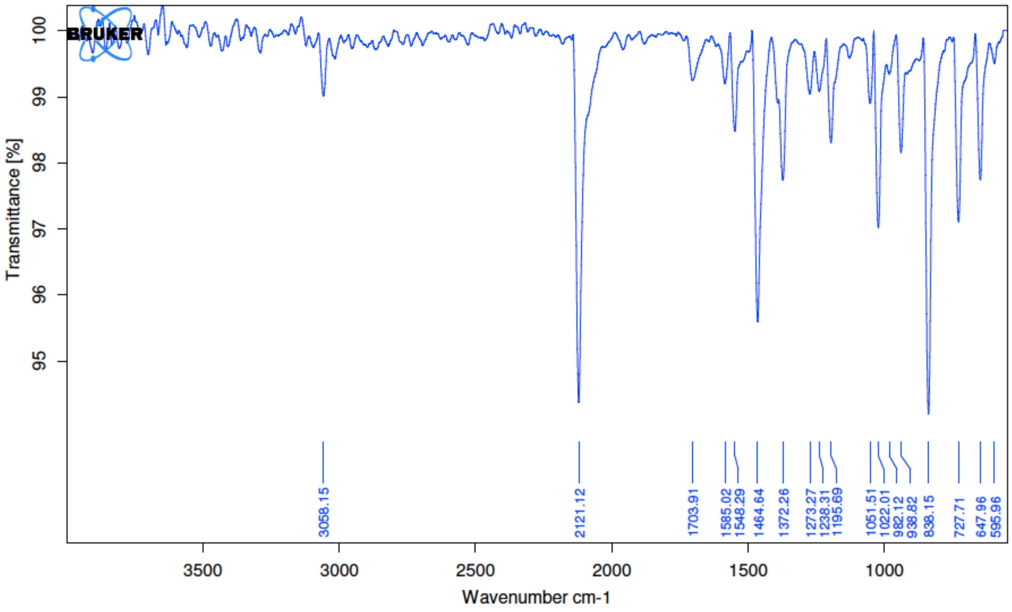

Figure S35. FTIR spectrum (film) of compound **8**.

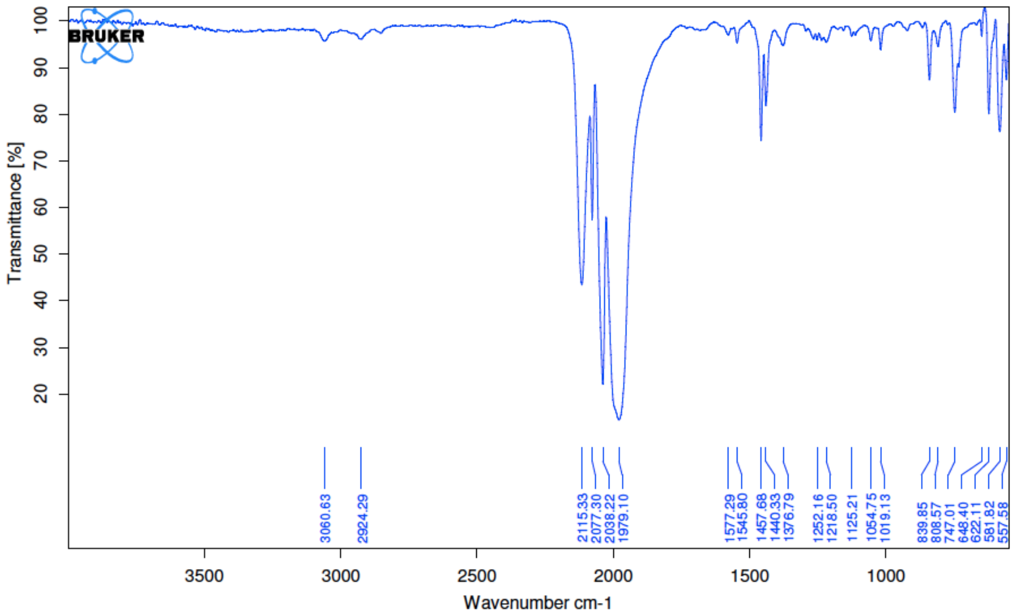

Figure S36. FTIR spectrum (film) of compound **6**.

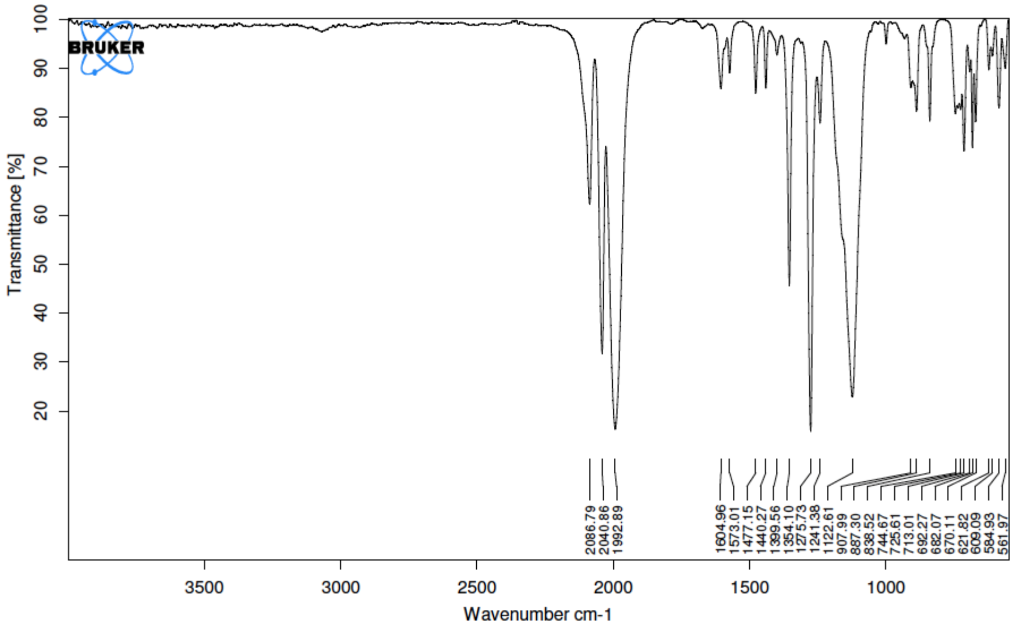

Figure S37. FTIR spectrum (film) of compound **10a**.

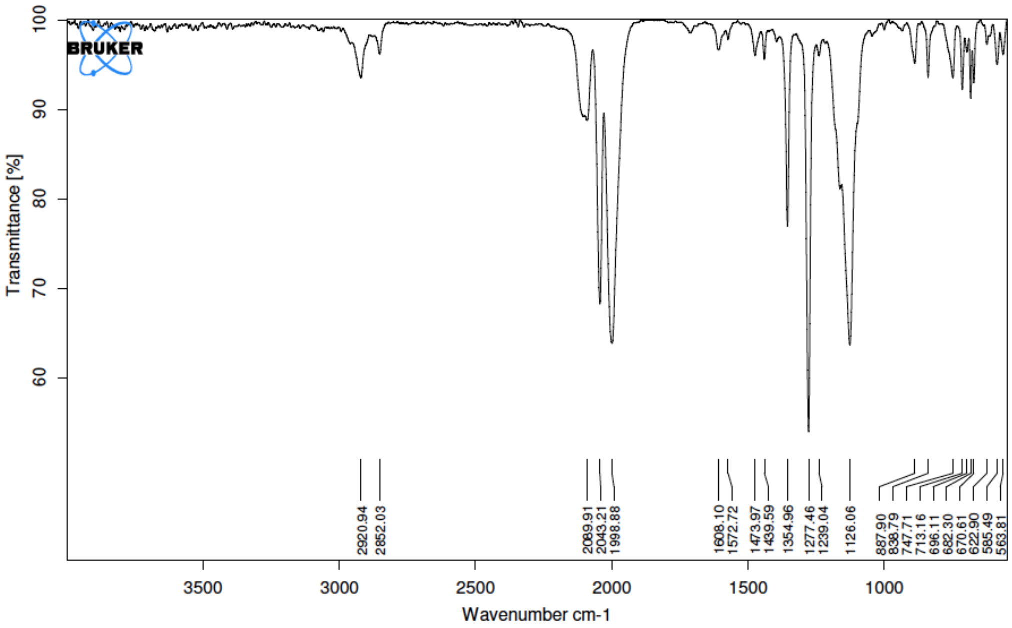

Figure S38. FTIR spectrum (film) of compound **10b**.

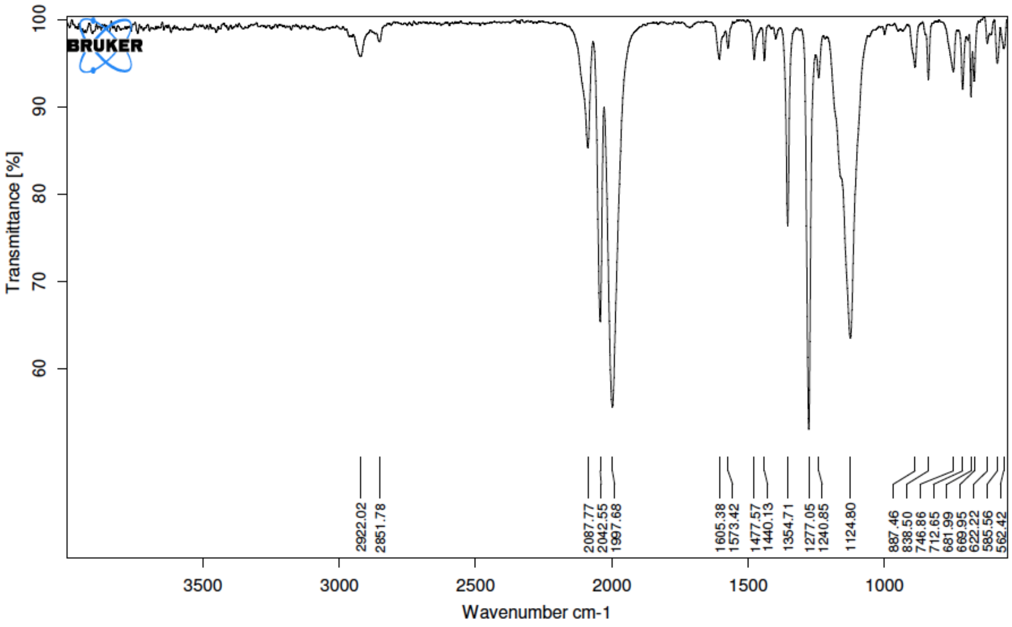

Figure S39. FTIR spectrum (film) of compound **10c**.

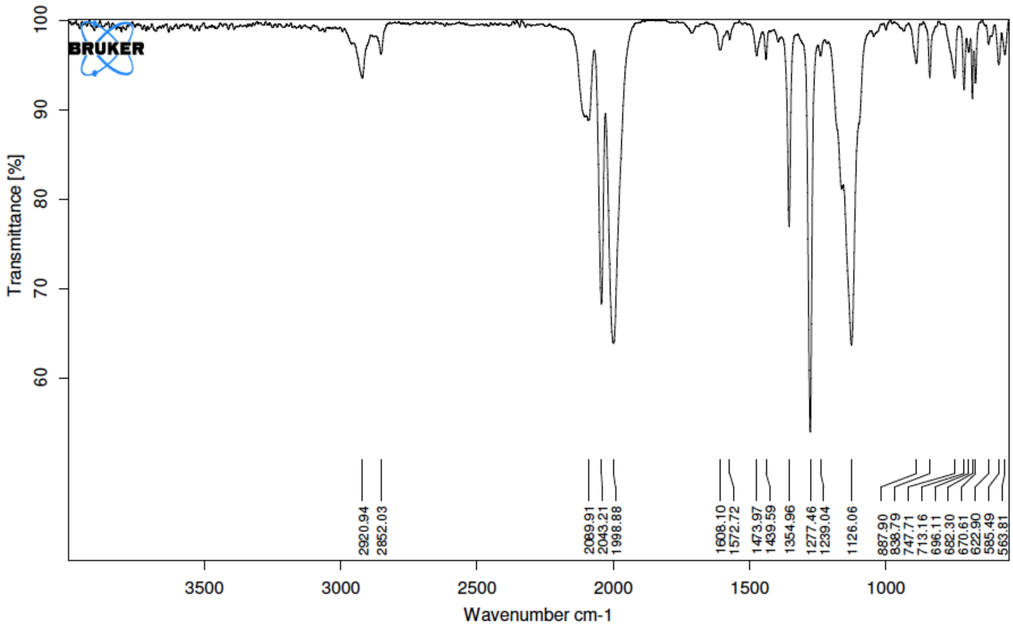

Figure S40. FTIR spectrum (film) of compound **11**.

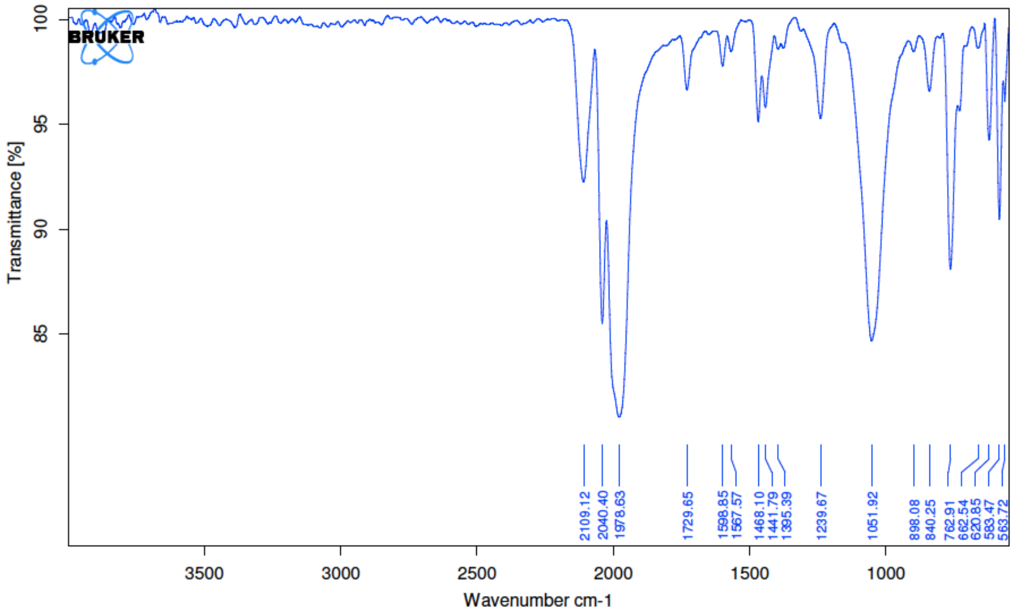

Figure S41. FTIR spectrum (film) of compound **12**.

S.6. DFT calculations

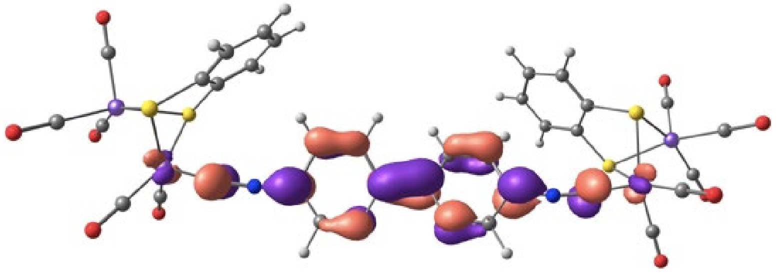

Figure S42. Computed LUMO of complex **6** (SMD(CH_2Cl_2)-B3LYP-D3/def2-SVP)

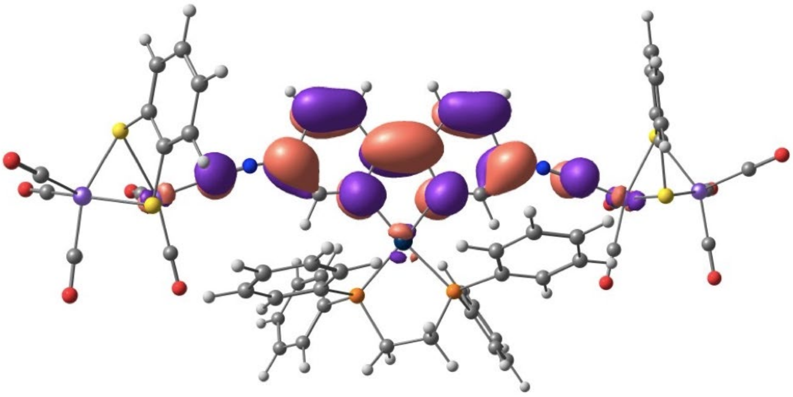

Figure S43. Computed LUMO of complex **10a** (SMD(CH_2Cl_2)-B3LYP-D3/def2-SVP).

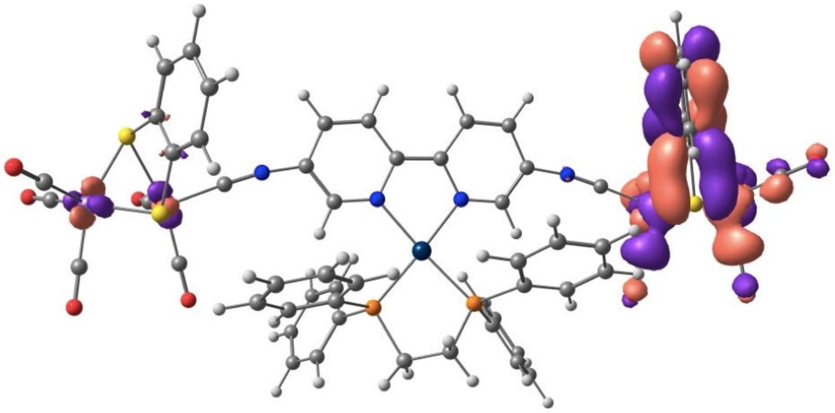

Figure S44. Computed HOMO of complex **10a** (SMD(CH_2Cl_2)-B3LYP-D3/def2-SVP).

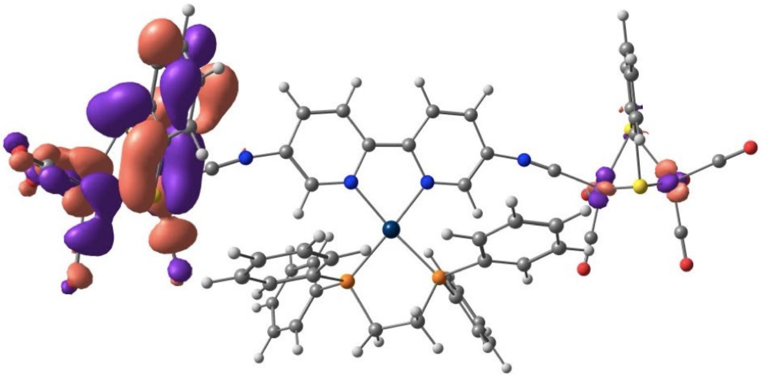

Figure S45. Computed HOMO-1 of complex **10a** (SMD(CH_2Cl_2)-B3LYP-D3/def2-SVP). HOMO and HOMO-1 of **10a** are degenerate orbitals (ΔE = 0.02 eV).

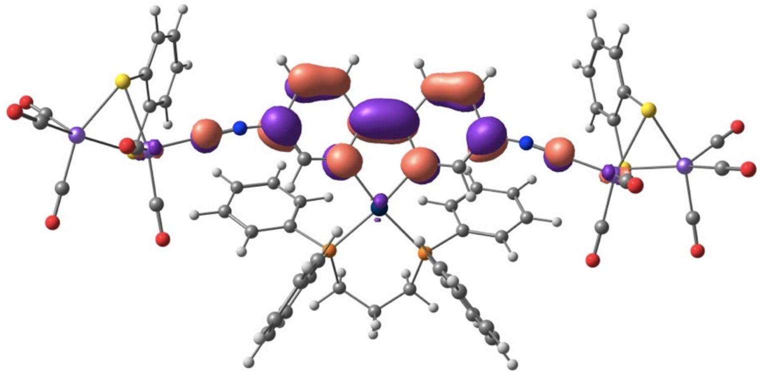

Figure S46. Computed LUMO of complex **10b** (SMD(CH_2Cl_2)-B3LYP-D3/def2-SVP).

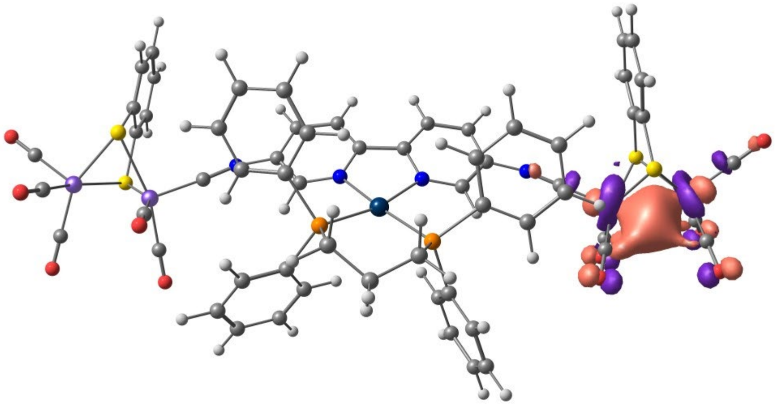

Figure S47. Computed HOMO of complex **10b** (SMD(CH_2Cl_2)-B3LYP-D3/def2-SVP).

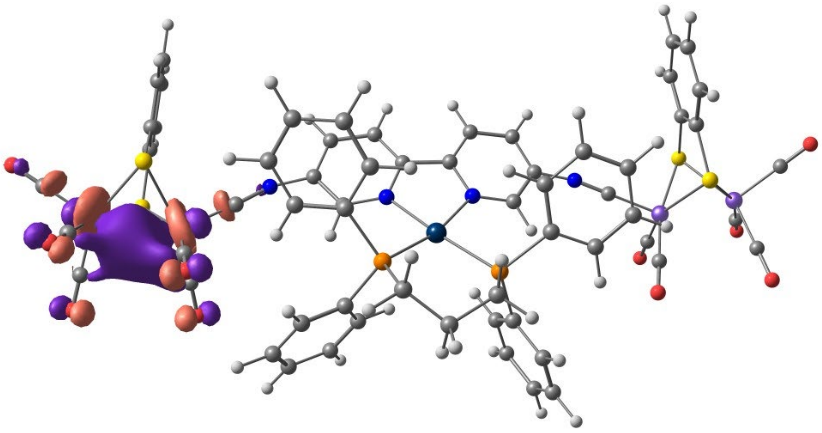

Figure S48. Computed HOMO-1 of complex **10b** (SMD(CH_2Cl_2)-B3LYP-D3/def2-SVP). HOMO and HOMO-1 of **10b** are degenerate orbitals (ΔE = 0.04 eV).

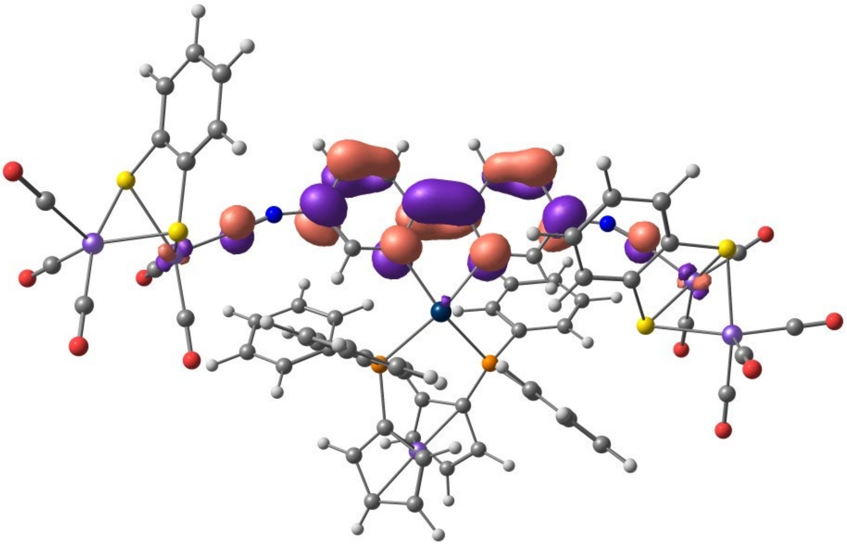

Figure S49. Computed LUMO of complex **10c** (SMD(CH_2Cl_2)-B3LYP-D3/def2-SVP).

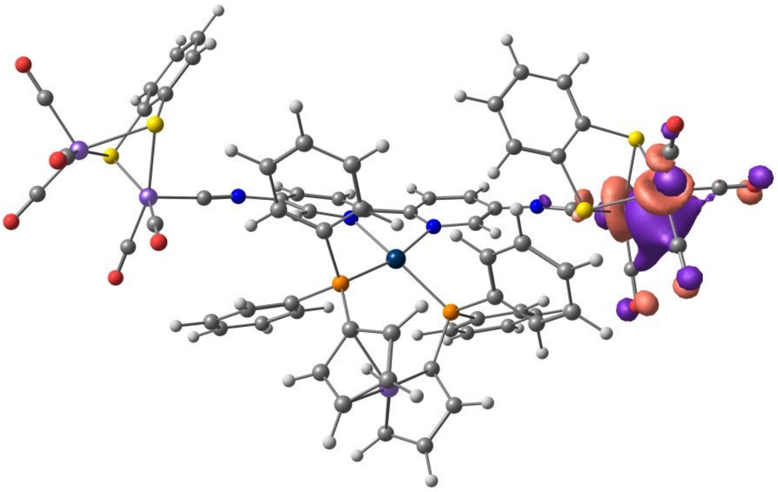

Figure S50. Computed HOMO of complex **10c** (SMD(CH_2Cl_2)-B3LYP-D3/def2-SVP).

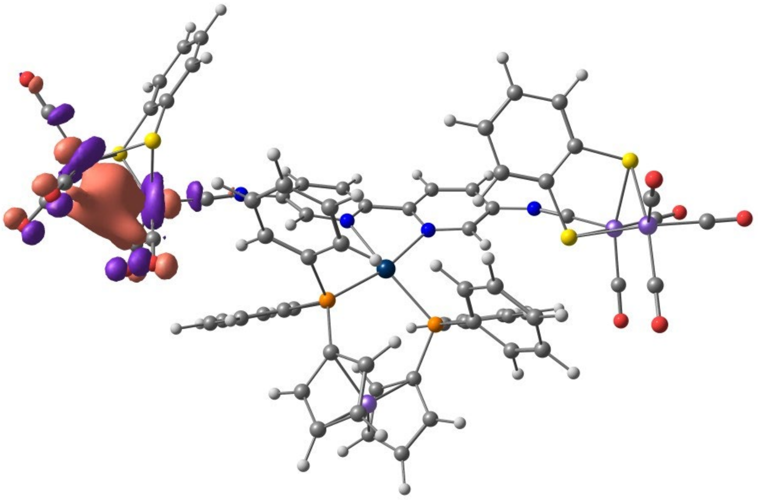

Figure S51. Computed HOMO-1 of complex **10c** (SMD(CH_2Cl_2)-B3LYP-D3/def2-SVP).
HOMO and HOMO-1 of **10c** are degenerate orbitals (ΔE = 0.04 eV).

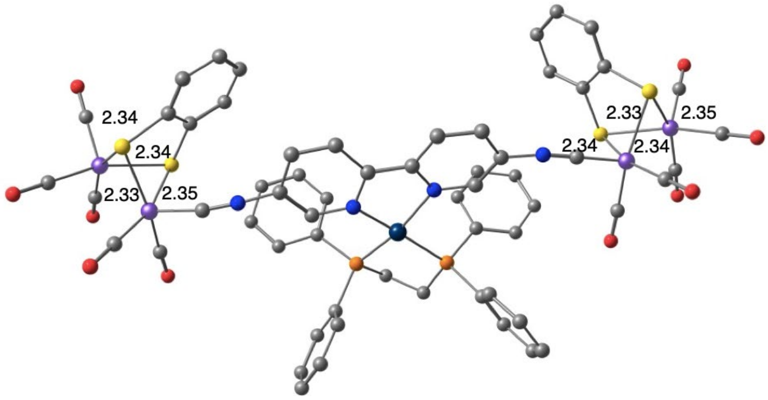

Figure S52. Computed structures of complex **10a** (SMD(CH_2Cl_2)-B3LYP-D3/def2-SVP), after the two successive 1e- reductions of the bipyridine Pt(II) fragment (**10a**^{0}/**10a**$^{2-}$). Distances in Å. H atoms omitted for clarity.

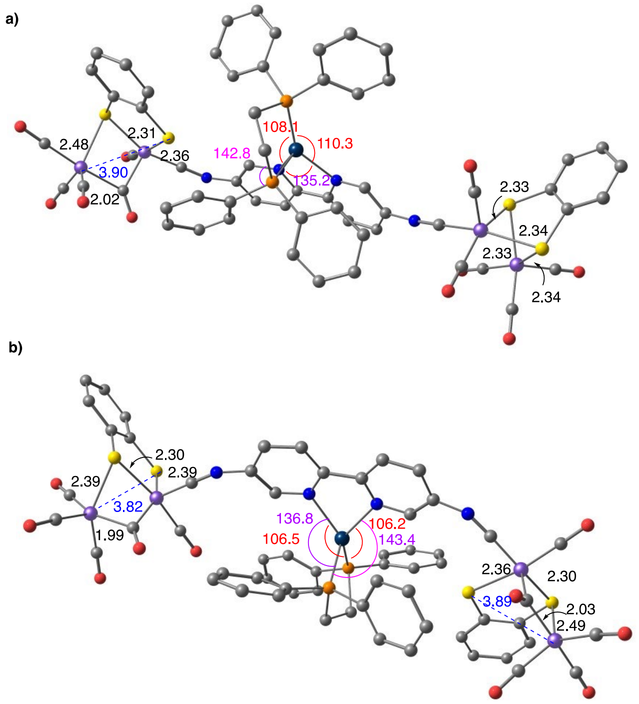

Figure S53. Changes of the structure of the Pt(II) complex during the reduction of the [FeS_2] moieties in species **10a^{2-}**: (a) **10a^{4-}**; (b) **10a^{6-}**). Computed at the (SMD(CH_2Cl_2)-B3LYP-D3/def2-SVP) level. Distances in Å.

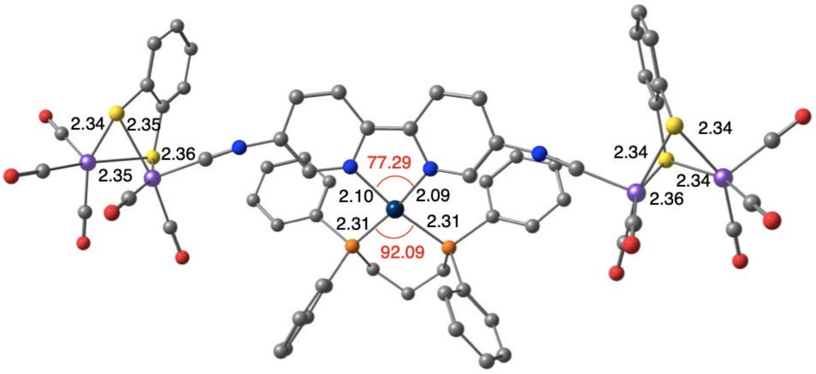

Figure S54. Computed structures of complex **10b** (SMD(CH_2Cl_2)-B3LYP-D3/def2-SVP), after the two successive 1e- reductions of the bipyridine Pt(II) fragment (**10b^0**/**10b^{2-}**). Distances in Å. H atoms omitted for clarity.

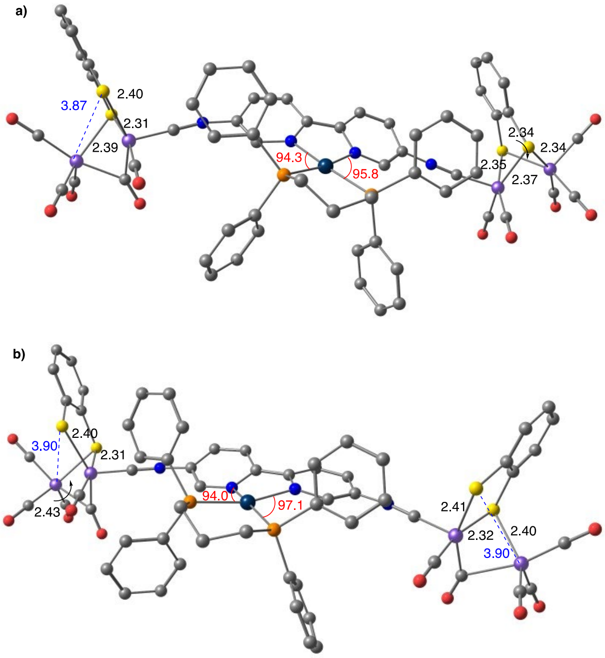

Figure S55. Changes of the structure of the Pt(II) complex during the reduction of the [FeS_2] moieties in species **10b^{2-}**: (a) **10b^{4-}**; (b) **10b^{6-}**. Computed at the (SMD(CH_2Cl_2)-B3LYP-D3/def2-SVP), level. Distances in Å.

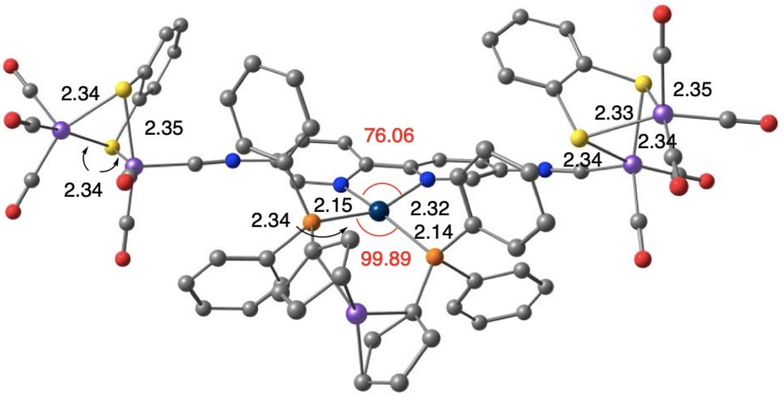

Figure S56. Computed structures of complex **10c** (SMD(CH_2Cl_2)-B3LYP-D3/def2-SVP), after the two successive 1e- reductions of the bipyridine Pt(II) fragment (**10c**^{0}/**10c^{2-}**). Distances in Å. H atoms omitted for clarity.

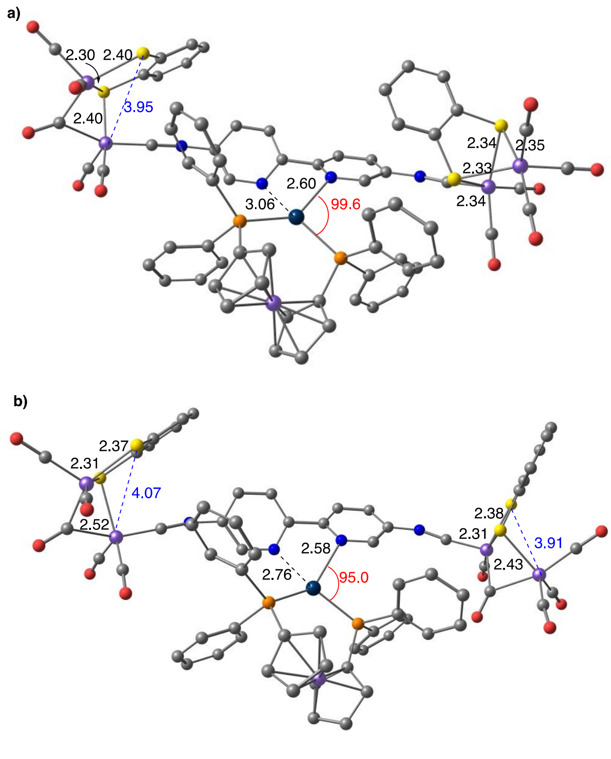

Figure S57. Changes of the structure of the Pt(II) complex during the reduction of the [FeS_2] moieties in species **10c**$^{2-}$: (a) **10c**$^{4-}$; (b) **10c**$^{6-}$. Computed at the (SMD(CH_2Cl_2)-B3LYP-D3/def2-SVP) level. Distances in Å.

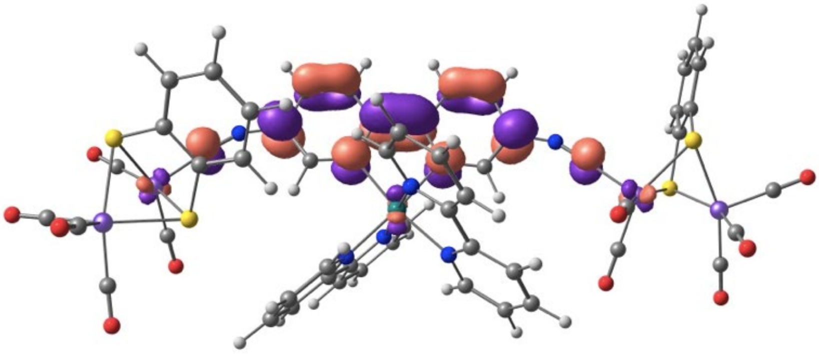

Figure S58. Computed LUMO of complex **12** (SMD(CH_3CN)-B3LYP-D3/def2-SVP).

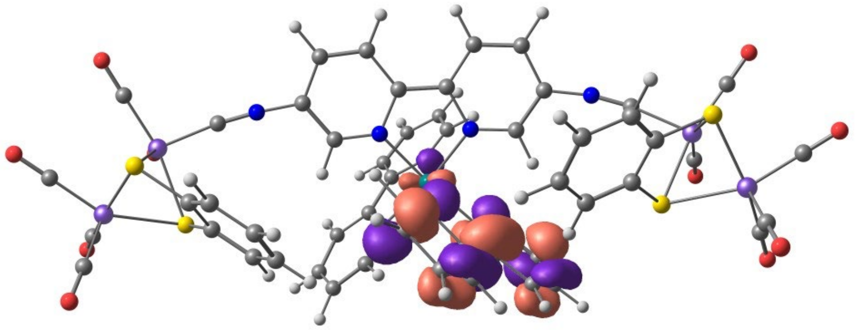

Figure S59. Computed LUMO of complex **12**$^{2-}$ (SMD(CH_3CN)-B3LYP-D3/def2-SVP).

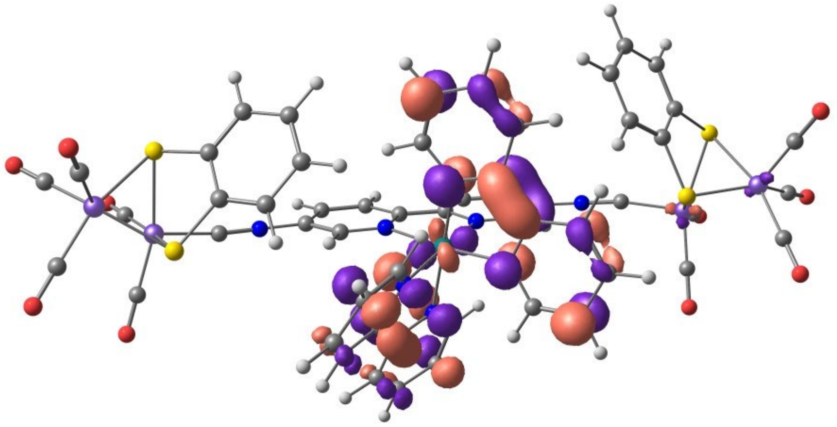

Figure S60. Computed HOMO of complex **12**$^{4-}$ (SMD(CH_3CN)-B3LYP-D3/def2-SVP).

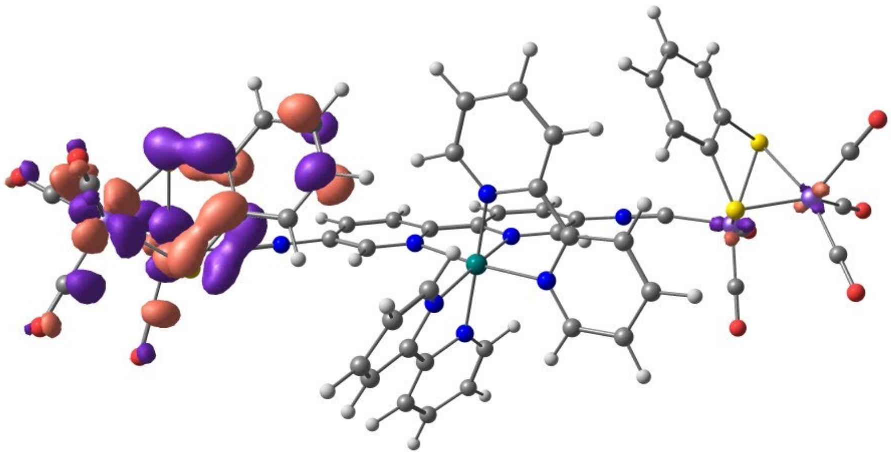

Figure S61. Computed LUMO of complex **12**$^{4-}$ (SMD(CH_3CN)-B3LYP-D3/def2-SVP).

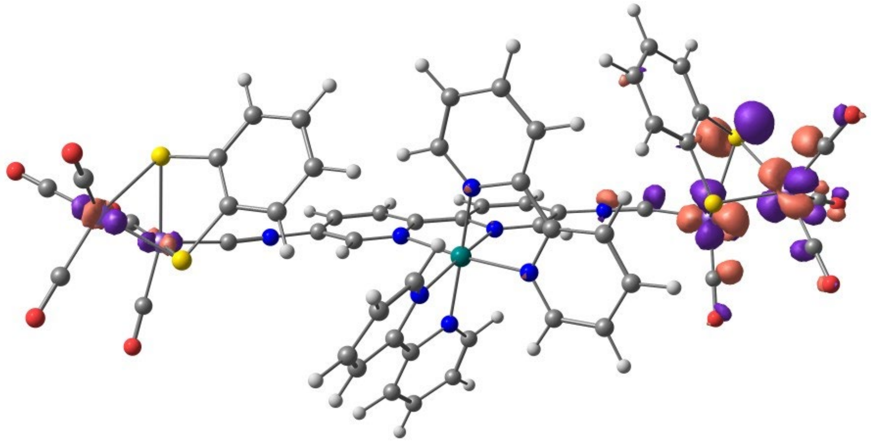

Figure S62. Computed LUMO+1 of complex **12**$^{4-}$ (SMD(CH_3CN)-B3LYP-D3/def2-SVP). LUMO and LUMO+1 of a**12**$^{4-}$ are degenerate orbitals (DE = 0.07 eV).

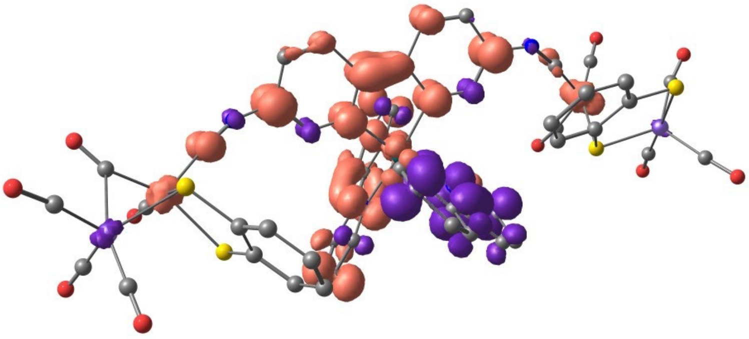

Figure S63. Computed Spin density of complex **12**$^{7-}$ (SMD(CH_3CN)-B3LYP-D3/def2-SVP). H atoms omitted for clarity.

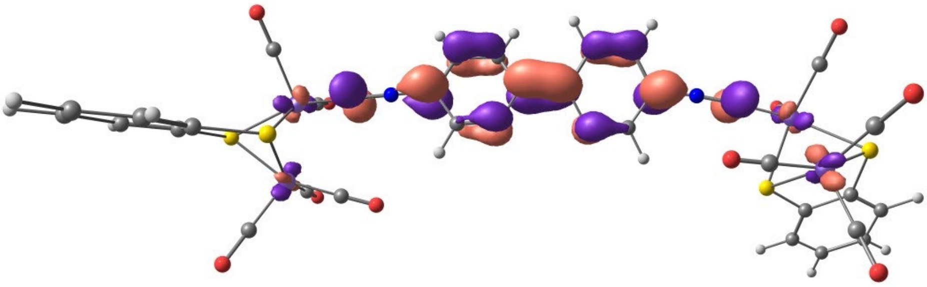

Figure S64. Computed LUMO of complex **6** (basal position) (SMD(CH_2Cl_2)-B3LYP-D3/def2-SVP).

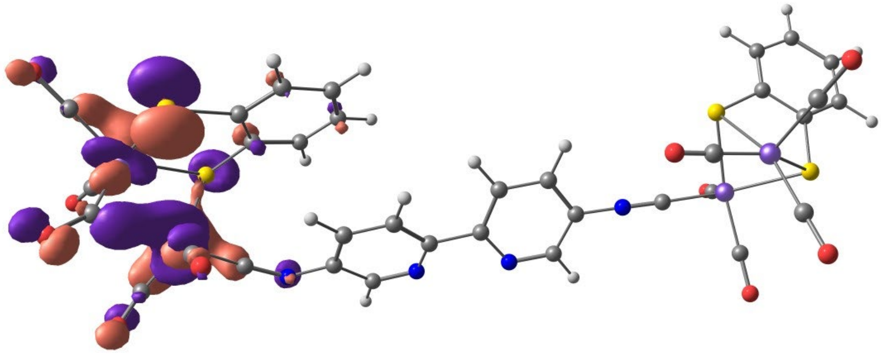

Figure S65. Computed HOMO of complex **6**$^{2-}$ (basal position) (SMD(CH_2Cl_2)-B3LYP-D3/def2-SVP).

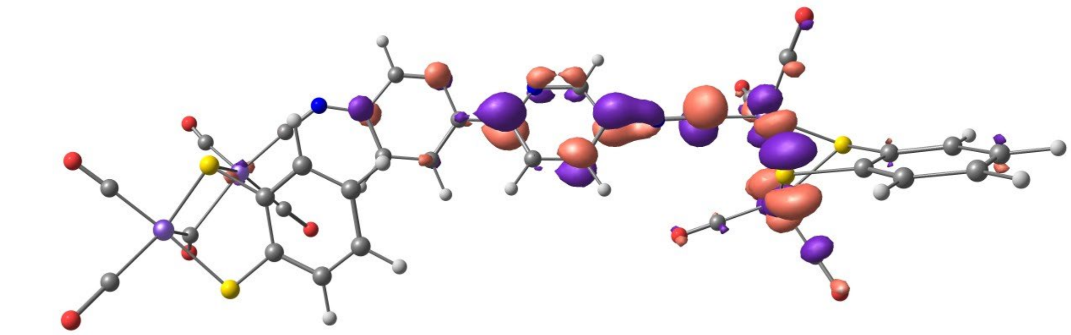

Figure S66. Computed LUMO of complex **6**$^{2-}$ (basal position) (SMD(CH_2Cl_2)-B3LYP-D3/def2-SVP).

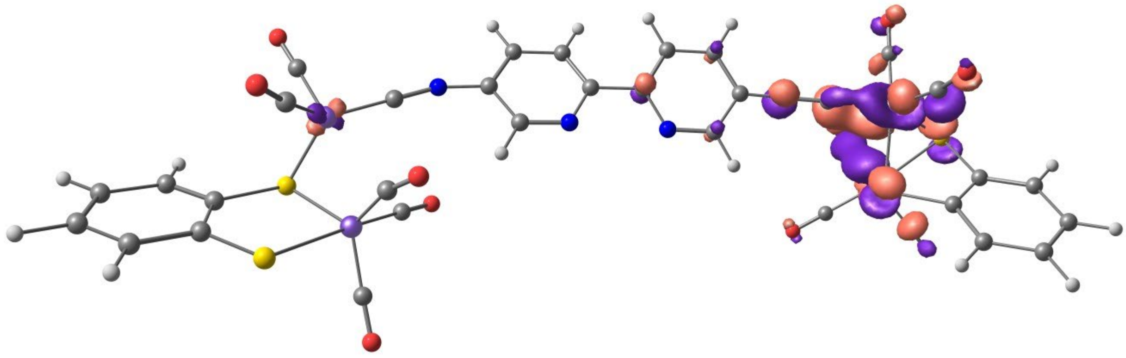

Figure S67. Computed HOMO of complex **6**$^{4-}$ (basal position) (SMD(CH_2Cl_2)-B3LYP-D3/def2-SVP).

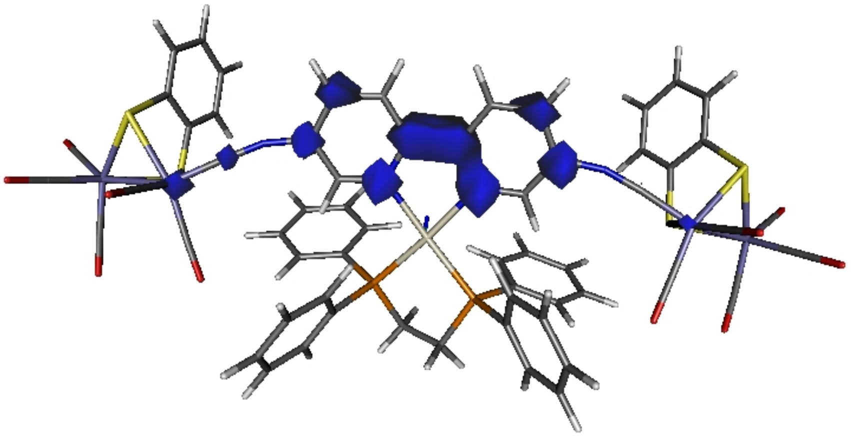

Figure S68. Computed Spin density of complex **10a**$^{\cdot-}$ (SMD(CH_2Cl_2)-B3LYP-D3/def2-SVP).

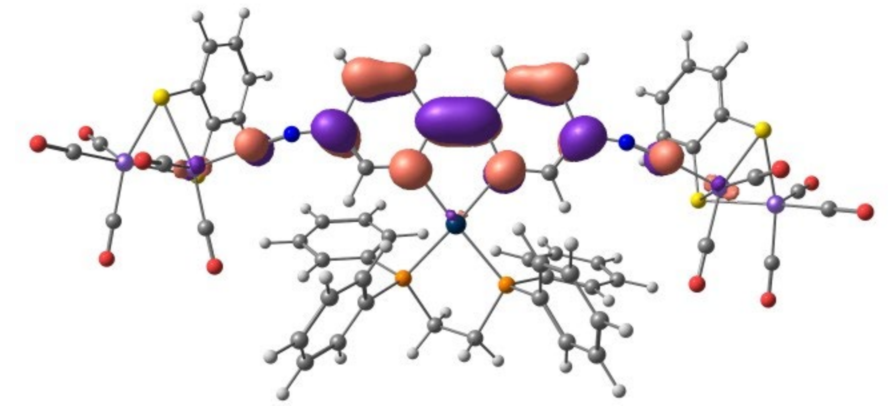

Figure S69. Computed LUMO of complex **10a**$^{-\cdot}$ (SMD(CH_2Cl_2)-B3LYP-D3/def2-SVP).

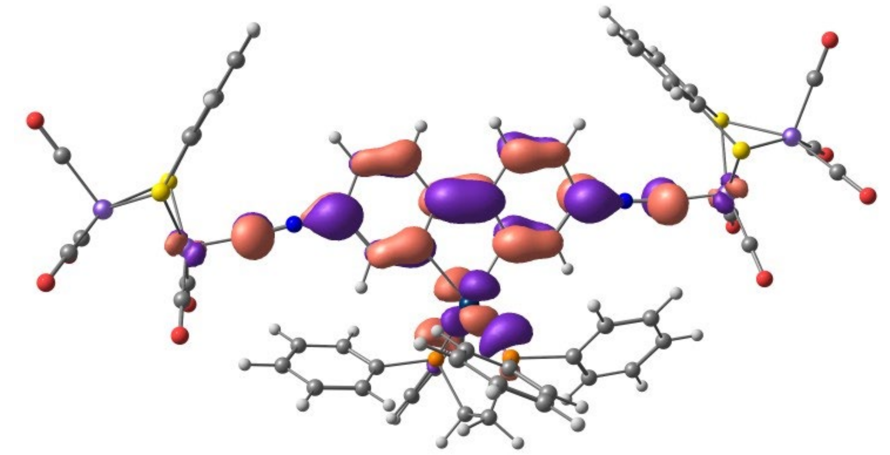

Figure S70. Computed SOMO of complex **10a**$^{3-\cdot}$ (SMD(CH_2Cl_2)-B3LYP-D3/def2-SVP).

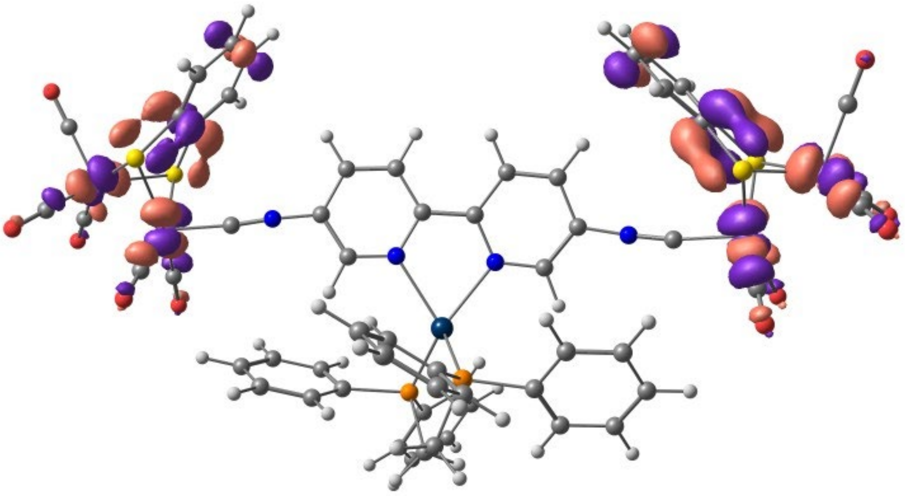

Figure S71. Computed LUMO of complex **10a**$^{3-\cdot}$ (SMD(CH_2Cl_2)-B3LYP-D3/def2-SVP).

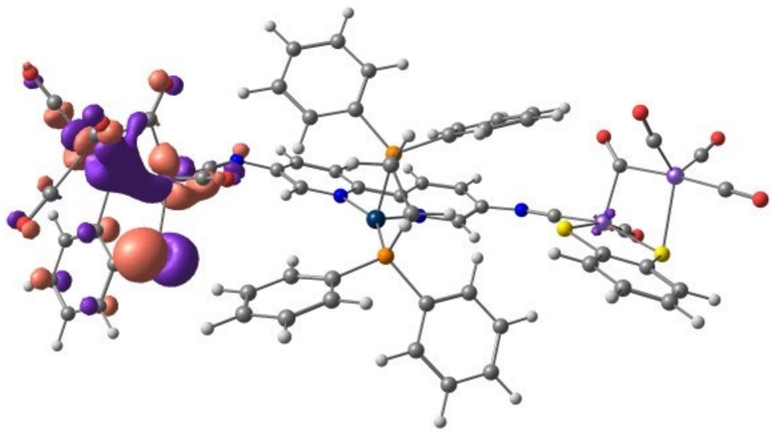

Figure S72. Computed SOMO of complex **10a**$^{5-\cdot}$ (SMD(CH_2Cl_2)-B3LYP-D3/def2-SVP).

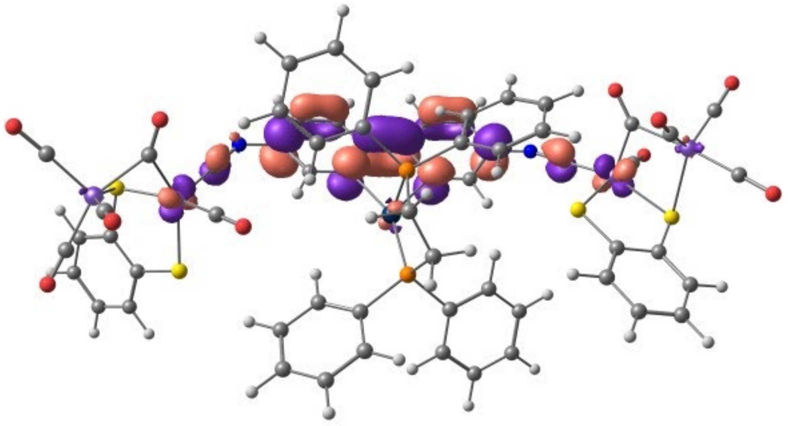

Figure S73. Computed LUMO of complex **10a**$^{5-\cdot}$ (SMD(CH_2Cl_2)-B3LYP-D3/def2-SVP).

Computational details.

Cartesian coordinates and energies (in Hartree) of compounds **6**, **10a**-**10c** and **12**, and the corresponding reduced species.

Compound 6

Sum of electronic and zero-point Energies = -8920.320018

Sum of electronic and thermal Energies = -8920.263859

Sum of electronic and thermal Enthalpies = -8920.262915

Sum of electronic and thermal Free Energies = -8920.419220

26 9.435430000 0.705218000 -0.089344000

26	7.806490000	-1.072351000	0.368017000
16	7.539424000	1.092002000	1.228707000
16	7.786965000	0.124140000	-1.648184000
6	9.803496000	2.380314000	-0.624174000
8	10.036175000	3.448625000	-0.966939000
6	10.421107000	0.584566000	1.395379000
8	11.045818000	0.514149000	2.355322000
6	10.641720000	-0.225591000	-1.021674000
8	11.411981000	-0.821970000	-1.628038000
6	6.014643000	-1.487008000	0.353904000
7	4.845569000	-1.598324000	0.303641000
6	8.513425000	-2.493480000	-0.451064000
8	8.970783000	-3.401704000	-0.982897000
6	8.286425000	-1.682866000	1.975890000
8	8.588072000	-2.064829000	3.015191000
6	6.519149000	1.348462000	-1.300364000
6	5.658455000	1.835549000	-2.283308000
6	6.407346000	1.797559000	0.026239000
6	4.679971000	2.781342000	-1.935676000
6	5.436539000	2.736925000	0.372053000
6	4.570210000	3.229257000	-0.618113000
1	5.746527000	1.482419000	-3.313537000
1	4.004334000	3.164322000	-2.704417000
1	5.350999000	3.082926000	1.404951000
1	3.808458000	3.965237000	-0.349334000
6	3.478922000	-1.505110000	0.202097000
6	2.877643000	-0.285729000	-0.143338000
6	2.659686000	-2.623618000	0.442854000
6	1.491146000	-0.240840000	-0.224261000
7	1.337408000	-2.568534000	0.359310000
6	0.744976000	-1.406005000	0.038258000
1	3.492227000	0.592895000	-0.348307000
1	3.119928000	-3.580953000	0.711840000
1	0.998234000	0.689177000	-0.510207000

6	-0.744961000	-1.405991000	-0.038461000
6	-1.491100000	-0.240756000	0.223836000
7	-1.337423000	-2.568566000	-0.359289000
6	-2.877599000	-0.285627000	0.142935000
6	-2.659704000	-2.623634000	-0.442807000
6	-3.478910000	-1.505062000	-0.202251000
1	-0.998162000	0.689305000	0.509595000
1	-3.492161000	0.593052000	0.347741000
1	-3.119974000	-3.581010000	-0.711602000
26	-7.806501000	-1.072372000	-0.367839000
26	-9.435446000	0.705185000	0.089518000
16	-7.786727000	0.124392000	1.648207000
16	-7.539648000	1.091873000	-1.228854000
6	-6.014641000	-1.486978000	-0.353908000
7	-4.845562000	-1.598273000	-0.303730000
6	-8.513253000	-2.493419000	0.451540000
8	-8.970486000	-3.401584000	0.983580000
6	-8.286645000	-1.683125000	-1.975557000
8	-8.588437000	-2.065240000	-3.014760000
6	-9.803527000	2.380320000	0.624224000
8	-10.036219000	3.448651000	0.966917000
6	-10.421321000	0.584339000	-1.395054000
8	-11.046165000	0.513799000	-2.354902000
6	-10.641558000	-0.225601000	1.022102000
8	-11.411707000	-0.821967000	1.628621000
6	-6.407420000	1.797634000	-0.026652000
6	-5.436696000	2.736984000	-0.372747000
6	-6.519012000	1.348723000	1.300031000
6	-4.570241000	3.229490000	0.617222000
6	-5.658197000	1.835988000	2.282779000
6	-4.679798000	2.781768000	1.934866000
1	-5.351324000	3.082834000	-1.405709000
1	-3.808554000	3.965458000	0.348224000
1	-5.746108000	1.483010000	3.313074000

1	-4.004066000	3.164888000	2.703453000

Compound 6^{2-}

Sum of electronic and zero-point Energies = -8920.515123

Sum of electronic and thermal Energies = -8920.458709

Sum of electronic and thermal Enthalpies = -8920.457765

Sum of electronic and thermal Free Energies = -8920.614667

26	-9.101941000	0.656006000	0.817647000
26	-7.961835000	-1.201782000	-0.699797000
16	-8.033756000	1.052678000	-1.182445000
16	-7.092511000	1.407958000	1.926473000
6	-9.868506000	2.098130000	1.480312000
8	-10.391158000	3.008337000	1.977179000
6	-10.536438000	0.047986000	-0.018271000
8	-11.519027000	-0.300959000	-0.524436000
6	-9.090898000	-0.674034000	1.984397000
8	-9.134829000	-1.425185000	2.871739000
6	-6.205803000	-1.354106000	-0.585975000
7	-5.003018000	-1.496543000	-0.599010000
6	-8.324018000	-2.694210000	0.132113000
8	-8.558708000	-3.698578000	0.674303000
6	-8.851930000	-1.620304000	-2.150404000
8	-9.325619000	-1.976081000	-3.159103000
6	-6.114395000	2.024867000	0.607815000
6	-4.895065000	2.692384000	0.875583000
6	-6.517271000	1.901079000	-0.747297000
6	-4.125669000	3.244870000	-0.148960000
6	-5.735050000	2.460135000	-1.769359000
6	-4.543266000	3.135386000	-1.482915000
1	-4.565943000	2.785838000	1.914630000
1	-3.194175000	3.764284000	0.095393000
1	-6.070356000	2.360124000	-2.805810000

1	-3.947261000	3.566086000	-2.292016000
6	-3.669868000	-1.393064000	-0.531990000
6	-2.991738000	-0.181242000	-0.817119000
6	-2.855443000	-2.507662000	-0.170689000
6	-1.611674000	-0.142428000	-0.718865000
7	-1.541072000	-2.456745000	-0.086582000
6	-0.887990000	-1.300417000	-0.346727000
1	-3.565636000	0.703138000	-1.094661000
1	-3.348088000	-3.462433000	0.052950000
1	-1.102261000	0.798264000	-0.931512000
6	0.584787000	-1.310434000	-0.219378000
6	1.368025000	-0.146007000	-0.422922000
7	1.175304000	-2.481841000	0.106990000
6	2.745676000	-0.203808000	-0.290744000
6	2.490941000	-2.547777000	0.233251000
6	3.332863000	-1.437115000	0.044096000
1	0.905560000	0.806449000	-0.679563000
1	3.369635000	0.679520000	-0.440665000
1	2.933168000	-3.516148000	0.497539000
26	7.680387000	-1.143884000	0.352869000
26	9.372652000	0.611128000	0.062396000
16	7.712597000	0.238692000	-1.545250000
16	7.485196000	0.939138000	1.409277000
6	5.863846000	-1.486285000	0.273267000
7	4.694877000	-1.546206000	0.185825000
6	8.342235000	-2.504122000	-0.592029000
8	8.769721000	-3.370833000	-1.212017000
6	8.091955000	-1.931239000	1.899956000
8	8.347679000	-2.433314000	2.900170000
6	9.809514000	2.320834000	-0.263253000
8	10.097187000	3.413118000	-0.459870000
6	10.332082000	0.260130000	1.523479000
8	10.938209000	0.034574000	2.472471000
6	10.546279000	-0.245497000	-0.974372000

8	11.292367000	-0.795248000	-1.651915000
6	6.381284000	1.790981000	0.277191000
6	5.433395000	2.717118000	0.710475000
6	6.485339000	1.466989000	-1.086016000
6	4.583507000	3.324265000	-0.228696000
6	5.642044000	2.069334000	-2.018815000
6	4.687283000	3.002936000	-1.583154000
1	5.351849000	2.963491000	1.771851000
1	3.837680000	4.048042000	0.108921000
1	5.721909000	1.812009000	-3.077706000
1	4.023269000	3.474341000	-2.311869000

Compound 6^{4-}

Sum of electronic and zero-point Energies = -8920.684918

Sum of electronic and thermal Energies = -8920.629310

Sum of electronic and thermal Enthalpies = -8920.628365

Sum of electronic and thermal Free Energies = -8920.780777

26	-9.312155000	0.037442000	0.803257000
26	-7.760847000	-0.832401000	-1.354952000
16	-8.085399000	1.354924000	-0.691946000
16	-7.450610000	0.328333000	2.377227000
6	-10.259267000	0.956762000	1.969591000
8	-10.897337000	1.503350000	2.775363000
6	-10.617466000	-0.169273000	-0.365991000
8	-11.508069000	-0.288256000	-1.100131000
6	-9.170400000	-1.703077000	1.126822000
8	-9.167564000	-2.803251000	1.511808000
6	-6.021760000	-0.886307000	-1.004181000
7	-4.828781000	-0.896385000	-0.836313000
6	-7.966570000	-2.561629000	-1.417360000
8	-8.105496000	-3.718488000	-1.463010000
6	-8.497178000	-0.548964000	-2.918465000

8	-8.864795000	-0.386163000	-4.019125000
6	-6.445519000	1.529111000	1.582059000
6	-5.331409000	2.077507000	2.262428000
6	-6.713338000	1.997052000	0.268650000
6	-4.534566000	3.066146000	1.684204000
6	-5.902872000	2.990313000	-0.302679000
6	-4.817870000	3.534267000	0.393627000
1	-5.105276000	1.718707000	3.270916000
1	-3.686227000	3.471070000	2.244105000
1	-6.132447000	3.337649000	-1.314452000
1	-4.198892000	4.308048000	-0.068372000
6	-3.499944000	-0.794061000	-0.633042000
6	-2.884791000	0.441080000	-0.325916000
6	-2.648743000	-1.928456000	-0.710193000
6	-1.512686000	0.479640000	-0.128695000
7	-1.339428000	-1.878691000	-0.520120000
6	-0.743668000	-0.701684000	-0.233822000
1	-3.497196000	1.338521000	-0.232079000
1	-3.094333000	-2.903711000	-0.943638000
1	-1.044078000	1.429595000	0.133784000
6	0.727236000	-0.702805000	-0.028913000
6	1.496376000	0.480155000	-0.114899000
7	1.321217000	-1.882665000	0.248345000
6	2.865801000	0.441352000	0.101696000
6	2.628057000	-1.932620000	0.455708000
6	3.476545000	-0.795422000	0.409422000
1	1.029440000	1.432022000	-0.373309000
1	3.479190000	1.340216000	0.026889000
1	3.072291000	-2.910238000	0.681496000
26	7.682144000	-0.789711000	1.404700000
26	9.355095000	-0.005513000	-0.687377000
16	7.591244000	0.308636000	-2.375383000
16	8.086762000	1.384666000	0.706375000
6	5.976701000	-0.863558000	0.903566000

7	4.801825000	-0.896828000	0.641995000
6	7.890158000	-2.511045000	1.570771000
8	8.036159000	-3.662325000	1.684182000
6	8.284321000	-0.445138000	3.011163000
8	8.572188000	-0.233763000	4.127294000
6	10.426578000	0.807098000	-1.821189000
8	11.155202000	1.275641000	-2.599825000
6	10.582333000	-0.230942000	0.560523000
8	11.427741000	-0.362251000	1.344509000
6	9.124470000	-1.745919000	-0.959745000
8	9.073416000	-2.853070000	-1.321723000
6	6.766781000	2.017511000	-0.329973000
6	5.930590000	3.018043000	0.189635000
6	6.555938000	1.526620000	-1.645332000
6	4.873167000	3.545364000	-0.559556000
6	5.470831000	2.061709000	-2.381064000
6	4.646159000	3.055767000	-1.853185000
1	6.115469000	3.383131000	1.204193000
1	4.232715000	4.323642000	-0.135842000
1	5.287610000	1.686419000	-3.392112000
1	3.820268000	3.447758000	-2.454373000

Compound 10a

Sum of electronic and zero-point Energies = -10726.276369

Sum of electronic and thermal Energies = -10726.194110

Sum of electronic and thermal Enthalpies = -10726.193166

Sum of electronic and thermal Free Energies = -10726.403331

26	9.892795000	-1.062719000	-0.055365000
26	7.779433000	-0.196907000	-0.969026000
16	8.558438000	-2.355378000	-1.467276000
16	7.920895000	-1.050907000	1.199788000
6	10.783595000	-2.295647000	0.900913000

8	11.343426000	-3.074416000	1.525922000
6	11.071828000	-0.841895000	-1.385608000
8	11.826479000	-0.717052000	-2.238563000
6	10.393080000	0.471912000	0.717118000
8	10.701025000	1.458768000	1.213853000
6	6.036377000	-0.571153000	-1.331874000
7	4.915666000	-0.898272000	-1.504626000
6	7.565783000	1.457541000	-0.337150000
8	7.410443000	2.519240000	0.072331000
6	8.416801000	0.417581000	-2.523501000
8	8.828763000	0.812419000	-3.518576000
6	7.315208000	-2.721756000	0.945101000
6	6.572089000	-3.407420000	1.904889000
6	7.607334000	-3.322767000	-0.290217000
6	6.117246000	-4.706138000	1.623181000
6	7.155862000	-4.611533000	-0.570659000
6	6.406758000	-5.303462000	0.395471000
1	6.348332000	-2.939501000	2.866297000
1	5.538160000	-5.248092000	2.374968000
1	7.387172000	-5.077577000	-1.531487000
1	6.055822000	-6.315783000	0.180621000
6	3.570630000	-1.130745000	-1.515138000
6	3.012740000	-2.323462000	-1.993353000
6	2.720078000	-0.143320000	-0.997012000
6	1.630275000	-2.467578000	-1.948168000
7	1.398993000	-0.308196000	-0.931456000
6	0.830541000	-1.446163000	-1.420148000
1	3.653012000	-3.114963000	-2.386028000
1	3.153247000	0.780612000	-0.622637000
1	1.176298000	-3.386726000	-2.314810000
6	-0.642078000	-1.504600000	-1.374387000
6	-1.396971000	-2.542612000	-1.936073000
7	-1.261975000	-0.446371000	-0.781641000
6	-2.786790000	-2.494164000	-1.903714000

6	-2.592241000	-0.366258000	-0.779256000
6	-3.400001000	-1.376250000	-1.323446000
1	-0.903018000	-3.387183000	-2.414828000
1	-3.390613000	-3.296542000	-2.330387000
1	-3.063080000	0.505830000	-0.331474000
26	-7.574854000	-0.260932000	-0.923627000
26	-9.936516000	-0.713228000	-0.399947000
16	-8.595776000	-2.305037000	-1.459554000
16	-8.254057000	-0.910365000	1.213450000
6	-5.866091000	-0.853419000	-1.096827000
7	-4.755339000	-1.221616000	-1.261042000
6	-7.901995000	0.369529000	-2.563012000
8	-8.106458000	0.776578000	-3.616238000
6	-7.156670000	1.360488000	-0.308145000
8	-6.852632000	2.390640000	0.097766000
6	-11.175795000	-1.699890000	0.448661000
8	-11.958570000	-2.316941000	1.011145000
6	-10.288105000	0.941423000	0.179417000
8	-10.503324000	2.003172000	0.555673000
6	-10.830692000	-0.431169000	-1.928456000
8	-11.403992000	-0.265239000	-2.906112000
6	-7.930631000	-2.674296000	1.170365000
6	-7.549562000	-3.396438000	2.300165000
6	-8.078321000	-3.317541000	-0.068802000
6	-7.307383000	-4.775136000	2.184990000
6	-7.838941000	-4.686172000	-0.183891000
6	-7.449920000	-5.414424000	0.952533000
1	-7.448325000	-2.895591000	3.265969000
1	-7.011949000	-5.345764000	3.068918000
1	-7.958515000	-5.186316000	-1.147965000
1	-7.264243000	-6.487914000	0.866460000
78	0.048178000	0.970252000	0.163410000
15	1.564838000	2.445081000	1.076194000
15	-1.452917000	2.109002000	1.472256000

6	-0.508361000	2.779734000	2.903296000
1	-1.144650000	3.445540000	3.504902000
1	-0.257306000	1.906599000	3.525742000
6	0.740568000	3.485536000	2.379762000
1	0.479838000	4.439895000	1.897093000
1	1.461481000	3.691291000	3.183705000
6	-2.225360000	3.479574000	0.564705000
6	-2.343357000	3.375422000	-0.833381000
6	-2.739006000	4.605713000	1.230455000
6	-2.978566000	4.387424000	-1.556448000
6	-3.368397000	5.615901000	0.498173000
6	-3.490983000	5.506897000	-0.891657000
1	-1.934497000	2.504042000	-1.351813000
1	-2.658413000	4.700990000	2.315501000
1	-3.068862000	4.303778000	-2.642123000
1	-3.766647000	6.491411000	1.016455000
1	-3.985373000	6.299487000	-1.458778000
6	2.245304000	3.605354000	-0.150509000
6	2.948651000	4.748409000	0.273240000
6	2.045467000	3.378043000	-1.523258000
6	3.462633000	5.639382000	-0.671381000
6	2.561618000	4.275866000	-2.461746000
6	3.273181000	5.402637000	-2.037993000
1	3.095921000	4.946690000	1.337715000
1	1.482709000	2.504798000	-1.859587000
1	4.011676000	6.523343000	-0.338153000
1	2.402723000	4.094597000	-3.527537000
1	3.676068000	6.103184000	-2.773709000
6	2.934245000	1.528222000	1.866465000
6	4.273630000	1.942750000	1.774399000
6	2.614527000	0.344830000	2.557696000
6	5.277908000	1.180695000	2.377975000
6	3.624349000	-0.407273000	3.161678000
6	4.957036000	0.009288000	3.070281000

1	4.545605000	2.844405000	1.223368000
1	1.578243000	0.001311000	2.606181000
1	6.318063000	1.499745000	2.298760000
1	3.371305000	-1.326070000	3.696340000
1	5.752011000	-0.584456000	3.526554000
6	-2.768559000	1.034329000	2.122083000
6	-2.399935000	-0.225651000	2.627503000
6	-4.124037000	1.395820000	2.054634000
6	-3.383298000	-1.117917000	3.056947000
6	-5.101491000	0.496583000	2.489972000
6	-4.734949000	-0.759392000	2.982470000
1	-1.346723000	-0.516951000	2.661798000
1	-4.424518000	2.363747000	1.649177000
1	-3.095802000	-2.099614000	3.440903000
1	-6.154986000	0.773887000	2.432628000
1	-5.505009000	-1.465241000	3.300863000

Compound 10a^{2-}

Sum of electronic and zero-point Energies = -10726.541528

Sum of electronic and thermal Energies = -10726.457664

Sum of electronic and thermal Enthalpies = -10726.456719

Sum of electronic and thermal Free Energies = -10726.673926

26	9.853756000	-0.837088000	0.117843000
26	7.731173000	-0.244259000	-0.964826000
16	8.773059000	-2.308495000	-1.352913000
16	7.813802000	-1.051049000	1.247267000
6	10.854686000	-1.962854000	1.092836000
8	11.496826000	-2.672755000	1.724892000
6	11.002723000	-0.383989000	-1.170908000
8	11.738635000	-0.095765000	-2.004082000
6	10.099074000	0.717966000	0.949618000
8	10.240931000	1.718801000	1.496759000

6	5.978393000	-0.762672000	-1.357552000
7	4.856183000	-1.061058000	-1.536385000
6	7.354866000	1.398320000	-0.402343000
8	7.098675000	2.459944000	-0.039475000
6	8.263933000	0.352009000	-2.552792000
8	8.616671000	0.732300000	-3.578227000
6	7.445312000	-2.787419000	0.995593000
6	6.737739000	-3.548051000	1.925724000
6	7.886054000	-3.367374000	-0.205671000
6	6.470102000	-4.899229000	1.651752000
6	7.622173000	-4.709216000	-0.477304000
6	6.909630000	-5.475829000	0.459244000
1	6.393470000	-3.094559000	2.858125000
1	5.914680000	-5.496690000	2.379066000
1	7.966234000	-5.156904000	-1.412851000
1	6.700279000	-6.527274000	0.247023000
6	3.503325000	-1.244889000	-1.613331000
6	2.891321000	-2.361637000	-2.302849000
6	2.681573000	-0.322107000	-0.983929000
6	1.532842000	-2.457701000	-2.294928000
7	1.345676000	-0.400154000	-0.960851000
6	0.696911000	-1.487117000	-1.622802000
1	3.517194000	-3.094148000	-2.815962000
1	3.151656000	0.512215000	-0.465981000
1	1.048738000	-3.283958000	-2.818196000
6	-0.686568000	-1.504283000	-1.559037000
6	-1.558683000	-2.523954000	-2.099964000
7	-1.297992000	-0.395732000	-0.898882000
6	-2.915757000	-2.417859000	-2.048048000
6	-2.632243000	-0.292409000	-0.888554000
6	-3.487805000	-1.237412000	-1.433491000
1	-1.105230000	-3.406570000	-2.554284000
1	-3.570058000	-3.194474000	-2.448538000
1	-3.071772000	0.588417000	-0.424789000

26	-7.691027000	-0.107094000	-0.819136000
26	-9.958952000	-0.691488000	-0.083954000
16	-8.779400000	-2.041231000	-1.586504000
16	-8.087718000	-1.167786000	1.248810000
6	-5.946233000	-0.685677000	-1.155828000
7	-4.833935000	-1.016011000	-1.340877000
6	-8.061664000	0.750706000	-2.329619000
8	-8.311556000	1.309994000	-3.302134000
6	-7.236567000	1.398778000	0.006471000
8	-6.921367000	2.363461000	0.548812000
6	-11.102206000	-1.833291000	0.694741000
8	-11.830388000	-2.550978000	1.214813000
6	-10.187759000	0.824067000	0.821493000
8	-10.333987000	1.800019000	1.411438000
6	-10.984349000	-0.107477000	-1.423191000
8	-11.643378000	0.252757000	-2.291495000
6	-7.773027000	-2.879954000	0.821327000
6	-7.222792000	-3.789684000	1.723503000
6	-8.086709000	-3.281426000	-0.488136000
6	-6.982796000	-5.110762000	1.312120000
6	-7.849525000	-4.593473000	-0.896506000
6	-7.293970000	-5.509746000	0.011393000
1	-6.980736000	-3.474371000	2.741308000
1	-6.550402000	-5.824756000	2.017528000
1	-8.094194000	-4.902639000	-1.915676000
1	-7.106499000	-6.538205000	-0.307425000
78	0.041732000	0.909555000	0.026166000
15	1.591245000	2.378106000	0.883271000
15	-1.453565000	2.078621000	1.316743000
6	-0.484523000	2.899626000	2.664100000
1	-1.115505000	3.622314000	3.203163000
1	-0.209525000	2.100226000	3.369839000
6	0.751335000	3.559753000	2.054249000
1	0.472642000	4.443798000	1.459235000

1	1.465611000	3.880482000	2.826599000
6	-2.375662000	3.396050000	0.451774000
6	-2.351255000	3.440786000	-0.952382000
6	-3.117498000	4.355599000	1.165134000
6	-3.070196000	4.427143000	-1.634585000
6	-3.832057000	5.339436000	0.478051000
6	-3.810920000	5.374893000	-0.921533000
1	-1.767750000	2.704093000	-1.510040000
1	-3.148875000	4.331896000	2.257305000
1	-3.048634000	4.454182000	-2.726861000
1	-4.409296000	6.080086000	1.037175000
1	-4.372602000	6.145201000	-1.456283000
6	2.439842000	3.435252000	-0.340955000
6	3.144703000	4.580684000	0.073726000
6	2.386884000	3.110635000	-1.706604000
6	3.800507000	5.378344000	-0.866841000
6	3.046817000	3.912442000	-2.642972000
6	3.755606000	5.043064000	-2.225263000
1	3.189109000	4.850471000	1.131606000
1	1.828761000	2.232664000	-2.039065000
1	4.349768000	6.263981000	-0.537636000
1	3.002881000	3.651365000	-3.703264000
1	4.271095000	5.667869000	-2.959241000
6	2.888481000	1.525755000	1.862598000
6	4.253611000	1.844197000	1.771808000
6	2.477535000	0.474880000	2.702642000
6	5.190972000	1.125838000	2.519879000
6	3.418409000	-0.232577000	3.454836000
6	4.776801000	0.090600000	3.362842000
1	4.600031000	2.632440000	1.102173000
1	1.422839000	0.192867000	2.749979000
1	6.251629000	1.367118000	2.430891000
1	3.090264000	-1.049883000	4.101943000
1	5.515937000	-0.473435000	3.936885000

6	-2.676485000	1.000952000	2.148754000
6	-2.184721000	-0.149285000	2.792713000
6	-4.061485000	1.222124000	2.087314000
6	-3.068703000	-1.058588000	3.376270000
6	-4.941852000	0.304764000	2.669893000
6	-4.449488000	-0.834593000	3.311698000
1	-1.109739000	-0.347722000	2.814740000
1	-4.466646000	2.092295000	1.569119000
1	-2.678654000	-1.952610000	3.869053000
1	-6.018161000	0.473351000	2.606171000
1	-5.143383000	-1.554487000	3.752829000

Compound 10a^{4-}

Sum of electronic and zero-point Energies = -10726.695202

Sum of electronic and thermal Energies = -10726.611564

Sum of electronic and thermal Enthalpies = -10726.610619

Sum of electronic and thermal Free Energies = -10726.827614

26	-8.936188000	-1.477896000	0.807333000
26	-7.059743000	-1.203486000	-1.343347000
16	-8.710784000	0.338911000	-0.874508000
16	-5.560635000	0.209346000	-0.187954000
6	-8.049355000	-1.471560000	2.309819000
8	-7.517878000	-1.573514000	3.350022000
6	-10.239739000	-0.452475000	1.412909000
8	-11.081337000	0.244859000	1.812252000
6	-9.934136000	-2.856227000	0.399173000
8	-10.593440000	-3.815121000	0.262646000
6	-5.604563000	-1.867109000	-2.038069000
7	-4.561053000	-2.313434000	-2.463388000
6	-7.363438000	-2.505468000	0.038323000
8	-6.799346000	-3.520674000	0.292823000
6	-8.092207000	-2.030583000	-2.495219000

8	-8.752242000	-2.579890000	-3.275682000
6	-6.545807000	1.540559000	0.390222000
6	-5.979917000	2.558663000	1.190172000
6	-7.923516000	1.627540000	0.067504000
6	-6.741638000	3.640499000	1.630010000
6	-8.680244000	2.726265000	0.510582000
6	-8.099576000	3.732091000	1.286294000
1	-4.924099000	2.487189000	1.461245000
1	-6.275373000	4.416809000	2.243764000
1	-9.741749000	2.773148000	0.252278000
1	-8.703043000	4.577468000	1.628266000
6	-3.204208000	-2.201963000	-2.535087000
6	-2.386121000	-3.216992000	-3.092418000
6	-2.565359000	-1.064333000	-2.009119000
6	-1.010896000	-3.080975000	-3.017761000
7	-1.236961000	-0.936210000	-1.948216000
6	-0.436581000	-1.943072000	-2.404159000
1	-2.845551000	-4.099700000	-3.540449000
1	-3.167457000	-0.250105000	-1.600250000
1	-0.374039000	-3.871651000	-3.416374000
6	0.983326000	-1.793298000	-2.110864000
6	1.952794000	-2.791930000	-2.357393000
7	1.329257000	-0.633551000	-1.464791000
6	3.251217000	-2.646803000	-1.897040000
6	2.585804000	-0.483649000	-1.030549000
6	3.564932000	-1.462576000	-1.189783000
1	1.675353000	-3.701925000	-2.889609000
1	4.012220000	-3.411868000	-2.055130000
1	2.831131000	0.446652000	-0.516767000
26	7.353200000	-0.354665000	0.711434000
26	8.994178000	-1.933921000	-0.237808000
16	8.264352000	-0.039843000	-1.413793000
16	9.610688000	-0.270889000	1.292703000
6	5.784717000	-0.906959000	-0.088604000

7	4.803861000	-1.244128000	-0.639131000
6	6.939545000	-1.222562000	2.214174000
8	6.661832000	-1.788882000	3.174292000
6	6.826659000	1.304730000	1.118831000
8	6.483883000	2.368714000	1.378073000
6	10.531959000	-2.219802000	-1.125475000
8	11.505240000	-2.402147000	-1.702787000
6	9.132691000	-3.158102000	1.058962000
8	9.229531000	-3.929971000	1.902599000
6	7.816477000	-2.931557000	-1.141235000
8	7.042336000	-3.550863000	-1.720894000
6	10.223432000	1.000885000	0.184303000
6	11.266562000	1.859632000	0.527155000
6	9.602164000	1.105069000	-1.070854000
6	11.690446000	2.831041000	-0.394336000
6	10.022156000	2.069029000	-1.986296000
6	11.072751000	2.934932000	-1.641755000
1	11.746603000	1.774146000	1.504739000
1	12.507697000	3.505776000	-0.128605000
1	9.535252000	2.146441000	-2.961166000
1	11.404046000	3.691490000	-2.357042000
78	-0.321132000	0.701229000	-0.602881000
15	-0.592731000	0.161104000	1.599355000
15	-0.825125000	2.823156000	-0.120615000
6	-1.556770000	2.785774000	1.617142000
1	-1.551363000	3.792468000	2.062057000
1	-2.608409000	2.480749000	1.499141000
6	-0.797851000	1.784541000	2.495265000
1	0.223538000	2.144876000	2.694451000
1	-1.282687000	1.645851000	3.473151000
6	0.548618000	4.049176000	0.101272000
6	1.854880000	3.649465000	-0.223400000
6	0.353424000	5.324362000	0.662867000
6	2.944291000	4.495005000	0.014193000

6	1.438895000	6.174804000	0.893702000
6	2.737628000	5.759961000	0.573889000
1	2.007199000	2.659343000	-0.661235000
1	-0.654057000	5.661725000	0.920609000
1	3.956142000	4.161298000	-0.231213000
1	1.271274000	7.163510000	1.329244000
1	3.586451000	6.422411000	0.762796000
6	0.858485000	-0.633826000	2.383980000
6	0.835743000	-1.925765000	2.940590000
6	2.099494000	0.030522000	2.273017000
6	2.020220000	-2.532220000	3.372904000
6	3.276670000	-0.573448000	2.716806000
6	3.244669000	-1.862357000	3.265161000
1	-0.111630000	-2.462155000	3.030840000
1	2.140405000	1.024583000	1.819064000
1	1.984210000	-3.537802000	3.800964000
1	4.226115000	-0.039615000	2.624310000
1	4.170045000	-2.337369000	3.597838000
6	-2.063241000	-0.858415000	1.994318000
6	-2.264382000	-2.016184000	1.215445000
6	-3.071429000	-0.464968000	2.892353000
6	-3.441242000	-2.757286000	1.333565000
6	-4.257346000	-1.198089000	2.989927000
6	-4.447672000	-2.341584000	2.211254000
1	-1.506306000	-2.311274000	0.485357000
1	-2.956964000	0.434239000	3.500521000
1	-3.599615000	-3.633000000	0.699265000
1	-5.060647000	-0.863844000	3.649485000
1	-5.397695000	-2.874354000	2.248098000
6	-2.144708000	3.702204000	-1.058517000
6	-3.374681000	3.026247000	-1.181780000
6	-1.977499000	4.927183000	-1.727243000
6	-4.419653000	3.571822000	-1.927379000
6	-3.023431000	5.466966000	-2.485973000

6	-4.248038000	4.797341000	-2.584246000
1	-3.528595000	2.053577000	-0.705673000
1	-1.029003000	5.464637000	-1.664266000
1	-5.364618000	3.025874000	-1.988595000
1	-2.877898000	6.420363000	-3.001816000
1	-5.063070000	5.225200000	-3.173967000

Compound 10a^{6-}

Sum of electronic and zero-point Energies = -10726.880393

Sum of electronic and thermal Energies = -10726.797788

Sum of electronic and thermal Enthalpies = -10726.796844

Sum of electronic and thermal Free Energies = -10727.005679

26	9.004777000	-1.329746000	-0.923166000
26	7.164085000	-1.110881000	1.268984000
16	8.778106000	0.465749000	0.787777000
16	5.623301000	0.273801000	0.140226000
6	8.050833000	-1.420424000	-2.381201000
8	7.475284000	-1.585566000	-3.389792000
6	10.202233000	-0.229044000	-1.609435000
8	10.975415000	0.515586000	-2.060910000
6	10.101830000	-2.631817000	-0.522382000
8	10.824736000	-3.545032000	-0.388162000
6	5.715561000	-1.813637000	1.957301000
7	4.683876000	-2.272576000	2.385293000
6	7.530958000	-2.433881000	-0.067232000
8	7.043834000	-3.500805000	-0.272226000
6	8.203162000	-1.879369000	2.448201000
8	8.871215000	-2.388560000	3.250790000
6	6.569162000	1.639885000	-0.425100000
6	5.972057000	2.658823000	-1.201606000
6	7.946922000	1.757682000	-0.109390000
6	6.700455000	3.772397000	-1.618295000

6	8.669428000	2.888196000	-0.529408000
6	8.056447000	3.895978000	-1.276943000
1	4.916033000	2.568069000	-1.466453000
1	6.208392000	4.549517000	-2.210484000
1	9.730744000	2.958868000	-0.275389000
1	8.633429000	4.767203000	-1.599396000
6	3.327678000	-2.198389000	2.533329000
6	2.573149000	-3.245494000	3.110393000
6	2.631593000	-1.069571000	2.065865000
6	1.190489000	-3.140391000	3.124746000
7	1.297680000	-0.976029000	2.086544000
6	0.553493000	-2.006567000	2.576288000
1	3.079198000	-4.128015000	3.505443000
1	3.188775000	-0.234012000	1.635573000
1	0.599419000	-3.957718000	3.539374000
6	-0.895959000	-1.893043000	2.389024000
6	-1.813080000	-2.897931000	2.763066000
7	-1.329486000	-0.780690000	1.730579000
6	-3.150834000	-2.795555000	2.417101000
6	-2.620103000	-0.672764000	1.400300000
6	-3.578502000	-1.662159000	1.686282000
1	-1.470562000	-3.776098000	3.310832000
1	-3.868438000	-3.573637000	2.682270000
1	-2.929736000	0.229287000	0.870240000
26	-7.134139000	-0.239193000	-0.132182000
26	-9.164114000	-0.831766000	-1.626391000
16	-8.766729000	-1.708771000	0.570328000
16	-8.126175000	1.410531000	1.289745000
6	-5.792071000	-0.980847000	0.711251000
7	-4.862174000	-1.514577000	1.260059000
6	-7.211642000	-1.253279000	-1.718891000
8	-6.483194000	-1.825643000	-2.478685000
6	-6.118456000	0.969363000	-0.876595000
8	-5.417614000	1.765030000	-1.357234000

6	-10.622579000	0.021218000	-1.038836000
8	-11.580026000	0.563404000	-0.665787000
6	-8.798315000	0.347222000	-2.859576000
8	-8.555517000	1.114775000	-3.705215000
6	-9.731519000	-2.265015000	-2.476383000
8	-10.139013000	-3.151918000	-3.115837000
6	-9.410032000	0.537721000	2.099767000
6	-10.207714000	1.175671000	3.082031000
6	-9.694155000	-0.825776000	1.811335000
6	-11.223594000	0.499934000	3.755179000
6	-10.718629000	-1.498077000	2.499286000
6	-11.485744000	-0.849423000	3.469309000
1	-10.007612000	2.226712000	3.310647000
1	-11.814978000	1.027101000	4.510201000
1	-10.912499000	-2.547752000	2.258862000
1	-12.278956000	-1.387454000	3.995485000
78	0.275849000	0.593617000	0.740621000
15	0.512958000	-0.146650000	-1.421887000
15	0.680782000	2.674961000	0.087274000
6	1.381400000	2.502650000	-1.662022000
1	1.346417000	3.470126000	-2.186921000
1	2.439328000	2.214879000	-1.553721000
6	0.614205000	1.421001000	-2.432418000
1	-0.430156000	1.731683000	-2.594628000
1	1.051117000	1.237437000	-3.425672000
6	-0.716970000	3.847270000	-0.256144000
6	-2.027980000	3.390599000	-0.046789000
6	-0.530135000	5.121672000	-0.823161000
6	-3.129865000	4.178611000	-0.398401000
6	-1.627916000	5.919670000	-1.158660000
6	-2.930280000	5.447622000	-0.950846000
1	-2.177842000	2.398584000	0.387198000
1	0.480647000	5.496603000	-1.005798000
1	-4.140083000	3.787901000	-0.257420000

1	-1.467005000	6.910252000	-1.593211000
1	-3.788216000	6.066920000	-1.225983000
6	-0.920055000	-1.054205000	-2.105595000
6	-0.855595000	-2.366640000	-2.607794000
6	-2.187645000	-0.449446000	-1.955226000
6	-2.028521000	-3.054424000	-2.941726000
6	-3.352346000	-1.132072000	-2.303627000
6	-3.278547000	-2.443466000	-2.791015000
1	0.114182000	-2.855563000	-2.728428000
1	-2.260629000	0.560192000	-1.541961000
1	-1.960994000	-4.077295000	-3.323829000
1	-4.329839000	-0.663519000	-2.185690000
1	-4.204382000	-2.968819000	-3.033813000
6	2.020846000	-1.113964000	-1.816203000
6	2.307394000	-2.226446000	-0.998093000
6	2.982977000	-0.711122000	-2.760835000
6	3.514934000	-2.915720000	-1.128258000
6	4.197146000	-1.394472000	-2.876558000
6	4.469714000	-2.494672000	-2.060420000
1	1.587285000	-2.532817000	-0.234994000
1	2.804823000	0.155481000	-3.399873000
1	3.738092000	-3.752599000	-0.461419000
1	4.958086000	-1.050302000	-3.580207000
1	5.441859000	-2.985621000	-2.110870000
6	1.986068000	3.672970000	0.920967000
6	3.261500000	3.081158000	1.016658000
6	1.763060000	4.897353000	1.576161000
6	4.291179000	3.705713000	1.720619000
6	2.794412000	5.517749000	2.291311000
6	4.062281000	4.929891000	2.362620000
1	3.458502000	2.110518000	0.552572000
1	0.778900000	5.369404000	1.539176000
1	5.271001000	3.223695000	1.762362000
1	2.602646000	6.468860000	2.796521000

1	4.865909000	5.418827000	2.919773000

Compound 10b

Sum of electronic and zero-point Energies = -10765.536730

Sum of electronic and thermal Energies = -10765.453346

Sum of electronic and thermal Enthalpies = -10765.452402

Sum of electronic and thermal Free Energies = -10765.664939

26	-9.851315000	0.345766000	-0.408782000
26	-7.430577000	0.082582000	-0.747790000
16	-8.704754000	1.490692000	-2.101705000
16	-8.311874000	1.495075000	0.909004000
6	-11.289707000	1.421391000	-0.357600000
8	-12.200221000	2.114222000	-0.344601000
6	-10.349504000	-0.913047000	-1.580040000
8	-10.664752000	-1.720509000	-2.330190000
6	-10.258577000	-0.712781000	0.978668000
8	-10.525074000	-1.370619000	1.877924000
6	-5.807575000	0.895839000	-0.832185000
7	-4.775439000	1.475617000	-0.860779000
6	-7.203961000	-1.211273000	0.458450000
8	-7.080371000	-2.045900000	1.238898000
6	-7.219511000	-1.085140000	-2.084514000
8	-7.072061000	-1.833434000	-2.941292000
6	-8.227031000	3.081163000	0.074944000
6	-7.981015000	4.273138000	0.753967000
6	-8.419218000	3.080670000	-1.315788000
6	-7.931590000	5.476535000	0.031432000
6	-8.371893000	4.274481000	-2.033724000
6	-8.126288000	5.477094000	-1.350782000
1	-7.831527000	4.268253000	1.836396000
1	-7.743108000	6.414296000	0.559961000
1	-8.526343000	4.271896000	-3.115337000
1	-8.091344000	6.415597000	-1.909437000
6	-3.434458000	1.716615000	-0.723664000

6	-2.858636000	2.991496000	-0.770986000
6	-2.606278000	0.612760000	-0.481533000
6	-1.477625000	3.097415000	-0.615182000
7	-1.302064000	0.736445000	-0.257424000
6	-0.705469000	1.954459000	-0.369271000
1	-3.477306000	3.873634000	-0.943560000
1	-3.037474000	-0.383688000	-0.480845000
1	-1.004085000	4.075224000	-0.694736000
6	0.768157000	1.934430000	-0.337888000
6	1.563091000	3.074523000	-0.501964000
7	1.340280000	0.697910000	-0.249103000
6	2.942249000	2.952735000	-0.638141000
6	2.651150000	0.563492000	-0.438802000
6	3.495788000	1.666630000	-0.640841000
1	1.105133000	4.062402000	-0.536041000
1	3.578821000	3.829940000	-0.763618000
1	3.080429000	-0.434263000	-0.435586000
26	7.594244000	0.329385000	-1.170104000
26	10.011802000	0.556943000	-0.783599000
16	8.687753000	2.402020000	-1.315986000
16	8.489275000	0.431175000	0.984469000
6	5.917288000	0.995791000	-0.954761000
7	4.829069000	1.434388000	-0.820598000
6	7.710503000	0.128353000	-2.941736000
8	7.789826000	0.000637000	-4.079349000
6	7.169245000	-1.387576000	-0.940497000
8	6.870523000	-2.484807000	-0.780135000
6	11.370746000	1.289342000	0.134227000
8	12.229315000	1.747923000	0.736349000
6	10.325664000	-1.195825000	-0.622261000
8	10.514397000	-2.321515000	-0.510888000
6	10.745977000	0.596279000	-2.417015000
8	11.221408000	0.634014000	-3.458589000
6	8.250351000	2.165796000	1.376278000

6	7.981142000	2.613266000	2.668578000
6	8.333997000	3.076586000	0.310913000
6	7.788324000	3.986108000	2.894230000
6	8.142830000	4.439366000	0.533871000
6	7.867675000	4.891511000	1.835092000
1	7.927171000	1.901731000	3.495699000
1	7.579790000	4.341120000	3.906505000
1	8.210073000	5.147156000	-0.295876000
1	7.720622000	5.959547000	2.013856000
78	-0.020577000	-0.824951000	0.500430000
15	-1.761167000	-2.039285000	1.410410000
15	1.558550000	-2.368306000	1.174417000
6	1.102312000	-3.547644000	2.503729000
1	1.937693000	-4.259646000	2.576635000
1	1.114046000	-2.959261000	3.435284000
6	-1.420633000	-3.350728000	2.650171000
1	-2.361890000	-3.916904000	2.728134000
1	-1.264780000	-2.846803000	3.616754000
6	2.117775000	-3.349434000	-0.263482000
6	1.899818000	-2.866802000	-1.568013000
6	2.797945000	-4.568283000	-0.084676000
6	2.358106000	-3.590579000	-2.671603000
6	3.253824000	-5.286252000	-1.193246000
6	3.035661000	-4.799864000	-2.486376000
1	1.373881000	-1.922746000	-1.725902000
1	2.983271000	-4.966500000	0.914500000
1	2.183130000	-3.207613000	-3.679937000
1	3.782565000	-6.230561000	-1.043145000
1	3.392979000	-5.365195000	-3.350727000
6	-2.771240000	-2.819565000	0.102954000
6	-4.022213000	-3.386532000	0.415137000
6	-2.318723000	-2.829591000	-1.228935000
6	-4.825636000	-3.908276000	-0.600818000
6	-3.121412000	-3.369263000	-2.237599000

6	-4.379632000	-3.895147000	-1.927644000
1	-4.382942000	-3.404909000	1.445790000
1	-1.342556000	-2.407936000	-1.479726000
1	-5.806157000	-4.321958000	-0.354774000
1	-2.763736000	-3.371409000	-3.269851000
1	-5.015282000	-4.296227000	-2.720499000
6	-2.778647000	-0.809329000	2.319199000
6	-4.119593000	-0.524386000	2.021802000
6	-2.121465000	-0.069323000	3.320291000
6	-4.799863000	0.475823000	2.722907000
6	-2.804765000	0.927177000	4.019094000
6	-4.145438000	1.201719000	3.721397000
1	-4.637917000	-1.061293000	1.229964000
1	-1.070798000	-0.264501000	3.550898000
1	-5.842731000	0.688338000	2.477180000
1	-2.287140000	1.494447000	4.796468000
1	-4.677584000	1.985251000	4.266460000
6	3.010193000	-1.505419000	1.879575000
6	2.771934000	-0.433843000	2.758455000
6	4.327977000	-1.878759000	1.572542000
6	3.844639000	0.261072000	3.320557000
6	5.396602000	-1.181532000	2.144652000
6	5.158456000	-0.109592000	3.010185000
1	1.746956000	-0.131850000	2.989018000
1	4.530249000	-2.696786000	0.878868000
1	3.654497000	1.099178000	3.995419000
1	6.421471000	-1.468939000	1.904996000
1	5.998541000	0.443082000	3.436698000
6	-0.237956000	-4.263916000	2.327894000
1	-0.329919000	-4.695096000	1.316623000
1	-0.266860000	-5.112622000	3.028781000

Compound 10b^{2-}

Sum of electronic and zero-point Energies = -10765.801148
Sum of electronic and thermal Energies = -10765.716453
Sum of electronic and thermal Enthalpies = -10765.715509
Sum of electronic and thermal Free Energies = -10765.934440

26	-9.989845000	0.735230000	-0.939009000
26	-7.590692000	0.272115000	-1.169212000
16	-8.519407000	2.325213000	-1.824299000
16	-8.532665000	0.935568000	0.887589000
6	-11.340412000	1.762818000	-0.355650000
8	-12.208475000	2.412871000	0.018062000
6	-10.547233000	0.353331000	-2.589887000
8	-10.896858000	0.116176000	-3.657753000
6	-10.438912000	-0.891282000	-0.366812000
8	-10.720288000	-1.940107000	0.009735000
6	-5.854006000	0.909084000	-0.900993000
7	-4.762183000	1.301613000	-0.718120000
6	-7.324746000	-1.376040000	-0.566068000
8	-7.130334000	-2.438183000	-0.167814000
6	-7.465339000	-0.258402000	-2.859470000
8	-7.385790000	-0.597691000	-3.954679000
6	-8.166509000	2.690018000	0.869390000
6	-7.870426000	3.406212000	2.028634000
6	-8.158096000	3.331004000	-0.381082000
6	-7.562406000	4.773214000	1.936515000
6	-7.853863000	4.688818000	-0.472901000
6	-7.554261000	5.410000000	0.694550000
1	-7.877402000	2.904377000	2.999247000
1	-7.328991000	5.335630000	2.844067000
1	-7.845972000	5.184059000	-1.446944000
1	-7.313803000	6.473792000	0.623834000
6	-3.447312000	1.569803000	-0.460906000
6	-2.907837000	2.912242000	-0.382154000
6	-2.601809000	0.499603000	-0.207132000

6	-1.569666000	3.058544000	-0.171997000
7	-1.307446000	0.630339000	0.094738000
6	-0.693116000	1.916317000	-0.023397000
1	-3.570650000	3.771842000	-0.498618000
1	-3.018303000	-0.504662000	-0.243880000
1	-1.133844000	4.058559000	-0.130976000
6	0.687759000	1.917266000	-0.034179000
6	1.553313000	3.061984000	-0.218602000
7	1.310481000	0.634858000	0.095672000
6	2.890520000	2.922417000	-0.436691000
6	2.605482000	0.511176000	-0.208528000
6	3.441447000	1.583502000	-0.486223000
1	1.107136000	4.058147000	-0.208873000
1	3.542996000	3.784707000	-0.586525000
1	3.031452000	-0.489140000	-0.224999000
26	7.594169000	0.343413000	-1.250592000
26	9.998091000	0.768425000	-1.005522000
16	8.515166000	2.475094000	-1.600027000
16	8.576273000	0.682643000	0.864178000
6	5.855826000	0.958003000	-0.943458000
7	4.757333000	1.325830000	-0.749429000
6	7.549341000	0.027421000	-2.996477000
8	7.535274000	-0.182654000	-4.126574000
6	7.259240000	-1.356487000	-0.858133000
8	7.017214000	-2.447235000	-0.584026000
6	11.364422000	1.611417000	-0.207109000
8	12.244853000	2.126295000	0.318261000
6	10.358990000	-0.962559000	-0.792822000
8	10.593910000	-2.079285000	-0.650136000
6	10.622777000	0.754658000	-2.676506000
8	11.022492000	0.766258000	-3.752606000
6	8.230345000	2.422084000	1.125842000
6	7.977378000	2.954888000	2.389545000
6	8.197301000	3.247714000	-0.010647000

6	7.689019000	4.323351000	2.517555000
6	7.913169000	4.607161000	0.116345000
6	7.657768000	5.143798000	1.388855000
1	8.003781000	2.309659000	3.271076000
1	7.489258000	4.741959000	3.507135000
1	7.887688000	5.246635000	-0.769344000
1	7.432735000	6.208569000	1.489056000
78	-0.002764000	-0.860172000	0.766885000
15	-1.683781000	-2.274766000	1.496770000
15	1.648877000	-2.342887000	1.428343000
6	1.257794000	-3.514825000	2.795314000
1	2.127463000	-4.180852000	2.896483000
1	1.230759000	-2.898152000	3.708133000
6	-1.285410000	-3.446537000	2.861475000
1	-2.181205000	-4.066661000	3.014437000
1	-1.175917000	-2.825151000	3.765097000
6	2.228585000	-3.374465000	0.026743000
6	1.937670000	-2.967693000	-1.287830000
6	2.962361000	-4.557277000	0.231472000
6	2.375561000	-3.727568000	-2.376064000
6	3.396593000	-5.314681000	-0.859482000
6	3.104454000	-4.901857000	-2.164086000
1	1.365395000	-2.053525000	-1.461274000
1	3.207086000	-4.894681000	1.240490000
1	2.142304000	-3.400300000	-3.392311000
1	3.966468000	-6.231362000	-0.687953000
1	3.444631000	-5.497336000	-3.015279000
6	-2.355512000	-3.309313000	0.139674000
6	-3.253437000	-4.364044000	0.390535000
6	-1.963498000	-3.043093000	-1.183976000
6	-3.753135000	-5.129177000	-0.665948000
6	-2.463825000	-3.813493000	-2.237590000
6	-3.359881000	-4.855534000	-1.981112000
1	-3.577088000	-4.591735000	1.408303000

1	-1.262671000	-2.231099000	-1.390511000
1	-4.453447000	-5.942543000	-0.460041000
1	-2.149695000	-3.596575000	-3.261536000
1	-3.751614000	-5.457131000	-2.805410000
6	-3.074970000	-1.314897000	2.222159000
6	-4.421480000	-1.520797000	1.884086000
6	-2.735893000	-0.288808000	3.121835000
6	-5.414172000	-0.704772000	2.437151000
6	-3.731011000	0.522313000	3.671073000
6	-5.071975000	0.319329000	3.324339000
1	-4.707486000	-2.292322000	1.167598000
1	-1.687467000	-0.103384000	3.370473000
1	-6.458275000	-0.857717000	2.158793000
1	-3.455947000	1.326005000	4.358630000
1	-5.850260000	0.965848000	3.737457000
6	3.110580000	-1.466140000	2.113261000
6	2.864067000	-0.416787000	3.015782000
6	4.430391000	-1.768793000	1.745039000
6	3.926275000	0.324908000	3.536617000
6	5.490279000	-1.020467000	2.268211000
6	5.240887000	0.028229000	3.157993000
1	1.836943000	-0.160416000	3.288914000
1	4.640817000	-2.564955000	1.028606000
1	3.725417000	1.148345000	4.226511000
1	6.514392000	-1.245592000	1.965551000
1	6.070845000	0.622254000	3.547861000
6	-0.040361000	-4.308392000	2.645925000
1	-0.079282000	-4.817552000	1.667673000
1	-0.041544000	-5.105160000	3.407157000

Compound 10b^{4-}

Sum of electronic and zero-point Energies = -10765.982652

Sum of electronic and thermal Energies = -10765.898235

Sum of electronic and thermal Enthalpies = -10765.897291

Sum of electronic and thermal Free Energies = -10766.111430

26	-9.483661000	-0.524174000	2.100352000
26	-7.435842000	-0.501248000	0.525207000
16	-8.343482000	-2.477070000	1.307975000
16	-8.445702000	-0.987188000	-1.604101000
6	-11.002777000	-1.061197000	1.347750000
8	-12.017388000	-1.412392000	0.897553000
6	-9.668797000	-0.816625000	3.818469000
8	-9.848931000	-0.918223000	4.968869000
6	-9.773343000	1.166929000	1.806205000
8	-9.998560000	2.305956000	1.657665000
6	-5.768299000	-1.122188000	0.358797000
7	-4.659226000	-1.545053000	0.251591000
6	-7.044053000	1.116950000	0.023395000
8	-6.782312000	2.199133000	-0.325133000
6	-7.521022000	0.025764000	2.310292000
8	-6.870798000	0.513086000	3.188109000
6	-9.198476000	-2.546751000	-1.357788000
6	-9.874179000	-3.200459000	-2.419919000
6	-9.165005000	-3.210646000	-0.097362000
6	-10.480684000	-4.444552000	-2.253463000
6	-9.778993000	-4.465733000	0.055667000
6	-10.437626000	-5.089414000	-1.007400000
1	-9.913394000	-2.703292000	-3.394101000
1	-10.991285000	-4.914879000	-3.099774000
1	-9.740792000	-4.954497000	1.034147000
1	-10.912075000	-6.064579000	-0.865797000
6	-3.302158000	-1.684700000	0.113290000
6	-2.660181000	-2.979398000	0.058399000
6	-2.505990000	-0.562387000	-0.010349000
6	-1.299502000	-3.040967000	-0.022868000
7	-1.166746000	-0.604010000	-0.199391000

6	-0.489129000	-1.846549000	-0.071209000
1	-3.271259000	-3.883610000	0.101539000
1	-2.979457000	0.415180000	0.041698000
1	-0.798943000	-4.011367000	-0.031741000
6	0.889810000	-1.778152000	0.025667000
6	1.807469000	-2.877450000	0.201428000
7	1.443188000	-0.451848000	-0.020654000
6	3.125670000	-2.679912000	0.482202000
6	2.712463000	-0.271302000	0.355969000
6	3.596081000	-1.309366000	0.620350000
1	1.417590000	-3.894681000	0.122865000
1	3.822995000	-3.509131000	0.614151000
1	3.079039000	0.750395000	0.435602000
26	7.848052000	-0.422072000	1.366779000
26	10.101396000	-1.201931000	0.786384000
16	8.459697000	-2.682832000	1.550340000
16	8.486602000	-0.837181000	-0.878114000
6	6.014347000	-0.752764000	1.150864000
7	4.890908000	-1.013930000	0.927472000
6	7.951644000	-0.203920000	3.125574000
8	8.027160000	-0.070210000	4.264852000
6	7.829224000	1.328258000	1.076632000
8	7.804519000	2.461180000	0.876040000
6	11.207659000	-2.269143000	-0.137489000
8	11.925347000	-2.936302000	-0.734993000
6	10.735685000	0.430800000	0.473994000
8	11.138575000	1.486857000	0.261159000
6	10.875450000	-1.248715000	2.391757000
8	11.366805000	-1.285932000	3.429481000
6	7.817397000	-2.484308000	-1.107077000
6	7.292705000	-2.919947000	-2.323865000
6	7.796834000	-3.331405000	0.014056000
6	6.738501000	-4.206591000	-2.418191000
6	7.249257000	-4.610684000	-0.079769000

6	6.716280000	-5.046527000	-1.303719000
1	7.308341000	-2.261434000	-3.195132000
1	6.322867000	-4.545628000	-3.370336000
1	7.231445000	-5.263950000	0.796015000
1	6.282310000	-6.046847000	-1.377005000
78	0.083276000	0.973289000	-0.744829000
15	-1.630389000	2.268524000	-1.603606000
15	1.671252000	2.582513000	-1.248766000
6	1.310220000	3.760290000	-2.622317000
1	2.129463000	4.494751000	-2.635693000
1	1.408328000	3.167274000	-3.546197000
6	-1.204798000	3.500966000	-2.906598000
1	-2.129233000	4.055475000	-3.125778000
1	-0.971886000	2.912233000	-3.808399000
6	2.072442000	3.626721000	0.208377000
6	1.667560000	3.198405000	1.485212000
6	2.769614000	4.843100000	0.084886000
6	1.959263000	3.967398000	2.615428000
6	3.056680000	5.610377000	1.216973000
6	2.651837000	5.174770000	2.483725000
1	1.116011000	2.262097000	1.596935000
1	3.100873000	5.199261000	-0.892634000
1	1.636834000	3.621814000	3.600751000
1	3.598604000	6.553232000	1.107251000
1	2.875020000	5.777999000	3.367586000
6	-2.516223000	3.215273000	-0.307259000
6	-3.471542000	4.196290000	-0.632694000
6	-2.251003000	2.939511000	1.045368000
6	-4.153500000	4.876910000	0.378456000
6	-2.935566000	3.623799000	2.053628000
6	-3.888953000	4.590747000	1.722386000
1	-3.705856000	4.424931000	-1.674596000
1	-1.508268000	2.183875000	1.311423000
1	-4.902645000	5.627413000	0.114169000

1	-2.725039000	3.393649000	3.100857000
1	-4.430689000	5.119335000	2.510876000
6	-2.859094000	1.217914000	-2.476909000
6	-4.236994000	1.274047000	-2.222808000
6	-2.357937000	0.269879000	-3.386446000
6	-5.104505000	0.383281000	-2.863493000
6	-3.228994000	-0.613069000	-4.028528000
6	-4.602804000	-0.562456000	-3.761197000
1	-4.645685000	1.976125000	-1.496132000
1	-1.281500000	0.199554000	-3.566125000
1	-6.171039000	0.402053000	-2.632069000
1	-2.830870000	-1.357346000	-4.723126000
1	-5.285195000	-1.272116000	-4.236157000
6	3.241040000	1.831969000	-1.842883000
6	3.133598000	0.786503000	-2.776773000
6	4.507498000	2.224400000	-1.384181000
6	4.280915000	0.139643000	-3.241031000
6	5.653006000	1.570943000	-1.850617000
6	5.541838000	0.527722000	-2.773699000
1	2.146933000	0.457369000	-3.114181000
1	4.609674000	3.017175000	-0.640847000
1	4.188588000	-0.683437000	-3.954062000
1	6.634627000	1.868931000	-1.478566000
1	6.439780000	0.008662000	-3.115231000
6	-0.053405000	4.449779000	-2.572704000
1	-0.214364000	4.934907000	-1.594679000
1	-0.051691000	5.260691000	-3.319173000

Compound 10b^{6-}

Sum of electronic and zero-point Energies = -10766.141225

Sum of electronic and thermal Energies = -10766.057549

Sum of electronic and thermal Enthalpies = -10766.056605

Sum of electronic and thermal Free Energies = -10766.268665

26	9.954219000	-0.425114000	-2.063056000
26	7.664152000	-0.284462000	-0.607536000
16	8.562250000	-2.288863000	-1.345878000
16	8.551706000	-0.789694000	1.565739000
6	11.379131000	-1.122383000	-1.289122000
8	12.332594000	-1.594274000	-0.809047000
6	10.166869000	-0.653223000	-3.789409000
8	10.421220000	-0.714124000	-4.931083000
6	10.328034000	1.221994000	-1.632959000
8	10.602767000	2.333615000	-1.381663000
6	5.971005000	-0.855700000	-0.648265000
7	4.844007000	-1.234339000	-0.690325000
6	7.252342000	1.317581000	-0.054104000
8	6.971089000	2.379116000	0.329484000
6	7.913832000	0.314780000	-2.346055000
8	7.390973000	0.874959000	-3.256387000
6	9.234565000	-2.391568000	1.368577000
6	9.814695000	-3.065148000	2.472282000
6	9.237798000	-3.062204000	0.112965000
6	10.359484000	-4.342825000	2.350450000
6	9.787486000	-4.351612000	0.004778000
6	10.348736000	-4.997790000	1.109210000
1	9.825053000	-2.560004000	3.442986000
1	10.794678000	-4.831372000	3.227858000
1	9.777978000	-4.847853000	-0.970583000
1	10.773541000	-5.999977000	1.003226000
6	3.496424000	-1.469404000	-0.623770000
6	2.949090000	-2.797797000	-0.778015000
6	2.628341000	-0.420142000	-0.395083000
6	1.588097000	-2.940104000	-0.746785000
7	1.279069000	-0.547219000	-0.296676000
6	0.714851000	-1.824041000	-0.540233000
1	3.618394000	-3.650863000	-0.902457000

1	3.037781000	0.584170000	-0.314004000
1	1.146650000	-3.934424000	-0.844924000
6	-0.685903000	-1.867961000	-0.553940000
6	-1.467703000	-2.909529000	-1.151713000
7	-1.348684000	-0.754421000	0.012692000
6	-2.829299000	-2.822334000	-1.256500000
6	-2.688167000	-0.659996000	-0.163191000
6	-3.472500000	-1.633241000	-0.757033000
1	-0.946219000	-3.765665000	-1.584116000
1	-3.421622000	-3.599475000	-1.743198000
1	-3.181269000	0.230047000	0.215421000
26	-7.635589000	-0.428115000	-0.766729000
26	-10.079362000	-0.479738000	-1.864174000
16	-8.723608000	-2.398589000	-1.352329000
16	-8.204666000	-0.916729000	1.524673000
6	-5.949991000	-1.050189000	-0.829601000
7	-4.828904000	-1.446028000	-0.849354000
6	-8.002470000	0.107561000	-2.491591000
8	-7.582668000	0.580408000	-3.497244000
6	-7.178686000	1.205238000	-0.357944000
8	-6.882104000	2.300704000	-0.101727000
6	-11.456915000	-1.074887000	-0.945897000
8	-12.391642000	-1.469784000	-0.367454000
6	-10.225892000	1.159472000	-1.289442000
8	-10.378456000	2.267225000	-0.934140000
6	-10.548009000	-0.606604000	-3.547122000
8	-10.970146000	-0.596466000	-4.641070000
6	-9.026674000	-2.458550000	1.430608000
6	-9.504462000	-3.091388000	2.605971000
6	-9.238406000	-3.130173000	0.192618000
6	-10.143471000	-4.330104000	2.568500000
6	-9.876275000	-4.382219000	0.169922000
6	-10.332502000	-4.988863000	1.343734000
1	-9.359782000	-2.582533000	3.563969000

1	-10.496020000	-4.785312000	3.499476000
1	-10.021996000	-4.879950000	-0.793946000
1	-10.830418000	-5.961810000	1.303422000
78	-0.013521000	0.791189000	0.710155000
15	1.705646000	1.963716000	1.699263000
15	-1.645858000	2.299194000	1.388584000
6	-1.208151000	3.517070000	2.713133000
1	-2.007840000	4.271698000	2.740566000
1	-1.296073000	2.949714000	3.653255000
6	1.297018000	3.198128000	3.011372000
1	2.231712000	3.738511000	3.226006000
1	1.032583000	2.636628000	3.921229000
6	-2.376800000	3.270696000	0.003473000
6	-2.315840000	2.721043000	-1.290109000
6	-3.027919000	4.503334000	0.188231000
6	-2.911015000	3.378666000	-2.369654000
6	-3.610521000	5.165951000	-0.896557000
6	-3.559522000	4.602688000	-2.175802000
1	-1.799616000	1.772020000	-1.453583000
1	-3.098925000	4.955267000	1.179409000
1	-2.866943000	2.930189000	-3.365383000
1	-4.115729000	6.122357000	-0.737666000
1	-4.024757000	5.116816000	-3.020941000
6	2.662412000	2.985517000	0.505107000
6	3.821766000	3.675102000	0.904786000
6	2.209243000	3.117623000	-0.818638000
6	4.521890000	4.467682000	-0.007189000
6	2.908870000	3.917411000	-1.727132000
6	4.066243000	4.590815000	-1.324452000
1	4.196978000	3.580568000	1.926462000
1	1.310578000	2.585377000	-1.139602000
1	5.435927000	4.975313000	0.308433000
1	2.550381000	4.006465000	-2.755817000
1	4.619447000	5.205809000	-2.039217000

6	2.880783000	0.838471000	2.555489000
6	4.207330000	0.614414000	2.152277000
6	2.344409000	0.059208000	3.599700000
6	4.984491000	-0.365796000	2.782233000
6	3.126326000	-0.902179000	4.240295000
6	4.448680000	-1.120496000	3.827603000
1	4.648994000	1.184106000	1.333747000
1	1.299441000	0.189016000	3.895892000
1	6.007503000	-0.545397000	2.436943000
1	2.696671000	-1.496984000	5.050954000
1	5.056923000	-1.888666000	4.312700000
6	-3.069751000	1.466806000	2.207942000
6	-2.776582000	0.429998000	3.113001000
6	-4.409046000	1.797671000	1.949589000
6	-3.809624000	-0.274021000	3.736231000
6	-5.437957000	1.087944000	2.575665000
6	-5.143478000	0.048500000	3.461663000
1	-1.736885000	0.150329000	3.301945000
1	-4.664258000	2.574510000	1.227317000
1	-3.570660000	-1.091979000	4.421127000
1	-6.476142000	1.330069000	2.347019000
1	-5.959367000	-0.522652000	3.909850000
6	0.173092000	4.167777000	2.635129000
1	0.350641000	4.609094000	1.638998000
1	0.194446000	5.008675000	3.347978000

Compound 10c

Sum of electronic and zero-point Energies = -12296.848580

Sum of electronic and thermal Energies = -12296.760858

Sum of electronic and thermal Enthalpies = -12296.759914

Sum of electronic and thermal Free Energies = -12296.975251

26	-9.083822000	-1.064008000	-1.066038000

26	-7.129857000	-0.272513000	0.196299000
16	-8.660063000	-1.760401000	1.131136000
16	-6.975044000	-2.001063000	-1.385499000
6	-10.206614000	-2.395935000	-1.507339000
8	-10.928148000	-3.243690000	-1.772153000
6	-10.192742000	0.203568000	-0.460666000
8	-10.895159000	1.020544000	-0.068818000
6	-8.967655000	-0.275089000	-2.669422000
8	-8.882798000	0.212316000	-3.702754000
6	-5.622280000	-0.728103000	1.102458000
7	-4.652839000	-1.087677000	1.677453000
6	-6.575931000	0.895127000	-1.029659000
8	-6.245020000	1.642027000	-1.838435000
6	-7.695764000	1.041696000	1.268144000
8	-8.052741000	1.877951000	1.967234000
6	-7.044269000	-3.437533000	-0.311038000
6	-6.354667000	-4.616442000	-0.588926000
6	-7.827937000	-3.327880000	0.848901000
6	-6.452401000	-5.695581000	0.304642000
6	-7.926401000	-4.398811000	1.735722000
6	-7.231892000	-5.587572000	1.457302000
1	-5.746746000	-4.697810000	-1.493167000
1	-5.915443000	-6.622787000	0.089439000
1	-8.537515000	-4.312148000	2.637274000
1	-7.307760000	-6.429420000	2.149922000
6	-3.340489000	-1.247271000	2.033570000
6	-2.926913000	-1.880820000	3.209322000
6	-2.369667000	-0.697612000	1.185809000
6	-1.559389000	-1.962453000	3.460490000
7	-1.071412000	-0.715698000	1.467801000
6	-0.640935000	-1.385561000	2.574798000
1	-3.657249000	-2.303177000	3.901162000
1	-2.691118000	-0.229331000	0.264508000
1	-1.209587000	-2.474197000	4.356277000

6	0.818061000	-1.519639000	2.699088000
6	1.439978000	-2.376428000	3.613426000
7	1.552657000	-0.829167000	1.781490000
6	2.816328000	-2.569427000	3.561190000
6	2.860570000	-1.057027000	1.675947000
6	3.538145000	-1.922331000	2.554307000
1	0.850604000	-2.912251000	4.356333000
1	3.323317000	-3.229922000	4.266245000
1	3.411296000	-0.549166000	0.887508000
26	7.305023000	-1.624130000	0.708120000
26	7.892909000	-1.580049000	-1.675195000
16	7.309788000	-3.570731000	-0.584274000
16	5.768154000	-0.977731000	-0.939261000
6	5.927070000	-1.933581000	1.839682000
7	4.887568000	-2.087990000	2.388080000
6	8.694763000	-2.229833000	1.667897000
8	9.563570000	-2.632458000	2.296199000
6	7.721122000	0.075076000	1.055645000
8	7.985490000	1.170145000	1.280338000
6	7.591334000	-2.128204000	-3.358948000
8	7.398230000	-2.485689000	-4.429168000
6	8.265277000	0.136382000	-2.019674000
8	8.488406000	1.236486000	-2.252915000
6	9.621326000	-1.975072000	-1.427754000
8	10.723982000	-2.239800000	-1.261083000
6	4.852846000	-2.508242000	-1.151568000
6	3.487297000	-2.533968000	-1.432450000
6	5.566975000	-3.706787000	-0.996420000
6	2.832957000	-3.769505000	-1.564209000
6	4.919507000	-4.934385000	-1.129151000
6	3.544423000	-4.960722000	-1.415254000
1	2.936736000	-1.600762000	-1.549578000
1	1.763408000	-3.791397000	-1.787697000
1	5.477518000	-5.865685000	-1.007330000

1	3.035488000	-5.921912000	-1.520692000
78	0.434248000	0.669571000	0.691541000
15	-1.125183000	1.928442000	-0.516224000
15	2.222530000	2.160978000	0.580065000
6	3.192218000	1.911501000	2.123300000
6	2.504684000	2.077949000	3.339200000
6	4.539999000	1.520566000	2.138299000
6	3.159849000	1.856848000	4.551295000
6	5.192262000	1.304548000	3.356243000
6	4.505990000	1.470194000	4.562012000
1	1.452785000	2.372719000	3.341213000
1	5.082436000	1.356615000	1.207294000
1	2.618311000	1.986960000	5.491405000
1	6.240340000	0.999605000	3.357005000
1	5.018422000	1.295988000	5.511353000
6	-2.522925000	2.414052000	0.566283000
6	-3.737087000	2.860417000	0.011292000
6	-2.399802000	2.322699000	1.964719000
6	-4.816882000	3.169977000	0.842199000
6	-3.479529000	2.644819000	2.790214000
6	-4.693555000	3.055464000	2.230717000
1	-3.848652000	2.965515000	-1.068756000
1	-1.463108000	1.989054000	2.414691000
1	-5.762189000	3.492632000	0.400351000
1	-3.372159000	2.565672000	3.874463000
1	-5.544984000	3.287051000	2.874793000
6	-1.736498000	0.782827000	-1.830353000
6	-3.068625000	0.358832000	-1.964839000
6	-0.775332000	0.311173000	-2.743530000
6	-3.433282000	-0.516660000	-2.992388000
6	-1.142842000	-0.560011000	-3.770263000
6	-2.473025000	-0.977865000	-3.896348000
1	-3.836747000	0.698003000	-1.272533000
1	0.262216000	0.635019000	-2.664443000

1	-4.474721000	-0.836063000	-3.077484000
1	-0.385949000	-0.913099000	-4.474825000
1	-2.759504000	-1.660919000	-4.699698000
6	3.321384000	1.907895000	-0.850473000
6	2.911388000	1.040073000	-1.874029000
6	4.543950000	2.594773000	-0.977812000
6	3.687958000	0.883262000	-3.024262000
6	5.323861000	2.423503000	-2.123162000
6	4.893528000	1.577570000	-3.151317000
1	1.979260000	0.485308000	-1.768261000
1	4.893851000	3.262195000	-0.188400000
1	3.355412000	0.205316000	-3.813499000
1	6.276502000	2.950022000	-2.209510000
1	5.510378000	1.445931000	-4.043551000
26	0.550788000	4.808651000	-0.663872000
6	1.796735000	3.915295000	0.637118000
6	-0.700468000	3.431576000	-1.423603000
6	0.667623000	4.461640000	1.346564000
6	2.481512000	5.013687000	-0.000499000
6	0.415832000	3.602705000	-2.317917000
6	-1.381496000	4.702450000	-1.331350000
6	0.656772000	5.868918000	1.134694000
6	1.770584000	6.206998000	0.310320000
6	0.425874000	4.957897000	-2.749657000
6	-0.677758000	5.631012000	-2.147752000
1	-0.069978000	3.891823000	1.906297000
1	3.359276000	4.947566000	-0.637660000
1	1.144375000	2.842585000	-2.588154000
1	-2.252262000	4.926022000	-0.720712000
1	-0.098222000	6.559463000	1.507492000
1	2.016967000	7.201845000	-0.057742000
1	1.173735000	5.407009000	-3.401321000
1	-0.924601000	6.685729000	-2.259649000

Compound $10c^{2-}$

Sum of electronic and zero-point Energies = -12297.114668

Sum of electronic and thermal Energies = -12297.025157

Sum of electronic and thermal Enthalpies = -12297.024213

Sum of electronic and thermal Free Energies = -12297.247227

26	-9.456974000	-0.803739000	-0.812534000
26	-7.285770000	-0.267433000	0.199567000
16	-8.803107000	-1.698289000	1.259138000
16	-7.503037000	-1.897214000	-1.489199000
6	-10.734344000	-2.002123000	-1.199263000
8	-11.558046000	-2.767047000	-1.424768000
6	-10.352874000	0.483408000	0.036119000
8	-10.925546000	1.310560000	0.590793000
6	-9.441317000	0.135611000	-2.331048000
8	-9.421734000	0.726089000	-3.315086000
6	-5.694520000	-0.935412000	0.899215000
7	-4.676749000	-1.378581000	1.286679000
6	-6.685420000	0.892196000	-1.005142000
8	-6.328311000	1.633003000	-1.809518000
6	-7.566711000	1.020121000	1.395295000
8	-7.739031000	1.844235000	2.177228000
6	-7.564682000	-3.393553000	-0.502398000
6	-7.031775000	-4.603930000	-0.944048000
6	-8.166179000	-3.302914000	0.764029000
6	-7.099197000	-5.732035000	-0.110449000
6	-8.233820000	-4.423051000	1.591801000
6	-7.696663000	-5.642424000	1.147831000
1	-6.563726000	-4.669479000	-1.929274000
1	-6.681613000	-6.681622000	-0.454268000
1	-8.700629000	-4.349783000	2.577096000
1	-7.748771000	-6.521362000	1.795225000
6	-3.360018000	-1.644510000	1.568177000

6	-2.890771000	-2.871471000	2.153731000
6	-2.439215000	-0.661480000	1.249193000
6	-1.545047000	-2.990213000	2.371869000
7	-1.120111000	-0.752113000	1.455023000
6	-0.621517000	-1.943319000	2.030113000
1	-3.588253000	-3.680038000	2.378409000
1	-2.820583000	0.263705000	0.837497000
1	-1.144801000	-3.924495000	2.769293000
6	0.765279000	-2.004972000	2.187806000
6	1.462946000	-2.924382000	3.040866000
7	1.508693000	-1.063657000	1.433170000
6	2.827956000	-2.961906000	3.090926000
6	2.848832000	-1.160935000	1.446927000
6	3.551129000	-2.062159000	2.232960000
1	0.878607000	-3.578442000	3.690788000
1	3.366719000	-3.637291000	3.757458000
1	3.404801000	-0.507160000	0.776447000
26	7.512550000	-1.404706000	0.766859000
26	8.257767000	-1.449362000	-1.569794000
16	7.729372000	-3.424823000	-0.411277000
16	6.050748000	-0.958207000	-1.011471000
6	6.018918000	-1.819024000	1.796954000
7	4.919816000	-2.061803000	2.138665000
6	8.849736000	-1.853416000	1.861043000
8	9.698900000	-2.164955000	2.568256000
6	7.732413000	0.332258000	1.066602000
8	7.882711000	1.461340000	1.228876000
6	8.146682000	-2.131386000	-3.223910000
8	8.093383000	-2.584969000	-4.275963000
6	8.507146000	0.264884000	-1.991648000
8	8.648847000	1.368703000	-2.275792000
6	9.972531000	-1.658225000	-1.127697000
8	11.077016000	-1.794299000	-0.841084000
6	5.255428000	-2.556222000	-1.194893000

6	3.912369000	-2.683749000	-1.546554000
6	6.030296000	-3.695892000	-0.925487000
6	3.340746000	-3.963520000	-1.631518000
6	5.464127000	-4.967062000	-1.011281000
6	4.111045000	-5.096520000	-1.366571000
1	3.310863000	-1.794941000	-1.742013000
1	2.286239000	-4.064380000	-1.899857000
1	6.067195000	-5.852344000	-0.795323000
1	3.663858000	-6.091733000	-1.429095000
78	0.384313000	0.607009000	0.714504000
15	-1.147971000	1.868967000	-0.528104000
15	2.203242000	2.052554000	0.691860000
6	3.167215000	1.863935000	2.254785000
6	2.484385000	2.131347000	3.455815000
6	4.486891000	1.389588000	2.313350000
6	3.117520000	1.946734000	4.685546000
6	5.117751000	1.201888000	3.547957000
6	4.438134000	1.480742000	4.735640000
1	1.445852000	2.470348000	3.430400000
1	5.027436000	1.137104000	1.401486000
1	2.574169000	2.158221000	5.610134000
1	6.141862000	0.821732000	3.572450000
1	4.930451000	1.327561000	5.699427000
6	-2.544186000	2.525519000	0.474849000
6	-3.748740000	2.939605000	-0.122312000
6	-2.420269000	2.580734000	1.873879000
6	-4.816312000	3.370345000	0.669847000
6	-3.484445000	3.026279000	2.661782000
6	-4.687645000	3.412502000	2.062328000
1	-3.866067000	2.912501000	-1.206801000
1	-1.500152000	2.233643000	2.348790000
1	-5.756297000	3.662469000	0.196102000
1	-3.377428000	3.054278000	3.748918000
1	-5.529681000	3.735557000	2.678980000

6	-1.872217000	0.737472000	-1.801078000
6	-3.176591000	0.216388000	-1.760039000
6	-0.998947000	0.290948000	-2.812436000
6	-3.599935000	-0.720425000	-2.709058000
6	-1.426641000	-0.635468000	-3.764146000
6	-2.729909000	-1.147990000	-3.714540000
1	-3.881883000	0.525448000	-0.991052000
1	0.028116000	0.658995000	-2.852156000
1	-4.618470000	-1.113385000	-2.651103000
1	-0.735421000	-0.966045000	-4.543847000
1	-3.061851000	-1.880331000	-4.454867000
6	3.371367000	1.815274000	-0.704753000
6	2.983523000	0.995230000	-1.777372000
6	4.598925000	2.499891000	-0.778715000
6	3.777259000	0.905781000	-2.924650000
6	5.395404000	2.398563000	-1.921629000
6	4.977984000	1.615759000	-3.003227000
1	2.047686000	0.434510000	-1.717324000
1	4.936436000	3.120895000	0.053151000
1	3.459287000	0.268498000	-3.753394000
1	6.350386000	2.926876000	-1.962729000
1	5.604036000	1.539398000	-3.895815000
26	0.632259000	4.702077000	-0.712793000
6	1.831434000	3.834504000	0.672766000
6	-0.640063000	3.328689000	-1.488967000
6	0.687293000	4.423038000	1.317609000
6	2.542948000	4.899330000	0.010658000
6	0.528906000	3.436412000	-2.321625000
6	-1.273292000	4.625180000	-1.472962000
6	0.692139000	5.820939000	1.045519000
6	1.834484000	6.113961000	0.242094000
6	0.614920000	4.776383000	-2.796212000
6	-0.493724000	5.507356000	-2.274511000
1	-0.067908000	3.882099000	1.880723000

1	3.437219000	4.797174000	-0.597697000
1	1.248578000	2.647630000	-2.516479000
1	-2.165415000	4.896697000	-0.914756000
1	-0.067315000	6.532115000	1.367545000
1	2.103658000	7.090216000	-0.159180000
1	1.412701000	5.175707000	-3.420900000
1	-0.696197000	6.565952000	-2.431738000

Compound 10c^{4-}

Sum of electronic and zero-point Energies = -12297.311864

Sum of electronic and thermal Energies = -12297.222591

Sum of electronic and thermal Enthalpies = -12297.221647

Sum of electronic and thermal Free Energies = -12297.444146

26	-8.418021000	-1.559413000	-1.429933000
26	-7.726073000	-0.132554000	0.628735000
16	-8.652316000	-2.344822000	0.720893000
16	-6.626402000	-3.116437000	-1.715720000
6	-9.809989000	-2.323590000	-2.121118000
8	-10.763681000	-2.811763000	-2.578934000
6	-9.191802000	0.011434000	-0.733980000
8	-10.072457000	0.782754000	-0.975397000
6	-7.913382000	-0.694443000	-2.866727000
8	-7.574837000	-0.118162000	-3.816887000
6	-6.122871000	-0.734597000	1.203988000
7	-5.066086000	-1.135703000	1.605922000
6	-7.073718000	1.290776000	-0.137435000
8	-6.665273000	2.262034000	-0.637058000
6	-8.618314000	0.561293000	1.975082000
8	-9.146777000	1.081587000	2.874251000
6	-6.525770000	-3.923507000	-0.168679000
6	-5.568556000	-4.946897000	0.050850000
6	-7.400498000	-3.602498000	0.907426000

6	-5.483712000	-5.623900000	1.266306000
6	-7.302010000	-4.289473000	2.129062000
6	-6.350776000	-5.295327000	2.320855000
1	-4.883451000	-5.200708000	-0.763312000
1	-4.731564000	-6.408076000	1.396172000
1	-7.985389000	-4.022972000	2.941016000
1	-6.287517000	-5.817775000	3.279277000
6	-3.764970000	-1.403481000	1.861371000
6	-3.272140000	-2.717755000	1.997124000
6	-2.838566000	-0.339312000	1.985091000
6	-1.906350000	-2.895396000	2.182179000
7	-1.535690000	-0.522029000	2.128184000
6	-1.052490000	-1.774824000	2.209860000
1	-3.955154000	-3.564277000	1.918530000
1	-3.205320000	0.688778000	1.946017000
1	-1.493349000	-3.903680000	2.252744000
6	0.417104000	-1.936796000	2.281119000
6	1.005068000	-2.889102000	3.138658000
7	1.171212000	-1.161276000	1.470268000
6	2.385352000	-3.047516000	3.161672000
6	2.488515000	-1.331360000	1.461519000
6	3.145675000	-2.254426000	2.291820000
1	0.374095000	-3.482717000	3.801766000
1	2.870833000	-3.759992000	3.831118000
1	3.069207000	-0.720995000	0.768945000
26	7.122827000	-1.883990000	0.792580000
26	7.910671000	-1.865245000	-1.533690000
16	7.062766000	-3.813583000	-0.532798000
16	5.793746000	-1.088292000	-0.960533000
6	5.610435000	-2.174725000	1.798159000
7	4.517794000	-2.344227000	2.204203000
6	8.382147000	-2.592437000	1.843804000
8	9.176295000	-3.064173000	2.524995000
6	7.601478000	-0.213778000	1.194723000

8	7.924575000	0.866844000	1.412114000
6	7.723929000	-2.383306000	-3.240968000
8	7.614301000	-2.725104000	-4.329624000
6	8.443483000	-0.186990000	-1.826410000
8	8.784412000	0.890162000	-2.027782000
6	9.565786000	-2.374482000	-1.099207000
8	10.628423000	-2.706201000	-0.815541000
6	4.767938000	-2.526293000	-1.280325000
6	3.432883000	-2.419433000	-1.667066000
6	5.354754000	-3.787400000	-1.089819000
6	2.679878000	-3.587906000	-1.867112000
6	4.609739000	-4.947990000	-1.292380000
6	3.264170000	-4.841747000	-1.682967000
1	2.977107000	-1.437633000	-1.802063000
1	1.630853000	-3.505682000	-2.161456000
1	5.067306000	-5.928668000	-1.140862000
1	2.675715000	-5.749444000	-1.838336000
78	0.446825000	1.133059000	0.480457000
15	-1.341400000	2.127041000	-0.470995000
15	2.432410000	2.252851000	0.622535000
6	3.486659000	2.162667000	2.142595000
6	3.146763000	2.905926000	3.289839000
6	4.516177000	1.206729000	2.249773000
6	3.814848000	2.699182000	4.500013000
6	5.178688000	0.997258000	3.462265000
6	4.831782000	1.741553000	4.595099000
1	2.348764000	3.650224000	3.243355000
1	4.809174000	0.621532000	1.376246000
1	3.536882000	3.291669000	5.376329000
1	5.969854000	0.245020000	3.518758000
1	5.349666000	1.577293000	5.543693000
6	-2.494420000	2.950978000	0.719571000
6	-3.749118000	3.453753000	0.333358000
6	-2.105066000	3.065891000	2.065807000

6	-4.597544000	4.047255000	1.272873000
6	-2.946596000	3.673868000	3.002160000
6	-4.197287000	4.162689000	2.608518000
1	-4.089264000	3.357724000	-0.698995000
1	-1.145501000	2.649592000	2.382738000
1	-5.581671000	4.401845000	0.958288000
1	-2.630412000	3.749152000	4.046100000
1	-4.864210000	4.622436000	3.342965000
6	-2.472059000	1.241224000	-1.641818000
6	-3.659940000	0.634770000	-1.191629000
6	-2.073835000	1.018717000	-2.974100000
6	-4.430988000	-0.162897000	-2.039396000
6	-2.845950000	0.220092000	-3.824519000
6	-4.024956000	-0.375293000	-3.360661000
1	-4.013463000	0.796077000	-0.174872000
1	-1.154715000	1.469290000	-3.355750000
1	-5.350783000	-0.615544000	-1.660867000
1	-2.522729000	0.065034000	-4.858192000
1	-4.631644000	-0.997980000	-4.022789000
6	3.641474000	2.003806000	-0.755689000
6	3.215380000	1.317968000	-1.904327000
6	4.946799000	2.526969000	-0.725300000
6	4.062050000	1.180243000	-3.009605000
6	5.797180000	2.378242000	-1.824042000
6	5.353814000	1.712047000	-2.972597000
1	2.211095000	0.886059000	-1.923299000
1	5.304438000	3.053869000	0.162318000
1	3.713834000	0.640917000	-3.894447000
1	6.813143000	2.777854000	-1.781112000
1	6.023425000	1.591477000	-3.828359000
26	0.730260000	4.855339000	-0.876814000
6	2.086262000	4.052806000	0.467677000
6	-0.773750000	3.562785000	-1.478310000
6	1.018194000	4.725044000	1.155938000

6	2.729239000	5.027815000	-0.369156000
6	0.336415000	3.516227000	-2.388860000
6	-1.255251000	4.915209000	-1.468126000
6	1.015823000	6.095302000	0.759795000
6	2.068582000	6.280826000	-0.187652000
6	0.526150000	4.818819000	-2.938960000
6	-0.452799000	5.685390000	-2.363998000
1	0.312948000	4.252370000	1.834216000
1	3.553762000	4.835720000	-1.052152000
1	0.941576000	2.638233000	-2.597566000
1	-2.069652000	5.295456000	-0.855052000
1	0.308941000	6.854444000	1.093253000
1	2.305594000	7.207402000	-0.710101000
1	1.305506000	5.106570000	-3.643941000
1	-0.552683000	6.754423000	-2.550588000

Compound 10c^{6-}

Sum of electronic and zero-point Energies = -12297.483099

Sum of electronic and thermal Energies = -12297.393767

Sum of electronic and thermal Enthalpies = -12297.392823

Sum of electronic and thermal Free Energies = -12297.615624

26	-9.020715000	-1.644752000	-1.223398000
26	-7.929389000	-0.328094000	0.926506000
16	-8.875215000	-2.668347000	0.847926000
16	-7.316337000	-3.113264000	-1.979192000
6	-10.534461000	-2.300425000	-1.740361000
8	-11.557534000	-2.733308000	-2.092163000
6	-9.618447000	-0.120673000	-0.241832000
8	-10.514272000	0.667499000	-0.283748000
6	-8.721943000	-0.619828000	-2.612815000
8	-8.491465000	0.058353000	-3.525611000

6	-6.268459000	-0.981050000	1.231768000
7	-5.180537000	-1.435024000	1.449628000
6	-7.414309000	1.138029000	0.143821000
8	-7.126515000	2.144356000	-0.373743000
6	-8.648930000	0.228940000	2.429541000
8	-9.058512000	0.681347000	3.425272000
6	-6.897538000	-4.046722000	-0.558625000
6	-5.870591000	-5.021722000	-0.617423000
6	-7.580398000	-3.879877000	0.678392000
6	-5.544917000	-5.807646000	0.486442000
6	-7.241026000	-4.678650000	1.784669000
6	-6.231925000	-5.640862000	1.699851000
1	-5.326082000	-5.148621000	-1.557786000
1	-4.745638000	-6.550445000	0.404742000
1	-7.780979000	-4.533241000	2.725143000
1	-5.979467000	-6.250703000	2.571609000
6	-3.855485000	-1.695010000	1.574400000
6	-3.337933000	-3.007462000	1.571223000
6	-2.937494000	-0.627688000	1.691690000
6	-1.960761000	-3.174976000	1.645691000
7	-1.622008000	-0.798354000	1.728830000
6	-1.117115000	-2.047761000	1.699562000
1	-4.014460000	-3.857687000	1.477948000
1	-3.313379000	0.396324000	1.737827000
1	-1.530303000	-4.177798000	1.613775000
6	0.356391000	-2.174689000	1.689822000
6	1.016712000	-3.174646000	2.429761000
7	1.048060000	-1.287658000	0.941287000
6	2.403598000	-3.250030000	2.408661000
6	2.376691000	-1.371894000	0.897732000
6	3.116160000	-2.324680000	1.622249000
1	0.438306000	-3.868873000	3.042054000
1	2.944473000	-3.997111000	2.993184000
1	2.906789000	-0.658679000	0.266166000

26	7.275726000	-1.610545000	0.931483000
26	8.881629000	-0.282206000	-0.824965000
16	8.313245000	-3.761288000	0.878579000
16	7.025060000	-1.754027000	-1.368938000
6	5.596873000	-1.974960000	1.301814000
7	4.482542000	-2.341614000	1.559098000
6	7.766837000	-1.382250000	2.595202000
8	8.078435000	-1.214710000	3.700835000
6	7.365375000	0.235708000	0.661593000
8	6.975694000	1.280899000	1.074078000
6	10.012445000	-1.252505000	-1.776009000
8	10.764490000	-1.852992000	-2.433355000
6	8.647764000	1.175816000	-1.764689000
8	8.654887000	2.172667000	-2.382671000
6	10.009091000	0.038108000	0.468122000
8	10.777276000	0.282242000	1.316804000
6	7.631693000	-3.377409000	-1.792412000
6	7.549139000	-3.803179000	-3.129195000
6	8.209675000	-4.225874000	-0.806602000
6	8.039229000	-5.049758000	-3.526172000
6	8.705896000	-5.482652000	-1.235227000
6	8.624271000	-5.889969000	-2.565792000
1	7.096298000	-3.133822000	-3.867150000
1	7.968962000	-5.360609000	-4.572293000
1	9.162936000	-6.142009000	-0.490849000
1	9.016954000	-6.869343000	-2.856663000
78	-0.028532000	0.988425000	0.358931000
15	-1.801148000	1.976440000	-0.628128000
15	1.900045000	2.184328000	0.573735000
6	2.867305000	2.113011000	2.152818000
6	2.330584000	2.711190000	3.311296000
6	4.023766000	1.320361000	2.284945000
6	2.938506000	2.531490000	4.555631000
6	4.631455000	1.142695000	3.532369000

6	4.091860000	1.743063000	4.673855000
1	1.424290000	3.318477000	3.241473000
1	4.487356000	0.854680000	1.415752000
1	2.507865000	3.008990000	5.440703000
1	5.539946000	0.537856000	3.594689000
1	4.567214000	1.603180000	5.648615000
6	-2.938657000	2.859685000	0.540223000
6	-4.148434000	3.438435000	0.119005000
6	-2.590440000	2.940562000	1.900160000
6	-4.985783000	4.085631000	1.032441000
6	-3.423119000	3.594969000	2.813225000
6	-4.623604000	4.169407000	2.381040000
1	-4.458089000	3.360838000	-0.924807000
1	-1.664415000	2.470731000	2.241849000
1	-5.935189000	4.502383000	0.689117000
1	-3.138062000	3.643100000	3.867739000
1	-5.282727000	4.669637000	3.095921000
6	-2.985581000	1.088529000	-1.745265000
6	-4.168951000	0.516006000	-1.239749000
6	-2.629285000	0.810609000	-3.079368000
6	-4.970333000	-0.309164000	-2.031791000
6	-3.433979000	-0.011082000	-3.875655000
6	-4.604134000	-0.577911000	-3.354475000
1	-4.490796000	0.721560000	-0.219492000
1	-1.713369000	1.233627000	-3.500436000
1	-5.883080000	-0.741374000	-1.611919000
1	-3.140628000	-0.211132000	-4.910680000
1	-5.230922000	-1.230973000	-3.967127000
6	3.190508000	1.897375000	-0.717118000
6	2.933042000	0.994933000	-1.763369000
6	4.442161000	2.534327000	-0.664223000
6	3.912320000	0.726120000	-2.726157000
6	5.417860000	2.268125000	-1.625519000
6	5.158524000	1.357591000	-2.654975000

1	1.965381000	0.487181000	-1.807204000
1	4.670857000	3.219511000	0.154263000
1	3.706393000	0.005252000	-3.521883000
1	6.395529000	2.744288000	-1.555043000
1	5.939347000	1.128964000	-3.384605000
26	0.252957000	4.694783000	-1.085741000
6	1.548199000	3.979695000	0.364403000
6	-1.199469000	3.352313000	-1.695488000
6	0.427273000	4.648677000	0.965764000
6	2.212102000	4.942974000	-0.470857000
6	-0.029298000	3.251299000	-2.524914000
6	-1.685424000	4.698743000	-1.814697000
6	0.409376000	6.002337000	0.516417000
6	1.509924000	6.182914000	-0.376040000
6	0.193612000	4.514081000	-3.150137000
6	-0.826432000	5.410611000	-2.706762000
1	-0.304280000	4.182934000	1.619820000
1	3.078021000	4.752044000	-1.100075000
1	0.596482000	2.367121000	-2.622329000
1	-2.540033000	5.117260000	-1.287739000
1	-0.335440000	6.751991000	0.781780000
1	1.754718000	7.096402000	-0.917672000
1	1.019730000	4.758150000	-3.817235000
1	-0.917575000	6.462839000	-2.975687000

Compound 12

Sum of electronic and zero-point Energies = -10004.963930

Sum of electronic and thermal Energies = -10004.888952

Sum of electronic and thermal Enthalpies = -10004.888008

Sum of electronic and thermal Free Energies = -10005.081256

26	8.660770000	-0.747431000	-0.684407000
26	7.489546000	0.850070000	0.767158000

16	8.319396000	1.480085000	-1.322733000
16	6.335740000	-0.696471000	-0.572878000
6	8.981206000	-1.400096000	-2.327082000
8	9.189347000	-1.796765000	-3.380576000
6	10.300604000	-0.338261000	-0.096205000
8	11.350479000	-0.072886000	0.280276000
6	8.640826000	-2.286341000	0.228839000
8	8.594632000	-3.280157000	0.798810000
6	6.039768000	1.935941000	0.906703000
7	5.008740000	2.518217000	0.889183000
6	7.279064000	-0.218170000	2.184013000
8	7.121066000	-0.921029000	3.078034000
6	8.731422000	1.842480000	1.594463000
8	9.513909000	2.494576000	2.118797000
6	5.911252000	0.345927000	-1.971360000
6	4.718113000	0.204227000	-2.678873000
6	6.831929000	1.346539000	-2.322425000
6	4.447702000	1.071856000	-3.749792000
6	6.563729000	2.207685000	-3.385582000
6	5.363294000	2.065124000	-4.100482000
1	4.002834000	-0.572928000	-2.400715000
1	3.516512000	0.960855000	-4.309393000
1	7.280475000	2.986790000	-3.655991000
1	5.150884000	2.737305000	-4.935386000
6	3.642417000	2.631285000	0.803738000
6	2.970316000	3.857600000	0.875335000
6	2.899138000	1.451602000	0.643323000
6	1.580935000	3.847763000	0.802543000
7	1.566422000	1.452605000	0.585422000
6	0.889241000	2.636188000	0.673268000
1	3.526512000	4.788732000	0.992257000
1	3.412109000	0.493512000	0.569766000
1	1.034257000	4.788317000	0.860862000
6	-0.575392000	2.515307000	0.626843000

6	-1.469237000	3.595354000	0.654890000
7	-1.037355000	1.232877000	0.555709000
6	-2.841683000	3.373667000	0.577342000
6	-2.350507000	1.009950000	0.491373000
6	-3.288656000	2.050144000	0.480456000
1	-1.094657000	4.616197000	0.725939000
1	-3.553017000	4.200895000	0.582290000
1	-2.696487000	-0.021340000	0.443843000
26	-6.757299000	-0.335683000	-0.158843000
26	-9.125393000	-0.957764000	-0.363345000
16	-8.372363000	0.420494000	1.361448000
16	-8.182542000	0.757186000	-1.651682000
6	-5.485061000	0.917920000	0.162420000
7	-4.611243000	1.696549000	0.352791000
6	-6.289095000	-1.631598000	0.972691000
8	-5.986714000	-2.470424000	1.697239000
6	-5.936023000	-1.158561000	-1.513872000
8	-5.399076000	-1.673589000	-2.388848000
6	-10.841290000	-0.458996000	-0.545057000
8	-11.930446000	-0.134562000	-0.677940000
6	-8.925473000	-2.110724000	-1.716501000
8	-8.790580000	-2.852150000	-2.580720000
6	-9.353141000	-2.289863000	0.813628000
8	-9.503321000	-3.123598000	1.584849000
6	-8.649873000	2.203101000	-0.695358000
6	-8.920544000	3.436400000	-1.285464000
6	-8.735015000	2.048149000	0.697221000
6	-9.279356000	4.523139000	-0.471276000
6	-9.091507000	3.125480000	1.506535000
6	-9.364561000	4.368923000	0.913240000
1	-8.856839000	3.553596000	-2.369847000
1	-9.495062000	5.490879000	-0.930691000
1	-9.159448000	3.000047000	2.589850000
1	-9.647011000	5.215717000	1.543561000

44	0.395647000	-0.262319000	0.498840000
6	0.206483000	0.118244000	4.843484000
6	2.466739000	-1.840141000	1.846799000
6	3.589808000	-2.655775000	2.037334000
6	4.190728000	-3.264795000	0.938363000
6	3.646248000	-3.056639000	-0.330347000
6	2.539263000	-2.224014000	-0.457687000
7	1.969309000	-1.613856000	0.598657000
1	3.997842000	-2.807297000	3.036242000
1	5.078439000	-3.886344000	1.071367000
1	4.080655000	-3.514327000	-1.220011000
1	2.096404000	-2.022154000	-1.433322000
6	1.737347000	-1.171110000	2.941997000
6	2.067256000	-1.301876000	4.296292000
6	1.296800000	-0.650095000	5.257462000
6	-0.069305000	0.211351000	3.482112000
7	0.674387000	-0.413967000	2.551871000
1	2.917256000	-1.912533000	4.598429000
1	1.544358000	-0.744977000	6.316779000
1	-0.428581000	0.642156000	5.559706000
1	-0.911730000	0.800706000	3.117203000
6	0.618661000	0.321765000	-3.822731000
6	-1.484464000	-1.971560000	-0.955814000
6	-2.586558000	-2.801759000	-1.191073000
6	-3.198155000	-3.450950000	-0.120122000
6	-2.674626000	-3.277310000	1.163130000
6	-1.577013000	-2.437309000	1.333230000
7	-1.006984000	-1.779618000	0.306592000
1	-2.983139000	-2.917291000	-2.198590000
1	-4.075504000	-4.078891000	-0.287321000
1	-3.117390000	-3.768544000	2.030702000
1	-1.152513000	-2.259058000	2.321999000
6	-0.792199000	-1.204947000	-2.009504000
6	-1.059996000	-1.340111000	-3.376826000

6	-0.349058000	-0.568510000	-4.294029000
6	0.842552000	0.407087000	-2.451130000
7	0.155772000	-0.334619000	-1.563161000
1	-1.814330000	-2.046277000	-3.722361000
1	-0.549092000	-0.662550000	-5.363392000
1	1.195041000	0.949380000	-4.503941000
1	1.592851000	1.085071000	-2.041535000

Compound 12^{2-}

Sum of electronic and zero-point Energies = -10005.213595

Sum of electronic and thermal Energies = -10005.137200

Sum of electronic and thermal Enthalpies = -10005.136256

Sum of electronic and thermal Free Energies = -10005.334043

26	-8.991309000	-1.205145000	-0.303153000
26	-7.649503000	0.244850000	1.148081000
16	-7.567709000	-2.110285000	1.338124000
16	-6.968209000	-0.286172000	-1.040338000
6	-9.184771000	-2.705545000	-1.266077000
8	-9.327351000	-3.679983000	-1.855107000
6	-10.341056000	-1.255818000	0.855825000
8	-11.217446000	-1.294960000	1.599394000
6	-9.870553000	-0.066613000	-1.357455000
8	-10.420587000	0.666849000	-2.049065000
6	-5.903099000	0.640580000	1.699850000
7	-4.761520000	0.796848000	1.936195000
6	-8.247138000	1.829450000	0.618899000
8	-8.643966000	2.846472000	0.257563000
6	-8.460426000	0.372315000	2.727313000
8	-8.967493000	0.441944000	3.755917000
6	-5.829848000	-1.637206000	-0.725730000
6	-4.697880000	-1.855016000	-1.510114000
6	-6.107327000	-2.478145000	0.365454000

6	-3.834399000	-2.918186000	-1.201319000
6	-5.248866000	-3.533980000	0.673196000
6	-4.108253000	-3.752168000	-0.116158000
1	-4.484644000	-1.195570000	-2.352600000
1	-2.944142000	-3.086780000	-1.811342000
1	-5.465099000	-4.184668000	1.524037000
1	-3.436846000	-4.580210000	0.124704000
6	-3.417534000	0.939669000	2.103872000
6	-2.817741000	1.138516000	3.399961000
6	-2.574534000	0.902795000	0.982290000
6	-1.461950000	1.264191000	3.485675000
7	-1.254569000	1.030241000	1.054417000
6	-0.623546000	1.201456000	2.309570000
1	-3.451891000	1.178564000	4.288366000
1	-3.012363000	0.762503000	-0.009353000
1	-0.993369000	1.405202000	4.460737000
6	0.772287000	1.265280000	2.300760000
6	1.618193000	1.464410000	3.456102000
7	1.399930000	1.122239000	1.039860000
6	2.979737000	1.465883000	3.352665000
6	2.724011000	1.096713000	0.955463000
6	3.575728000	1.256395000	2.057745000
1	1.152015000	1.621322000	4.429964000
1	3.617978000	1.619154000	4.225495000
1	3.164636000	0.961897000	-0.035094000
26	7.691748000	0.607357000	0.768164000
26	8.850803000	-1.277891000	-0.287622000
16	7.871459000	-1.482801000	1.837002000
16	6.647869000	-0.739534000	-0.861105000
6	6.039375000	1.115642000	1.496467000
7	4.922922000	1.215709000	1.853577000
6	8.791447000	1.351078000	1.954169000
8	9.490867000	1.826013000	2.731940000
6	7.976811000	1.894401000	-0.423873000

8	8.159289000	2.712673000	-1.210781000
6	8.975924000	-3.026040000	-0.665361000
8	9.071837000	-4.146639000	-0.892687000
6	9.392569000	-0.595987000	-1.842986000
8	9.721791000	-0.160760000	-2.853547000
6	10.406148000	-0.892073000	0.490398000
8	11.407679000	-0.650270000	1.000389000
6	5.709171000	-1.848107000	0.193897000
6	4.447197000	-2.323393000	-0.159400000
6	6.273686000	-2.192814000	1.433470000
6	3.741854000	-3.146143000	0.733207000
6	5.575707000	-3.014196000	2.318552000
6	4.303108000	-3.490163000	1.963413000
1	4.006399000	-2.044903000	-1.117832000
1	2.748960000	-3.507351000	0.458001000
1	6.014675000	-3.275549000	3.284505000
1	3.752409000	-4.127664000	2.659587000
44	0.066307000	1.124134000	-0.537011000
6	0.246269000	5.384902000	0.377916000
6	-1.985817000	2.645634000	-1.938051000
6	-3.105161000	2.930527000	-2.741362000
6	-3.623021000	1.951380000	-3.579045000
6	-3.002093000	0.692561000	-3.613401000
6	-1.921159000	0.457157000	-2.773458000
7	-1.428788000	1.392292000	-1.937060000
1	-3.557674000	3.921561000	-2.712778000
1	-4.493511000	2.163195000	-4.203525000
1	-3.359178000	-0.102277000	-4.270241000
1	-1.433581000	-0.518270000	-2.745536000
6	-1.296442000	3.634604000	-1.109308000
6	-1.668695000	4.989958000	-1.022320000
6	-0.897699000	5.870004000	-0.274955000
6	0.557766000	4.035052000	0.265788000
7	-0.189630000	3.172001000	-0.449493000

1	-2.557369000	5.345719000	-1.543578000
1	-1.178772000	6.922930000	-0.201221000
1	0.883567000	6.037216000	0.977158000
1	1.428309000	3.612079000	0.767977000
6	-0.299218000	-3.214643000	-0.393502000
6	1.935185000	-0.213729000	-2.361227000
6	3.001464000	-0.401563000	-3.253128000
6	3.659667000	0.704695000	-3.783013000
6	3.228671000	1.984692000	-3.418565000
6	2.169227000	2.106403000	-2.524993000
7	1.542683000	1.039957000	-1.993098000
1	3.321319000	-1.408155000	-3.521681000
1	4.499408000	0.570084000	-4.467856000
1	3.708327000	2.882287000	-3.812616000
1	1.809489000	3.085013000	-2.203281000
6	1.183932000	-1.309759000	-1.729326000
6	1.363053000	-2.665685000	-2.038381000
6	0.615013000	-3.629712000	-1.367887000
6	-0.447038000	-1.853918000	-0.144612000
7	0.264796000	-0.916535000	-0.799099000
1	2.084298000	-2.963980000	-2.798635000
1	0.747599000	-4.688877000	-1.598000000
1	-0.902206000	-3.930114000	0.167374000
1	-1.161355000	-1.488397000	0.593884000

Compound 12^{4-}

Sum of electronic and zero-point Energies = -10005.368059

Sum of electronic and thermal Energies = -10005.290501

Sum of electronic and thermal Enthalpies = -10005.289557

Sum of electronic and thermal Free Energies = -10005.489609

26	-9.166236000	-1.084105000	-0.116187000
26	-7.839667000	0.669761000	0.977330000

16	-7.639754000	-1.629300000	1.578543000
16	-7.223772000	-0.224424000	-1.103527000
6	-9.292867000	-2.673144000	-0.935035000
8	-9.384533000	-3.692105000	-1.456302000
6	-10.477734000	-1.164781000	1.084763000
8	-11.317295000	-1.228044000	1.868417000
6	-10.136881000	-0.048487000	-1.190906000
8	-10.751701000	0.623287000	-1.893035000
6	-6.079760000	1.142223000	1.451017000
7	-4.928475000	1.235731000	1.679504000
6	-8.403500000	2.169463000	0.222762000
8	-8.768508000	3.142158000	-0.271604000
6	-8.689824000	1.025114000	2.496512000
8	-9.242734000	1.246016000	3.480966000
6	-5.992569000	-1.428585000	-0.600580000
6	-4.853939000	-1.686604000	-1.362218000
6	-6.187482000	-2.079855000	0.629307000
6	-3.904242000	-2.607772000	-0.894823000
6	-5.243822000	-2.997356000	1.092418000
6	-4.099461000	-3.261480000	0.322911000
1	-4.688517000	-1.157348000	-2.302052000
1	-3.002547000	-2.794857000	-1.481771000
1	-5.393198000	-3.498207000	2.052232000
1	-3.352823000	-3.971013000	0.686437000
6	-3.584365000	1.287895000	1.873128000
6	-3.004661000	1.714447000	3.119346000
6	-2.707747000	0.915871000	0.829664000
6	-1.644544000	1.739838000	3.242682000
7	-1.389817000	0.943756000	0.938542000
6	-0.782276000	1.356830000	2.146879000
1	-3.656706000	2.007909000	3.945721000
1	-3.120792000	0.581375000	-0.125532000
1	-1.196525000	2.060941000	4.184478000
6	0.617745000	1.363503000	2.179484000

6	1.432266000	1.761490000	3.305897000
7	1.279674000	0.965447000	0.995210000
6	2.797092000	1.724507000	3.248491000
6	2.599661000	0.903055000	0.956047000
6	3.431994000	1.266585000	2.039634000
1	0.943617000	2.105351000	4.219058000
1	3.410810000	2.032445000	4.098539000
1	3.056927000	0.580890000	0.017168000
26	7.607176000	0.920353000	0.804967000
26	9.135178000	-0.643080000	-0.363882000
16	8.433610000	-1.094808000	1.840463000
16	6.825841000	-0.666389000	-0.743375000
6	5.913208000	1.031080000	1.608094000
7	4.777595000	1.191719000	1.891442000
6	8.543967000	1.923656000	1.930314000
8	9.148185000	2.564494000	2.671872000
6	7.499290000	2.209243000	-0.392277000
8	7.420806000	3.043233000	-1.183552000
6	9.679789000	-2.304867000	-0.742589000
8	10.068895000	-3.363705000	-0.965848000
6	9.370133000	0.114024000	-1.956910000
8	9.509457000	0.592226000	-2.993845000
6	10.564150000	0.173952000	0.298500000
8	11.500240000	0.682553000	0.739097000
6	6.279779000	-1.957145000	0.382537000
6	5.128446000	-2.705499000	0.143838000
6	7.034499000	-2.169969000	1.550421000
6	4.731352000	-3.684368000	1.068404000
6	6.641001000	-3.146789000	2.467128000
6	5.486323000	-3.906537000	2.221011000
1	4.533855000	-2.514127000	-0.749828000
1	3.818860000	-4.256051000	0.885390000
1	7.225921000	-3.307921000	3.376304000
1	5.176175000	-4.666500000	2.943037000

44	-0.009072000	0.647558000	-0.588863000
6	0.016244000	5.021519000	-0.513995000
6	-2.024269000	1.817227000	-2.336738000
6	-3.104434000	1.877827000	-3.266600000
6	-3.429942000	0.775868000	-4.031973000
6	-2.674612000	-0.420755000	-3.891380000
6	-1.672988000	-0.448042000	-2.927477000
7	-1.380146000	0.594000000	-2.133487000
1	-3.655682000	2.812001000	-3.389200000
1	-4.256105000	0.828875000	-4.746415000
1	-2.890183000	-1.311700000	-4.483422000
1	-1.095330000	-1.357929000	-2.741706000
6	-1.459143000	2.931074000	-1.645912000
6	-1.948512000	4.270732000	-1.706798000
6	-1.224332000	5.304270000	-1.152007000
6	0.420502000	3.696023000	-0.428952000
7	-0.281265000	2.666240000	-0.935524000
1	-2.900639000	4.470110000	-2.202640000
1	-1.601256000	6.329694000	-1.201367000
1	0.626612000	5.808828000	-0.068048000
1	1.341140000	3.424646000	0.094618000
6	-0.291389000	-3.535537000	0.612111000
6	1.973400000	-1.051260000	-1.937860000
6	3.059520000	-1.412525000	-2.796894000
6	3.613521000	-0.485709000	-3.654691000
6	3.082329000	0.831335000	-3.702887000
6	2.020957000	1.131464000	-2.853094000
7	1.492596000	0.259909000	-1.982944000
1	3.443534000	-2.433623000	-2.776516000
1	4.451946000	-0.769109000	-4.297296000
1	3.495110000	1.597645000	-4.360755000
1	1.581052000	2.132956000	-2.837836000
6	1.302145000	-1.927917000	-1.040993000
6	1.624884000	-3.306714000	-0.844389000

6	0.846857000	-4.103521000	-0.033762000
6	-0.549798000	-2.186198000	0.414509000
7	0.203707000	-1.380893000	-0.356140000
1	2.498218000	-3.726224000	-1.346714000
1	1.102307000	-5.156793000	0.112498000
1	-0.935336000	-4.123634000	1.268142000
1	-1.399127000	-1.703059000	0.903881000

Compound 12^{6-}

Sum of electronic and zero-point Energies = -10005.555755

Sum of electronic and thermal Energies = -10005.480428

Sum of electronic and thermal Enthalpies = -10005.479483

Sum of electronic and thermal Free Energies = -10005.671435

26	-8.855592000	-1.233056000	0.025003000
26	-6.786649000	0.048937000	1.172792000
16	-6.772155000	-2.179542000	0.708116000
16	-5.623042000	0.417909000	-0.874539000
6	-9.367177000	-2.846251000	-0.401773000
8	-9.678824000	-3.930423000	-0.691769000
6	-10.165847000	-0.981139000	1.161143000
8	-11.123389000	-0.799808000	1.813212000
6	-8.942163000	-0.602328000	-1.616495000
8	-9.136128000	-0.193837000	-2.693772000
6	-5.560207000	1.100179000	1.801638000
7	-4.654057000	1.793657000	2.237965000
6	-8.262853000	0.903573000	0.517779000
8	-8.842191000	1.909433000	0.323821000
6	-7.437803000	-0.204906000	2.785619000
8	-7.829097000	-0.349977000	3.866103000
6	-5.319672000	-1.207774000	-1.467789000
6	-4.570287000	-1.414439000	-2.649375000
6	-5.812899000	-2.351977000	-0.787709000
6	-4.308821000	-2.696544000	-3.132800000

6	-5.540575000	-3.638630000	-1.282028000
6	-4.795957000	-3.821199000	-2.450798000
1	-4.179890000	-0.546878000	-3.185981000
1	-3.711904000	-2.818118000	-4.041351000
1	-5.929763000	-4.504753000	-0.738516000
1	-4.591888000	-4.829816000	-2.821025000
6	-3.293126000	1.734847000	2.435507000
6	-2.732211000	1.658027000	3.727607000
6	-2.417762000	1.745184000	1.335179000
6	-1.358213000	1.524229000	3.853921000
7	-1.088763000	1.628099000	1.465834000
6	-0.539222000	1.488540000	2.711197000
1	-3.384339000	1.662936000	4.603014000
1	-2.819263000	1.818664000	0.323685000
1	-0.914935000	1.415615000	4.844158000
6	0.901380000	1.253257000	2.737403000
6	1.683051000	1.203010000	3.905932000
7	1.486522000	1.084063000	1.511256000
6	3.045554000	0.960376000	3.827098000
6	2.797063000	0.830353000	1.431479000
6	3.636237000	0.757187000	2.561035000
1	1.217256000	1.364544000	4.878552000
1	3.667669000	0.919695000	4.723432000
1	3.223329000	0.689537000	0.439268000
26	6.994118000	0.422644000	0.251389000
26	8.093272000	-1.984992000	-0.145574000
16	6.986381000	-3.172296000	1.642608000
16	6.014833000	-1.340881000	-0.888658000
6	5.819015000	0.452825000	1.540641000
7	4.968948000	0.465999000	2.431392000
6	8.165050000	1.420092000	1.075328000
8	8.931817000	2.100121000	1.629658000
6	6.962733000	1.426482000	-1.191561000
8	6.864054000	2.181581000	-2.080572000

6	8.655329000	-3.568598000	-0.657881000
8	9.089343000	-4.605269000	-0.959437000
6	8.903636000	-1.132853000	-1.458675000
8	9.472781000	-0.632199000	-2.336784000
6	9.093823000	-1.247933000	1.117036000
8	9.858787000	-0.975745000	1.954453000
6	4.842698000	-2.189752000	0.166252000
6	3.473395000	-2.061001000	-0.109595000
6	5.280121000	-2.946088000	1.282284000
6	2.510041000	-2.645421000	0.718330000
6	4.291255000	-3.527920000	2.110528000
6	2.929960000	-3.378705000	1.835993000
1	3.160353000	-1.473040000	-0.974385000
1	1.448500000	-2.516877000	0.495912000
1	4.609902000	-4.106343000	2.982936000
1	2.191470000	-3.840455000	2.498531000
44	0.261089000	1.499979000	-0.117798000
6	1.999654000	5.444392000	0.651387000
6	-0.642860000	3.513705000	-2.008957000
6	-1.326157000	4.087675000	-3.128339000
6	-2.318671000	3.390208000	-3.775962000
6	-2.662335000	2.084604000	-3.322432000
6	-1.954881000	1.555071000	-2.255147000
7	-0.966250000	2.209758000	-1.611676000
1	-1.066097000	5.097313000	-3.450851000
1	-2.846155000	3.838874000	-4.622399000
1	-3.454521000	1.503930000	-3.795498000
1	-2.177688000	0.552222000	-1.885511000
6	0.303513000	4.196873000	-1.199410000
6	0.771358000	5.529042000	-1.427899000
6	1.605520000	6.147613000	-0.525351000
6	1.555558000	4.142757000	0.812121000
7	0.762668000	3.500805000	-0.070846000
1	0.459006000	6.054799000	-2.331809000

1	1.961150000	7.165059000	-0.710181000
1	2.652237000	5.894751000	1.401203000
1	1.871040000	3.562885000	1.681897000
6	-1.511216000	-2.476780000	-0.414525000
6	1.381789000	-0.053897000	-2.297311000
6	2.220575000	-0.402388000	-3.396349000
6	3.316068000	0.371491000	-3.712993000
6	3.597194000	1.528430000	-2.937566000
6	2.731444000	1.849108000	-1.904696000
7	1.647447000	1.112546000	-1.582932000
1	2.013192000	-1.313655000	-3.959304000
1	3.975557000	0.082176000	-4.535350000
1	4.475434000	2.147123000	-3.122351000
1	2.900962000	2.733966000	-1.287947000
6	0.344722000	-0.883502000	-1.766226000
6	-0.079516000	-2.122247000	-2.326097000
6	-0.998521000	-2.910824000	-1.666343000
6	-1.094103000	-1.250611000	0.074360000
7	-0.221783000	-0.446621000	-0.569240000
1	0.330416000	-2.443497000	-3.284891000
1	-1.331915000	-3.855299000	-2.102486000
1	-2.242770000	-3.064725000	0.141092000
1	-1.492861000	-0.866311000	1.015141000

Compound 12$^{7-\bullet}$

Sum of electronic and zero-point Energies = -10005.620115

Sum of electronic and thermal Energies = -10005.544883

Sum of electronic and thermal Enthalpies = -10005.543939

Sum of electronic and thermal Free Energies = -10005.734037

26	-9.119122000	-1.254923000	0.169518000
26	-7.235142000	0.592711000	0.476449000
16	-6.925820000	-1.643957000	1.020348000

16	-5.953517000	0.198913000	-1.520111000
6	-8.949851000	-2.526714000	-1.032156000
8	-8.837044000	-3.398531000	-1.802273000
6	-10.255379000	-1.857241000	1.355666000
8	-11.113587000	-2.260887000	2.047991000
6	-9.866107000	-0.088907000	-0.890383000
8	-10.432118000	0.655968000	-1.600607000
6	-5.823358000	1.092759000	1.476688000
7	-4.809913000	1.257032000	2.084152000
6	-7.698680000	2.163701000	-0.141368000
8	-8.007303000	3.200252000	-0.565547000
6	-8.717395000	0.512004000	1.552129000
8	-9.365490000	0.976840000	2.431827000
6	-5.409293000	-1.454849000	-1.326920000
6	-4.525764000	-2.031351000	-2.274039000
6	-5.813881000	-2.266864000	-0.230059000
6	-4.054263000	-3.336107000	-2.143461000
6	-5.329703000	-3.581286000	-0.109893000
6	-4.457172000	-4.125907000	-1.055624000
1	-4.200081000	-1.420816000	-3.120517000
1	-3.359329000	-3.735897000	-2.887873000
1	-5.652969000	-4.184215000	0.744515000
1	-4.089684000	-5.149523000	-0.940960000
6	-3.462215000	1.249557000	2.344680000
6	-2.950503000	1.144103000	3.674195000
6	-2.535140000	1.286531000	1.285082000
6	-1.593429000	1.019582000	3.857919000
7	-1.219173000	1.180612000	1.457702000
6	-0.697345000	0.998352000	2.743506000
1	-3.641211000	1.141473000	4.520440000
1	-2.895529000	1.383306000	0.258217000
1	-1.194282000	0.918746000	4.868395000
6	0.708413000	0.774277000	2.824820000
6	1.434568000	0.544186000	4.032896000

7	1.402968000	0.774684000	1.618333000
6	2.798030000	0.341830000	4.009976000
6	2.726358000	0.585506000	1.609734000
6	3.490953000	0.372040000	2.769586000
1	0.902710000	0.526258000	4.985612000
1	3.358756000	0.160759000	4.930625000
1	3.220287000	0.596855000	0.636241000
26	6.859577000	0.793391000	0.584014000
26	8.480636000	-1.199271000	-0.541958000
16	7.952030000	-2.987775000	1.046272000
16	6.181255000	-0.920198000	-0.802797000
6	5.750085000	0.387376000	1.887235000
7	4.859317000	0.154176000	2.697367000
6	7.924249000	1.772536000	1.559071000
8	8.642547000	2.413264000	2.218227000
6	6.464275000	2.048916000	-0.572956000
8	6.119599000	2.950065000	-1.237830000
6	9.202702000	-2.464551000	-1.525622000
8	9.746688000	-3.292117000	-2.140009000
6	8.802313000	0.096015000	-1.694648000
8	9.041034000	0.909331000	-2.487489000
6	9.529149000	-0.515954000	0.719814000
8	10.356530000	-0.218967000	1.486942000
6	5.420814000	-2.226897000	0.157430000
6	4.025439000	-2.362629000	0.109789000
6	6.195658000	-3.084795000	0.978895000
6	3.364262000	-3.321948000	0.883331000
6	5.508629000	-4.045933000	1.757521000
6	4.117376000	-4.161609000	1.714723000
1	3.448794000	-1.692964000	-0.531553000
1	2.274966000	-3.400128000	0.838458000
1	6.089852000	-4.709034000	2.405974000
1	3.618808000	-4.913656000	2.334249000
44	0.216802000	1.137424000	-0.048887000

6	1.400315000	5.276386000	0.823319000
6	-0.959968000	3.075764000	-1.908662000
6	-1.689994000	3.576529000	-3.031646000
6	-2.459015000	2.730553000	-3.801591000
6	-2.526777000	1.353261000	-3.467107000
6	-1.799898000	0.912258000	-2.365798000
7	-1.032958000	1.713377000	-1.608717000
1	-1.634551000	4.638848000	-3.275582000
1	-3.016637000	3.121371000	-4.657730000
1	-3.137005000	0.650968000	-4.036286000
1	-1.829918000	-0.137401000	-2.063656000
6	-0.165208000	3.873902000	-1.035075000
6	0.019270000	5.286709000	-1.160839000
6	0.788121000	5.979362000	-0.250696000
6	1.202293000	3.900713000	0.895380000
7	0.467703000	3.201290000	0.016135000
1	-0.456844000	5.815501000	-1.988480000
1	0.923891000	7.059749000	-0.356086000
1	2.018741000	5.780922000	1.567547000
1	1.669994000	3.314474000	1.690296000
6	-0.984296000	-3.081699000	-0.169536000
6	1.679667000	-0.404553000	-2.064374000
6	2.578759000	-0.679616000	-3.140935000
6	3.477244000	0.275098000	-3.566253000
6	3.512487000	1.537699000	-2.918608000
6	2.605798000	1.763517000	-1.888444000
7	1.706124000	0.859474000	-1.470538000
1	2.560431000	-1.661021000	-3.617343000
1	4.172737000	0.049609000	-4.379576000
1	4.240747000	2.305511000	-3.180213000
1	2.593670000	2.720046000	-1.360233000
6	0.774120000	-1.347775000	-1.496784000
6	0.646809000	-2.709291000	-1.913498000
6	-0.216361000	-3.564777000	-1.262928000

6	-0.838667000	-1.745030000	0.186494000
7	-0.011824000	-0.889427000	-0.435512000
1	1.250232000	-3.072679000	-2.746708000
1	-0.306136000	-4.606008000	-1.585471000
1	-1.691986000	-3.714930000	0.366046000
1	-1.429070000	-1.322838000	1.002516000

Mulliken charges and spin densities:

		1	2
1	Fe	-0.577288	-0.030079
2	Fe	-0.424147	0.059144
3	S	-0.116141	0.005899
4	S	-0.271803	0.001252
5	C	0.016325	0.002053
6	O	-0.160510	0.000839
7	C	0.074535	0.001327
8	O	-0.173680	0.000433
9	C	0.058834	0.000698
10	O	-0.182872	0.000156
11	C	0.018474	0.095838
12	N	0.014764	-0.048105
13	C	0.103816	-0.005895
14	O	-0.128933	-0.002978
15	C	0.069449	-0.003113
16	O	-0.226998	-0.002108
17	C	0.003587	-0.000196
18	C	-0.051403	0.000037
19	C	-0.037781	-0.000983
20	C	-0.002666	0.000390
21	C	-0.032399	0.000299
22	C	-0.035521	-0.000707
23	H	-0.035587	0.000205
24	H	-0.020980	-0.000070
25	H	-0.035518	0.000039

26	H	-0.023180	-0.000021
27	C	0.033647	0.212158
28	C	0.014724	0.020894
29	C	0.072142	-0.078937
30	C	-0.089811	0.043114
31	N	-0.231756	0.116444
32	C	0.095483	0.164068
33	H	-0.022387	-0.001831
34	H	0.034694	0.003430
35	H	-0.022128	-0.002901
36	C	0.105849	0.045780
37	C	-0.092464	0.096470
38	N	-0.226857	0.132163
39	C	0.009823	-0.043461
40	C	0.057184	-0.086540
41	C	0.049680	0.194513
42	H	-0.023760	-0.004895
43	H	-0.029348	0.001102
44	H	0.036641	0.002975
45	Fe	-0.335744	0.113147
46	Fe	-0.524589	-0.011499
47	S	-0.264593	-0.000279
48	S	-0.099489	-0.001059
49	C	-0.096029	0.022094
50	N	-0.106252	-0.030656
51	C	0.006405	-0.010920
52	O	-0.164547	-0.005006
53	C	0.054824	-0.009709
54	O	-0.166726	-0.005041
55	C	0.115281	0.000329
56	O	-0.137374	0.000301
57	C	0.088667	-0.000264
58	O	-0.125928	-0.000297
59	C	0.125756	0.001772

60	O	-0.147221	0.000709
61	C	-0.035760	0.001072
62	C	-0.027643	-0.002170
63	C	0.002025	-0.000348
64	C	-0.028083	0.000384
65	C	-0.062062	0.000181
66	C	-0.008815	-0.000961
67	H	-0.007409	0.001585
68	H	-0.007955	0.000484
69	H	-0.041055	-0.000006
70	H	-0.019840	0.000040
71	Ru	0.200792	0.053803
72	C	-0.074774	0.230420
73	C	0.096223	0.107737
74	C	-0.080879	0.072005
75	C	0.032808	-0.012130
76	C	-0.072552	0.223702
77	C	0.093633	-0.084789
78	N	-0.245336	0.151932
79	H	-0.027571	-0.004013
80	H	-0.017949	-0.000647
81	H	-0.015175	-0.011332
82	H	0.054854	0.002941
83	C	0.087033	0.133133
84	C	-0.079861	0.059456
85	C	0.025137	-0.000125
86	C	0.093415	-0.088198
87	N	-0.248488	0.158298
88	H	-0.025799	-0.003585
89	H	-0.018382	-0.001301
90	H	-0.022255	-0.011450
91	H	0.044763	0.002840
92	C	-0.058223	-0.227342
93	C	0.104076	-0.116721

94	C	-0.069611	-0.069821
95	C	0.033600	0.007452
96	C	-0.059459	-0.223425
97	C	0.096854	0.084466
98	N	-0.254740	-0.155746
99	H	-0.026360	0.003926
100	H	-0.017491	0.000901
101	H	-0.026714	0.011121
102	H	0.044517	-0.003230
103	C	0.082498	-0.124484
104	C	-0.082810	-0.064028
105	C	0.026934	0.003198
106	C	0.096958	0.086069
107	N	-0.250211	-0.156449
108	H	-0.025691	0.003689
109	H	-0.017629	0.001146
110	H	-0.021787	0.011343
111	H	0.046074	-0.003541

Sum of Mulliken charges = -5.00000 1.00000

Compound 12^{8-}

Sum of electronic and zero-point Energies = -10005.644457

Sum of electronic and thermal Energies = -10005.569102

Sum of electronic and thermal Enthalpies = -10005.568157

Sum of electronic and thermal Free Energies = -10005.757921

26	10.026370000	0.514329000	0.521597000
26	7.598815000	-0.533999000	0.629495000
16	8.034538000	1.727876000	0.988692000
16	6.783574000	0.057765000	-1.564100000
6	10.571574000	1.670659000	-0.678726000
8	10.966591000	2.456284000	-1.451602000
6	11.190768000	0.654338000	1.814010000

8	12.080699000	0.696429000	2.582778000
6	10.284138000	-0.899906000	-0.460557000
8	10.574306000	-1.826472000	-1.124397000
6	5.946911000	-0.529981000	1.421354000
7	4.841270000	-0.529775000	1.851797000
6	7.549883000	-2.222318000	0.189981000
8	7.505261000	-3.344346000	-0.110161000
6	8.833790000	-0.836405000	1.940815000
8	9.150955000	-1.376646000	2.949967000
6	6.900818000	1.805152000	-1.576149000
6	6.455625000	2.542949000	-2.702834000
6	7.432107000	2.545847000	-0.479781000
6	6.522926000	3.934931000	-2.750321000
6	7.487967000	3.948865000	-0.539520000
6	7.044706000	4.652466000	-1.663216000
1	6.039974000	1.992351000	-3.551462000
1	6.161305000	4.463490000	-3.638211000
1	7.896254000	4.494360000	0.317180000
1	7.101169000	5.744602000	-1.687861000
6	3.494444000	-0.551778000	2.099418000
6	2.954500000	-0.586986000	3.434865000
6	2.582421000	-0.549015000	1.027846000
6	1.598951000	-0.658810000	3.603634000
7	1.262191000	-0.622005000	1.173691000
6	0.698865000	-0.720339000	2.472809000
1	3.632955000	-0.551750000	4.291348000
1	2.966929000	-0.473500000	0.007680000
1	1.180446000	-0.679760000	4.612106000
6	-0.693404000	-0.865749000	2.547326000
6	-1.475729000	-0.939391000	3.764662000
7	-1.388312000	-0.937193000	1.316595000
6	-2.843574000	-1.004394000	3.733170000
6	-2.714890000	-1.012239000	1.307849000
6	-3.516179000	-1.020962000	2.462217000

1	-0.960216000	-0.929841000	4.727505000
1	-3.429860000	-1.041978000	4.655563000
1	-3.203266000	-1.063669000	0.332981000
26	-7.552761000	-0.760863000	0.968310000
26	-8.594350000	-0.049440000	-1.390283000
16	-8.849995000	0.633101000	2.441646000
16	-6.936444000	1.173974000	-0.169506000
6	-5.959958000	-0.919865000	1.827554000
7	-4.882127000	-0.989928000	2.327406000
6	-8.305392000	-2.185741000	1.637548000
8	-8.811777000	-3.131598000	2.087832000
6	-7.175541000	-1.626755000	-0.605389000
8	-6.621419000	-2.521578000	-1.154457000
6	-9.661807000	1.341170000	-1.503855000
8	-10.372039000	2.264605000	-1.609749000
6	-7.987298000	-0.318540000	-3.007818000
8	-7.724096000	-0.518493000	-4.136040000
6	-9.842307000	-1.198014000	-0.991315000
8	-10.701345000	-1.977579000	-0.802865000
6	-7.682807000	2.509705000	0.746173000
6	-7.433469000	3.831052000	0.335840000
6	-8.521095000	2.256136000	1.870262000
6	-8.000431000	4.921727000	1.001443000
6	-9.086160000	3.379824000	2.526184000
6	-8.833028000	4.685506000	2.106075000
1	-6.786204000	3.997133000	-0.531017000
1	-7.793734000	5.941475000	0.664092000
1	-9.734239000	3.205636000	3.390765000
1	-9.284920000	5.524449000	2.645181000
44	-0.161271000	-0.673831000	-0.325940000
6	0.165325000	-5.023559000	-0.122108000
6	1.972501000	-1.733122000	-2.033180000
6	3.102843000	-1.715137000	-2.917879000
6	3.406639000	-0.586328000	-3.644099000

6	2.587504000	0.574020000	-3.526956000
6	1.529481000	0.523036000	-2.623969000
7	1.236692000	-0.546218000	-1.870926000
1	3.727782000	-2.606191000	-3.005576000
1	4.282575000	-0.579964000	-4.299317000
1	2.802377000	1.492245000	-4.076021000
1	0.898465000	1.401278000	-2.453895000
6	1.495476000	-2.856567000	-1.320748000
6	2.076912000	-4.169070000	-1.343520000
6	1.429743000	-5.233985000	-0.762849000
6	-0.321500000	-3.725359000	-0.058716000
7	0.302526000	-2.657892000	-0.587431000
1	3.044069000	-4.313374000	-1.831468000
1	1.881851000	-6.230533000	-0.783578000
1	-0.387990000	-5.840897000	0.344587000
1	-1.254426000	-3.504772000	0.469676000
6	-0.481739000	3.536142000	0.762496000
6	-2.533305000	0.598371000	-1.451601000
6	-3.798823000	0.702769000	-2.107913000
6	-4.228539000	-0.290163000	-2.961591000
6	-3.395524000	-1.418913000	-3.198073000
6	-2.186434000	-1.487999000	-2.516095000
7	-1.766312000	-0.554222000	-1.647540000
1	-4.438241000	1.563934000	-1.913171000
1	-5.205942000	-0.216455000	-3.442675000
1	-3.704519000	-2.232545000	-3.856653000
1	-1.527235000	-2.354273000	-2.625401000
6	-1.952463000	1.589828000	-0.612999000
6	-2.559730000	2.835930000	-0.264010000
6	-1.841230000	3.798566000	0.409895000
6	0.038494000	2.283848000	0.471865000
7	-0.652602000	1.311983000	-0.154198000
1	-3.604281000	3.011640000	-0.528712000
1	-2.310800000	4.749226000	0.679364000

1	0.128190000	4.270828000	1.291880000
1	1.052630000	2.010480000	0.778423000

Computational data of Complex 6 (basal position)

Sum of electronic and zero-point Energies= -8920.319649

Sum of electronic and thermal Energies= -8920.263619

Sum of electronic and thermal Enthalpies= -8920.262674

Sum of electronic and thermal Free Energies= -8920.418566

26	-7.879369000	-1.034007000	-0.225408000
26	-7.876881000	0.689136000	1.534250000
16	-7.320718000	1.184405000	-0.686375000
16	-9.852861000	-0.009754000	0.487390000
6	-8.393635000	-1.587065000	-1.844902000
8	-8.742950000	-1.929910000	-2.881773000
6	-8.229449000	-2.499352000	0.733943000
8	-8.453318000	-3.446239000	1.342561000
6	-6.091824000	-1.441680000	-0.301378000
7	-4.931489000	-1.630649000	-0.341311000
6	-8.352087000	2.326061000	2.102197000
8	-8.649585000	3.371221000	2.465351000
6	-8.293142000	-0.298034000	2.965236000
8	-8.573589000	-0.928767000	3.881880000
6	-10.054523000	1.277283000	-0.748632000
6	-11.305944000	1.725700000	-1.168523000
6	-8.883426000	1.831899000	-1.289904000
6	-11.384231000	2.739136000	-2.137651000
6	-8.959967000	2.838173000	-2.251768000
6	-10.220264000	3.290859000	-2.675098000
1	-12.215444000	1.292602000	-0.745045000

1	-12.363749000	3.093353000	-2.468314000
1	-8.047194000	3.268930000	-2.670413000
1	-10.283517000	4.079799000	-3.428686000
6	-3.563254000	-1.709354000	-0.369089000
6	-2.898255000	-2.942531000	-0.353154000
6	-2.795047000	-0.526953000	-0.409774000
6	-1.508281000	-2.938976000	-0.389478000
7	-1.472196000	-0.538256000	-0.444097000
6	-0.817182000	-1.712829000	-0.441273000
1	-3.464409000	-3.874715000	-0.308333000
1	-3.304453000	0.443084000	-0.414970000
1	-0.971730000	-3.887490000	-0.360020000
6	0.673347000	-1.653847000	-0.493045000
6	1.442993000	-2.759905000	-0.902876000
7	1.249307000	-0.492486000	-0.136278000
6	2.828830000	-2.657657000	-0.930409000
6	2.568833000	-0.380536000	-0.160661000
6	3.411720000	-1.441577000	-0.548011000
1	0.970379000	-3.690533000	-1.217124000
1	3.455377000	-3.493737000	-1.246902000
1	3.010487000	0.579185000	0.129236000
26	7.755677000	-0.843120000	-0.584258000
26	7.850773000	1.616214000	-0.575739000
16	7.582400000	0.383371000	1.394687000
16	9.786640000	0.302515000	-0.692040000
6	5.939109000	-1.119840000	-0.566564000
7	4.773915000	-1.276917000	-0.554446000
6	7.820433000	-1.201951000	-2.332610000
8	7.860649000	-1.439954000	-3.454573000
6	8.311409000	-2.412106000	0.066550000
8	8.694415000	-3.407933000	0.486656000
6	8.548506000	3.136035000	0.078662000
8	8.988584000	4.107651000	0.497292000
6	7.945869000	1.902850000	-2.335843000

8	8.019622000	2.088544000	-3.466135000
6	6.110716000	2.006088000	-0.550146000
8	4.987071000	2.240582000	-0.511225000
6	10.293774000	0.301828000	1.031219000
6	11.631698000	0.263927000	1.420453000
6	9.274041000	0.339843000	1.995988000
6	11.950026000	0.265035000	2.788440000
6	9.588995000	0.341644000	3.353967000
6	10.936654000	0.304415000	3.747394000
1	12.422530000	0.234326000	0.666946000
1	12.997947000	0.235769000	3.097228000
1	8.793342000	0.371663000	4.102451000
1	11.187068000	0.305840000	4.811190000
6	-6.139083000	0.648853000	1.942069000
8	-5.015915000	0.616693000	2.178107000

Complex 6^{2-} (basal position)

Sum of electronic and zero-point Energies= -8920.512394

Sum of electronic and thermal Energies= -8920.456886

Sum of electronic and thermal Enthalpies= -8920.455942

Sum of electronic and thermal Free Energies= -8920.609027

26	-9.053431000	1.216759000	-0.056846000
26	-7.732004000	-1.092422000	0.305801000
16	-7.482794000	0.437491000	-1.524235000
16	-7.339567000	2.535237000	0.953492000
6	-10.015300000	2.654336000	0.107868000
8	-10.661988000	3.616415000	0.228211000
6	-10.340677000	0.413419000	-0.948196000
8	-11.201267000	-0.094651000	-1.532714000
6	-9.260654000	0.197426000	1.428585000
8	-9.778862000	-0.006853000	2.465149000
6	-6.384033000	-1.969227000	-0.321603000
7	-5.371930000	-2.524932000	-0.749674000
6	-9.001186000	-2.301100000	0.155895000
8	-9.750835000	-3.197974000	0.139691000
6	-6.902136000	-0.915017000	1.840558000
8	-6.369389000	-0.908708000	2.880478000
6	-5.931530000	2.223500000	-0.048054000
6	-4.698203000	2.861558000	0.224874000
6	-5.987200000	1.335034000	-1.155075000
6	-3.576171000	2.653284000	-0.579838000
6	-4.855696000	1.144585000	-1.964679000
6	-3.651254000	1.796251000	-1.687673000
1	-4.634367000	3.538107000	1.082500000
1	-2.639143000	3.166905000	-0.343668000
1	-4.924872000	0.455875000	-2.811603000
1	-2.776141000	1.625590000	-2.320579000
6	-4.026955000	-2.244499000	-0.601279000

6	-3.549022000	-1.176510000	0.183089000
6	-3.062704000	-3.042876000	-1.255795000
6	-2.180863000	-0.953229000	0.254956000
7	-1.756238000	-2.827284000	-1.184073000
6	-1.297936000	-1.793308000	-0.453449000
1	-4.254597000	-0.531957000	0.711003000
1	-3.409046000	-3.885519000	-1.866928000
1	-1.807793000	-0.130972000	0.867353000
6	0.173441000	-1.577098000	-0.432599000
6	0.732373000	-0.317414000	-0.124206000
7	0.958192000	-2.629341000	-0.735907000
6	2.111450000	-0.155864000	-0.123073000
6	2.274741000	-2.486814000	-0.739580000
6	2.909134000	-1.266664000	-0.437548000
1	0.093333000	0.539092000	0.091834000
1	2.573031000	0.806192000	0.106997000
1	2.886796000	-3.362377000	-0.986311000
26	7.289230000	-0.967638000	-0.451114000
26	7.604337000	0.461314000	1.530326000
16	9.419287000	-0.373161000	0.298349000
16	7.076136000	1.354626000	-0.569878000
6	5.451766000	-1.115605000	-0.466746000
7	4.278950000	-1.177453000	-0.455803000
6	7.641461000	-1.348012000	-2.161178000
8	7.892052000	-1.582099000	-3.255544000
6	7.443063000	-2.595107000	0.266727000
8	7.538570000	-3.642456000	0.726422000
6	8.375325000	1.883772000	2.308947000
8	8.865567000	2.791835000	2.808065000
6	5.903800000	0.673670000	2.027465000
8	4.804787000	0.816588000	2.326954000
6	7.855841000	-0.806825000	2.761875000
8	8.022903000	-1.620412000	3.554520000
6	8.705968000	1.837708000	-1.149528000

6	8.915368000	2.949561000	-1.964253000
6	9.789258000	1.038020000	-0.749444000
6	10.219507000	3.262194000	-2.381229000
6	11.084172000	1.347492000	-1.163009000
6	11.295783000	2.467231000	-1.983869000
1	8.071142000	3.570318000	-2.273990000
1	10.386219000	4.133681000	-3.019286000
1	11.924499000	0.723322000	-0.849398000
1	12.309825000	2.712712000	-2.309198000

Complex 6^{4-} (basal position)

Sum of electronic and zero-point Energies= -8920.662463

Sum of electronic and thermal Energies= -8920.605356

Sum of electronic and thermal Enthalpies= -8920.604412

Sum of electronic and thermal Free Energies= -8920.763568

26	-7.199849000	1.552252000	0.394976000
26	-7.756040000	-1.128963000	-0.205355000
16	-8.903115000	0.232073000	1.299209000
16	-8.885476000	2.463719000	-1.130545000
6	-6.954079000	3.231892000	0.831344000
8	-6.757509000	4.353612000	1.076622000
6	-6.019831000	0.979644000	1.573796000
8	-5.231254000	0.626862000	2.345064000
6	-6.255793000	0.983097000	-1.012319000
8	-5.513195000	0.894109000	-1.910407000
6	-6.128354000	-1.727646000	0.146386000
7	-4.995302000	-2.120195000	0.336315000
6	-8.421953000	-0.955929000	-1.836802000
8	-8.813236000	-0.945463000	-2.937286000
6	-8.544565000	-2.614099000	0.205380000
8	-9.095871000	-3.599056000	0.505581000

6	-10.375549000	1.700392000	-0.586064000
6	-11.599575000	2.024068000	-1.215258000
6	-10.402115000	0.754238000	0.472629000
6	-12.805922000	1.448463000	-0.811855000
6	-11.624308000	0.194038000	0.878615000
6	-12.824877000	0.527906000	0.244312000
1	-11.588868000	2.744897000	-2.038299000
1	-13.733931000	1.721784000	-1.323005000
1	-11.621861000	-0.527611000	1.700990000
1	-13.764034000	0.073020000	0.571261000
6	-3.645643000	-2.137099000	0.308962000
6	-2.893278000	-3.287946000	0.636158000
6	-2.899389000	-0.974916000	-0.040014000
6	-1.507932000	-3.232439000	0.579323000
7	-1.578780000	-0.933896000	-0.092061000
6	-0.854086000	-2.038149000	0.195734000
1	-3.409405000	-4.203631000	0.933285000
1	-3.452318000	-0.061590000	-0.291130000
1	-0.934135000	-4.119765000	0.850579000
6	0.622602000	-1.951047000	0.083995000
6	1.440861000	-3.104983000	0.040038000
7	1.183258000	-0.723490000	0.005819000
6	2.817137000	-2.987710000	-0.079494000
6	2.494001000	-0.597903000	-0.106213000
6	3.397233000	-1.697778000	-0.158827000
1	1.000059000	-4.102252000	0.075183000
1	3.458330000	-3.870667000	-0.124394000
1	2.907970000	0.417110000	-0.162424000
26	7.569731000	-0.992450000	-0.975815000
26	8.349063000	0.718544000	1.718243000
16	9.718225000	-0.301844000	0.012211000
16	7.202218000	1.339383000	-0.422157000
6	5.896336000	-1.323729000	-0.490001000
7	4.723823000	-1.512174000	-0.278807000

6	7.967172000	-2.684636000	-0.779804000
8	8.255958000	-3.804476000	-0.636161000
6	7.801124000	-0.827256000	-2.722752000
8	7.789510000	-0.872262000	-3.890132000
6	9.528197000	1.955505000	2.174839000
8	10.323267000	2.668307000	2.652840000
6	6.993170000	1.349802000	2.629652000
8	6.064732000	1.773614000	3.189564000
6	8.369037000	-0.761854000	2.653451000
8	8.494777000	-1.700325000	3.343669000
6	8.703640000	1.943418000	-1.193167000
6	8.759542000	3.102726000	-1.967334000
6	9.879821000	1.177560000	-0.994030000
6	9.975329000	3.514436000	-2.544912000
6	11.081413000	1.589296000	-1.568672000
6	11.131385000	2.761293000	-2.347416000
1	7.849216000	3.687817000	-2.123847000
1	10.006266000	4.424870000	-3.150295000
1	11.985761000	0.995970000	-1.408263000
1	12.078884000	3.075785000	-2.794157000

Computational data for the sequential 1e$^-$ reduction of complex 10a

Complex 10a$^{-1\cdot}$

Sum of electronic and zero-point Energies= -10726.425446
Sum of electronic and thermal Energies= -10726.342487
Sum of electronic and thermal Enthalpies= -10726.341543
Sum of electronic and thermal Free Energies= -10726.555015

26	-9.654200000	0.663303000	0.349687000
26	-7.640786000	0.258168000	-0.993536000
16	-8.969879000	2.154363000	-1.330509000

16	-7.536734000	1.187877000	1.168526000
6	-10.724174000	1.789796000	1.249246000
8	-11.414243000	2.512386000	1.810124000
6	-10.777885000	-0.134757000	-0.787600000
8	-11.496959000	-0.650468000	-1.518755000
6	-9.690872000	-0.733252000	1.460612000
8	-9.687836000	-1.621472000	2.187580000
6	-5.955408000	0.835156000	-1.478694000
7	-4.846875000	1.176051000	-1.678913000
6	-7.235165000	-1.365259000	-0.390327000
8	-6.983750000	-2.422749000	-0.013376000
6	-8.181057000	-0.398838000	-2.561800000
8	-8.527010000	-0.802634000	-3.578735000
6	-7.413937000	2.934456000	0.781755000
6	-6.684321000	3.826023000	1.566863000
6	-8.081461000	3.383326000	-0.369188000
6	-6.625536000	5.179576000	1.196639000
6	-8.025058000	4.726947000	-0.736594000
6	-7.291815000	5.626644000	0.054525000
1	-6.164165000	3.471656000	2.460031000
1	-6.055721000	5.881979000	1.810070000
1	-8.546521000	5.073856000	-1.631965000
1	-7.246643000	6.680726000	-0.230405000
6	-3.486387000	1.336267000	-1.721764000
6	-2.855778000	2.440670000	-2.363199000
6	-2.691105000	0.378601000	-1.097368000
6	-1.484018000	2.514408000	-2.331116000
7	-1.355522000	0.444332000	-1.068386000
6	-0.705481000	1.516480000	-1.676950000
1	-3.457142000	3.202679000	-2.861215000
1	-3.176532000	-0.459971000	-0.602074000
1	-0.980641000	3.350740000	-2.815737000
6	0.717790000	1.521054000	-1.596669000
6	1.564751000	2.522912000	-2.153485000

7	1.298585000	0.441691000	-0.935504000
6	2.933632000	2.419888000	-2.082733000
6	2.629531000	0.324643000	-0.901763000
6	3.489999000	1.271746000	-1.448208000
1	1.117585000	3.387195000	-2.643982000
1	3.587200000	3.186317000	-2.502143000
1	3.059897000	-0.549438000	-0.420695000
26	7.633946000	0.072629000	-0.747956000
26	9.916036000	0.613141000	-0.027318000
16	8.765218000	1.970895000	-1.540605000
16	8.055473000	1.139220000	1.297643000
6	5.929449000	0.676488000	-1.115088000
7	4.837527000	1.054729000	-1.335438000
6	8.016848000	-0.824841000	-2.239295000
8	8.270543000	-1.407051000	-3.196068000
6	7.159188000	-1.415174000	0.103963000
8	6.831231000	-2.363684000	0.665406000
6	11.084087000	1.748946000	0.726839000
8	11.824140000	2.466521000	1.227130000
6	10.143349000	-0.884485000	0.914082000
8	10.284987000	-1.847946000	1.522587000
6	10.934358000	-0.005918000	-1.360399000
8	11.589912000	-0.385547000	-2.221517000
6	7.772019000	2.854160000	0.855098000
6	7.240483000	3.782232000	1.749397000
6	8.096563000	3.236984000	-0.456727000
6	7.031019000	5.104767000	1.325728000
6	7.889296000	4.549931000	-0.877636000
6	7.353211000	5.485653000	0.022201000
1	6.990926000	3.481441000	2.769834000
1	6.615508000	5.834851000	2.024721000
1	8.143776000	4.845308000	-1.898446000
1	7.191154000	6.515833000	-0.304727000
78	-0.069869000	-0.896495000	-0.033409000

15	-1.639380000	-2.351379000	0.810725000
15	1.392776000	-2.065593000	1.298211000
6	0.393274000	-2.893323000	2.614186000
1	1.016808000	-3.613670000	3.164584000
1	0.093227000	-2.099409000	3.315735000
6	-0.817964000	-3.556151000	1.962205000
1	-0.516575000	-4.420081000	1.349069000
1	-1.547675000	-3.902166000	2.708737000
6	2.344600000	-3.353589000	0.433649000
6	2.364451000	-3.369085000	-0.971762000
6	3.080346000	-4.312264000	1.154172000
6	3.123769000	-4.328177000	-1.648476000
6	3.835992000	-5.267322000	0.470997000
6	3.860613000	-5.274331000	-0.928947000
1	1.785937000	-2.631409000	-1.533394000
1	3.075343000	-4.309138000	2.247010000
1	3.137113000	-4.335874000	-2.741105000
1	4.409648000	-6.007820000	1.033663000
1	4.454749000	-6.022648000	-1.459581000
6	-2.499538000	-3.361728000	-0.437670000
6	-3.276787000	-4.464423000	-0.035955000
6	-2.378069000	-3.056657000	-1.803988000
6	-3.943382000	-5.233757000	-0.991808000
6	-3.047284000	-3.832593000	-2.755073000
6	-3.832878000	-4.916379000	-2.350709000
1	-3.366778000	-4.723146000	1.021647000
1	-1.760537000	-2.215206000	-2.125637000
1	-4.550890000	-6.084597000	-0.674015000
1	-2.951109000	-3.588768000	-3.815924000
1	-4.356703000	-5.520074000	-3.096181000
6	-2.888217000	-1.456862000	1.805173000
6	-4.260148000	-1.752348000	1.751264000
6	-2.432125000	-0.420157000	2.640246000
6	-5.161307000	-1.025725000	2.533698000

6	-3.339301000	0.299454000	3.421905000
6	-4.704852000	-0.001345000	3.368416000
1	-4.638275000	-2.532219000	1.090073000
1	-1.370320000	-0.162997000	2.667184000
1	-6.227094000	-1.254328000	2.479701000
1	-2.978986000	1.105518000	4.065839000
1	-5.417202000	0.571638000	3.966698000
6	2.569859000	-0.949829000	2.138297000
6	2.041926000	0.197587000	2.758951000
6	3.959706000	-1.147368000	2.105311000
6	2.898512000	1.132567000	3.342827000
6	4.811144000	-0.204999000	2.690108000
6	4.284478000	0.934700000	3.304155000
1	0.962363000	0.371586000	2.767078000
1	4.389313000	-2.019048000	1.609787000
1	2.483302000	2.024240000	3.818771000
1	5.891293000	-0.355556000	2.651381000
1	4.956353000	1.674009000	3.747318000

Complex 10a$^{3-\cdot}$

Sum of electronic and zero-point Energies= -10726.591011
Sum of electronic and thermal Energies= -10726.505971
Sum of electronic and thermal Enthalpies= -10726.505027
Sum of electronic and thermal Free Energies= -10726.730131

26	9.803016000	-2.066142000	0.107149000
26	7.722930000	-0.745003000	0.089409000
16	8.192803000	-2.353202000	-1.563421000
16	7.983208000	-2.646235000	1.459822000
6	10.660684000	-3.644050000	0.031914000
8	11.213205000	-4.648407000	-0.012837000
6	10.756442000	-1.071166000	-1.027619000

8	11.354206000	-0.427880000	-1.768283000
6	10.562380000	-1.280928000	1.518331000
8	11.034947000	-0.773329000	2.434442000
6	5.867282000	-0.868675000	-0.054400000
7	4.709951000	-1.045369000	-0.157928000
6	7.833041000	0.346253000	1.490242000
8	7.890182000	1.057944000	2.391290000
6	8.012270000	0.582745000	-1.054923000
8	8.178358000	1.444798000	-1.798151000
6	7.148880000	-3.877025000	0.457114000
6	6.387278000	-4.904677000	1.012188000
6	7.244543000	-3.741078000	-0.938260000
6	5.715047000	-5.801588000	0.166079000
6	6.578444000	-4.631944000	-1.778903000
6	5.809799000	-5.666171000	-1.220257000
1	6.310613000	-5.002169000	2.097553000
1	5.115340000	-6.605461000	0.599611000
1	6.650610000	-4.517309000	-2.862894000
1	5.284882000	-6.363490000	-1.877544000
6	3.356299000	-1.159981000	-0.277811000
6	2.712234000	-2.404523000	-0.553836000
6	2.553614000	-0.014615000	-0.130779000
6	1.342261000	-2.421382000	-0.672994000
7	1.235584000	-0.034755000	-0.251578000
6	0.574426000	-1.218362000	-0.530113000
1	3.308378000	-3.311800000	-0.673649000
1	3.019304000	0.950039000	0.089454000
1	0.839336000	-3.362695000	-0.898728000
6	-0.847597000	-1.140662000	-0.648407000
6	-1.711729000	-2.279874000	-0.731379000
7	-1.407574000	0.131408000	-0.653654000
6	-3.077714000	-2.134765000	-0.771741000
6	-2.728020000	0.273035000	-0.706034000
6	-3.618489000	-0.812255000	-0.745569000

1	-1.284083000	-3.283324000	-0.730916000
1	-3.745833000	-2.997163000	-0.805295000
1	-3.118480000	1.293379000	-0.702465000
26	-7.985397000	-0.572883000	-0.455358000
26	-9.819111000	-2.158094000	-0.009942000
16	-8.264956000	-2.539961000	-1.719938000
16	-7.865711000	-2.172491000	1.276050000
6	-6.133399000	-0.477422000	-0.675780000
7	-4.965273000	-0.594009000	-0.735878000
6	-8.532484000	0.434532000	-1.818853000
8	-8.881294000	1.075133000	-2.706452000
6	-8.240613000	0.731312000	0.722954000
8	-8.397745000	1.568973000	1.495830000
6	-10.419740000	-3.834897000	0.234222000
8	-10.810258000	-4.903082000	0.383702000
6	-10.643454000	-1.285218000	1.311371000
8	-11.155817000	-0.719602000	2.169822000
6	-10.955171000	-1.508410000	-1.223229000
8	-11.673392000	-1.085853000	-2.014392000
6	-6.898737000	-3.450059000	0.471489000
6	-5.962671000	-4.224370000	1.155941000
6	-7.083675000	-3.620523000	-0.911447000
6	-5.207011000	-5.177034000	0.453535000
6	-6.333126000	-4.566073000	-1.609405000
6	-5.391147000	-5.347022000	-0.919935000
1	-5.816036000	-4.081509000	2.228913000
1	-4.470125000	-5.781257000	0.987806000
1	-6.475188000	-4.689694000	-2.685425000
1	-4.799735000	-6.085644000	-1.466099000
78	-0.050210000	1.892200000	-0.146156000
15	1.021930000	3.719638000	-0.866057000
15	-0.949108000	3.051351000	1.577977000
6	-0.196646000	4.781602000	1.514589000
1	-0.930774000	5.520509000	1.874499000

1	0.608930000	4.750669000	2.263713000
6	0.360753000	5.174889000	0.140579000
1	-0.434968000	5.593028000	-0.495609000
1	1.138920000	5.949520000	0.235953000
6	-2.765980000	3.263174000	1.447631000
6	-3.322606000	4.279037000	0.647821000
6	-3.623038000	2.266839000	1.957568000
6	-4.694976000	4.303567000	0.377124000
6	-4.993217000	2.294595000	1.688962000
6	-5.536635000	3.313671000	0.896119000
1	-2.681987000	5.052380000	0.217222000
1	-3.210990000	1.454447000	2.561650000
1	-5.107967000	5.101478000	-0.246047000
1	-5.641716000	1.511686000	2.089470000
1	-6.607994000	3.329009000	0.685442000
6	1.005661000	4.489524000	-2.554166000
6	1.814027000	5.586839000	-2.902342000
6	0.120272000	3.967787000	-3.508184000
6	1.734346000	6.148746000	-4.179609000
6	0.034348000	4.533032000	-4.785894000
6	0.842268000	5.623525000	-5.123696000
1	2.516822000	6.000137000	-2.173634000
1	-0.498327000	3.107087000	-3.233907000
1	2.369507000	6.999714000	-4.440832000
1	-0.660467000	4.116604000	-5.520148000
1	0.781496000	6.063214000	-6.122814000
6	2.825217000	3.614765000	-0.493240000
6	3.739072000	3.198757000	-1.480617000
6	3.278534000	3.713499000	0.837059000
6	5.062368000	2.892301000	-1.150395000
6	4.602515000	3.405577000	1.167148000
6	5.499531000	2.992175000	0.176079000
1	3.407212000	3.096248000	-2.516804000
1	2.590983000	4.013070000	1.630463000

1	5.756420000	2.562506000	-1.927243000
1	4.933656000	3.482196000	2.206283000
1	6.531136000	2.746385000	0.436260000
6	-0.670191000	2.665657000	3.368115000
6	-1.507513000	3.090329000	4.414947000
6	0.482994000	1.924434000	3.680456000
6	-1.198572000	2.774466000	5.742265000
6	0.800663000	1.622989000	5.008053000
6	-0.043143000	2.043276000	6.042952000
1	-2.408780000	3.667518000	4.194017000
1	1.124420000	1.578691000	2.863308000
1	-1.861117000	3.105694000	6.546791000
1	1.702560000	1.047842000	5.234329000
1	0.196086000	1.797888000	7.081053000

Complex 10a$^{5-\cdot}$

Sum of electronic and zero-point Energies= -10726.789972

Sum of electronic and thermal Energies= -10726.707070

Sum of electronic and thermal Enthalpies= -10726.706125

Sum of electronic and thermal Free Energies= -10726.918603

26	9.049765000	-0.842735000	-0.782748000
26	7.201688000	-0.637557000	1.399235000
16	8.758216000	0.982771000	0.868308000
16	5.610134000	0.642991000	0.209288000
6	8.051041000	-0.960690000	-2.208227000
8	7.435426000	-1.134990000	-3.190539000
6	10.208856000	0.273805000	-1.511204000
8	10.954135000	1.028700000	-1.988949000
6	10.194971000	-2.109635000	-0.404711000
8	10.941648000	-3.004477000	-0.285195000
6	5.789558000	-1.399808000	2.083387000
7	4.785456000	-1.933654000	2.493022000
6	7.650422000	-1.995317000	0.117547000
8	7.221591000	-3.094438000	-0.038783000
6	8.264077000	-1.313677000	2.616535000
8	8.943219000	-1.759303000	3.445358000
6	6.507339000	2.031670000	-0.387622000
6	5.870541000	3.009145000	-1.184786000
6	7.879338000	2.207950000	-0.077465000
6	6.558808000	4.133894000	-1.638836000
6	8.561296000	3.348979000	-0.535067000
6	7.911815000	4.311453000	-1.310848000
1	4.815707000	2.876010000	-1.436837000
1	6.037743000	4.877119000	-2.249374000
1	9.619692000	3.464927000	-0.286205000
1	8.457844000	5.190879000	-1.663070000
6	3.437926000	-2.073825000	2.567565000

6	2.825483000	-3.253261000	3.038738000
6	2.583882000	-1.036773000	2.117583000
6	1.443007000	-3.372956000	2.972484000
7	1.267862000	-1.163960000	2.075723000
6	0.671258000	-2.314247000	2.452557000
1	3.444190000	-4.072672000	3.408911000
1	3.021535000	-0.099662000	1.760239000
1	0.973251000	-4.303008000	3.293003000
6	-0.784172000	-2.407090000	2.217700000
6	-1.580125000	-3.482126000	2.659304000
7	-1.353671000	-1.408152000	1.496713000
6	-2.932292000	-3.539985000	2.345427000
6	-2.653618000	-1.445789000	1.204598000
6	-3.507379000	-2.500573000	1.587408000
1	-1.137690000	-4.281843000	3.253077000
1	-3.551079000	-4.376419000	2.675536000
1	-3.060609000	-0.617362000	0.624653000
26	-6.658900000	-0.560582000	-0.056332000
26	-8.870925000	-0.505733000	-1.396223000
16	-8.637148000	-1.261125000	0.867457000
16	-6.871121000	1.450845000	1.202613000
6	-5.590732000	-1.673394000	0.705618000
7	-4.817991000	-2.493968000	1.192672000
6	-7.224415000	-1.581571000	-1.554856000
8	-6.761382000	-2.401396000	-2.294146000
6	-5.431470000	0.225257000	-1.021898000
8	-4.624373000	0.773866000	-1.652879000
6	-9.815714000	0.917426000	-0.848135000
8	-10.442021000	1.831053000	-0.505128000
6	-8.283677000	0.295700000	-2.833372000
8	-7.894779000	0.813937000	-3.801329000
6	-10.029010000	-1.696796000	-1.989791000
8	-10.808390000	-2.426829000	-2.453964000
6	-8.325138000	1.200991000	2.146720000

6	-8.770588000	2.183638000	3.064214000
6	-9.095802000	0.013382000	2.029360000
6	-9.912132000	1.993815000	3.840173000
6	-10.242256000	-0.169379000	2.820858000
6	-10.657291000	0.809590000	3.725555000
1	-8.193221000	3.107801000	3.158893000
1	-10.224579000	2.773719000	4.541106000
1	-10.815365000	-1.095049000	2.713240000
1	-11.552479000	0.653126000	4.333278000
78	-0.205198000	0.287431000	0.542116000
15	0.229090000	-0.569123000	-1.552351000
15	0.079639000	2.341160000	-0.308331000
6	0.714780000	2.132954000	-2.056622000
1	0.503960000	3.035138000	-2.649080000
1	1.808559000	2.033982000	-1.980204000
6	0.092637000	0.886246000	-2.698157000
1	-0.986755000	1.029536000	-2.862087000
1	0.542178000	0.656565000	-3.675725000
6	-1.436833000	3.363880000	-0.496479000
6	-2.647333000	2.918522000	0.058067000
6	-1.415970000	4.576016000	-1.212274000
6	-3.819224000	3.667473000	-0.095358000
6	-2.585393000	5.324549000	-1.363992000
6	-3.787554000	4.870413000	-0.806217000
1	-2.674099000	1.973364000	0.605159000
1	-0.483210000	4.945274000	-1.646818000
1	-4.756666000	3.301384000	0.331535000
1	-2.559353000	6.264403000	-1.921395000
1	-4.702683000	5.454815000	-0.931101000
6	-0.972478000	-1.810824000	-2.134123000
6	-0.614982000	-3.110532000	-2.532290000
6	-2.335606000	-1.463263000	-2.060798000
6	-1.609977000	-4.042561000	-2.845831000
6	-3.326405000	-2.393976000	-2.378382000

6	-2.961654000	-3.689600000	-2.766227000
1	0.436037000	-3.398138000	-2.596670000
1	-2.626212000	-0.460840000	-1.743162000
1	-1.323579000	-5.051621000	-3.153944000
1	-4.383777000	-2.125374000	-2.311433000
1	-3.736838000	-4.423045000	-3.002035000
6	1.904407000	-1.263229000	-1.750123000
6	2.286750000	-2.315973000	-0.895364000
6	2.859362000	-0.719379000	-2.628806000
6	3.590787000	-2.813093000	-0.924922000
6	4.166010000	-1.213810000	-2.644923000
6	4.535679000	-2.258480000	-1.794188000
1	1.563463000	-2.736100000	-0.192924000
1	2.600433000	0.102542000	-3.298307000
1	3.882277000	-3.613204000	-0.240494000
1	4.917800000	-0.769547000	-3.299836000
1	5.567767000	-2.608247000	-1.777583000
6	1.331153000	3.391879000	0.522093000
6	2.616816000	2.839535000	0.677767000
6	1.049842000	4.643568000	1.094704000
6	3.607788000	3.532737000	1.373524000
6	2.045310000	5.333596000	1.796177000
6	3.323834000	4.783501000	1.935388000
1	2.858850000	1.855565000	0.265392000
1	0.054504000	5.082024000	1.002687000
1	4.594299000	3.075309000	1.471312000
1	1.815643000	6.306828000	2.238065000
1	4.097873000	5.325145000	2.485055000

S.7. References

(1) Sun, Q.; Aguila, B.; Perman, J.; Nguyen, N.; Ma, S., Flexibility Matters: Cooperative Active Sites in Covalent Organic Framework and Threaded Ionic Polymer. *J. Am. Chem. Soc.* **2016**, *138*, 15790-15796.

(2) Sheldrick, G. M., SHELXT - Integrated space-group and crystal-structure determination. *Acta Crystallogr., Sect. A: Found. Adv.* **2015**, *71*, 3-8.

(3) Sheldrick, G. M., Crystal structure refinement with SHELXL. *Acta Crystallogr., Sect. C: Struct. Chem.* **2015**, *71*, 3-8.
